# Facilitative-Competitive Interactions in an Old-Growth Forest: The Importance of Large-Diameter Trees as Benefactors and Stimulators for Forest Community Assembly

**DOI:** 10.1371/journal.pone.0120335

**Published:** 2015-03-24

**Authors:** Andreas Fichtner, David I. Forrester, Werner Härdtle, Knut Sturm, Goddert von Oheimb

**Affiliations:** 1 Institute of Ecology, Leuphana University of Lüneburg, Lüneburg, Germany; 2 Chair of Silviculture, University of Freiburg, Freiburg, Germany; 3 Community Forest Lübeck, Lübeck, Germany; 4 Institute of General Ecology and Environmental Protection, Technische Universität Dresden, Tharandt, Germany; USDA-ARS, UNITED STATES

## Abstract

The role of competition in tree communities is increasingly well understood, while little is known about the patterns and mechanisms of the interplay between above- and belowground competition in tree communities. This knowledge, however, is crucial for a better understanding of community dynamics and developing adaptive near-natural management strategies. We assessed neighbourhood interactions in an unmanaged old-growth European beech (*Fagus sylvatica*) forest by quantifying variation in the intensity of above- (shading) and belowground competition (crowding) among dominant and co-dominant canopy beech trees during tree maturation. Shading had on average a much larger impact on radial growth than crowding and the sensitivity to changes in competitive conditions was lowest for crowding effects. We found that each mode of competition reduced the effect of the other. Increasing crowding reduced the negative effect of shading, and at high levels of shading, crowding actually had a facilitative effect and increased growth. Our study demonstrates that complementarity in above- and belowground processes enable *F*. *sylvatica* to alter resource acquisition strategies, thus optimising tree radial growth. As a result, competition seemed to become less important in stands with a high growing stock and tree communities with a long continuity of anthropogenic undisturbed population dynamics. We suggest that growth rates do not exclusively depend on the density of potential competitors at the intraspecific level, but on the conspecific aggregation of large-diameter trees and their functional role for regulating biotic filtering processes. This finding highlights the potential importance of the rarely examined relationship between the spatial aggregation pattern of large-diameter trees and the outcome of neighbourhood interactions, which may be central to community dynamics and the related forest ecosystem services.

## Introduction

Tree–tree interactions are important structuring mechanisms for forest community dynamics, and the outcome of these biotic interactions has already been identified to vary across environmental gradients and tree growth stages (e.g., [1–7]). These interactions can be competitive or facilitative and complementary and the direction of biotic interactions may shift with different environmental conditions [7,8,9,10]. Generally, interactions shift towards facilitation as stress increases [11]. For example, if the availability of a given soil resource declines along a spatial or temporal gradient, then facilitation or complementarity could increase if the plants interact in ways that improve the availability or uptake of that resource [7]. Or, as productivity and leaf area increases, competition for light is also likely to become more intense and complementarity can increase if the plants interact in ways that improve light absorption [12]. Moreover, competitive interactions often become less severe in mixed-species communities (e.g., [5,13]), and the negative effects of competition on adult tree growth are on average greater for shading than for crowding [14,15]. In monospecific stands, neighbourhood interactions affecting growth dynamics are determined by factors other than species identity. It has been suggested that the spatial arrangement of trees plays a key role in regulating the intensity of inter–tree competition within structurally diverse old-growth forests [16].

The competitive ability of plants is strongly related to their size, and competitive interactions among trees can be size-asymmetric or size-symmetric [17]. There is more or less consensus that competition for light among terrestrial plants is strongly size-asymmetric [18,19], particularly in later successional stages [20]. In contrast, competition for belowground resources (e.g. water and nutrients) can be size-asymmetric or size-symmetric [19,21–26]. In addition to the importance of differentiating between the modes of competition (e.g. above- vs. belowground and symmetric vs. asymmetric), the potential interactions between above- and belowground competition have received increasing attention in plant ecology, such as whether these effects are additive (e.g. the summation of single effects) or non-additive (e.g. antagonistic or synergistic interaction) [27]. Next to competition many studies increasingly stress facilitation or complementarity as an important driver for community dynamics (e.g., [11,28,29,30]). Most of these findings, however, refer to mixed-species communities or tree seedlings [7,11,31], but mechanisms of facilitative or complementarity interactions between adult trees in monospecific stands are poorly understood.

In this study, we aim to determine how interactions between adult trees are related to above- and belowground processes at the intraspecific level. To answer this question, we used growth data from a long-term (> 50 years) unmanaged old-growth European beech (*Fagus sylvatica*) forest, encompassing a large range of tree sizes (diameter at breast height, DBH 7–116 cm) and age classes (35–240 years). More specifically, we asked (i) whether above- or belowground competition has a stronger effect on tree radial-growth, (ii) whether competitive interactions vary with tree size, and (iii) whether the effects of above- and belowground competition are additive (i.e. the summation of shading and crowding effects) or non-additive (i.e. antagonistic or synergistic interaction).

## Materials and Methods

### Study area

This study was conducted in an 8 ha (200 m × 400 m) permanent plot of an old-growth forest (‘Serrahn’) located in the core zone of the Müritz National Park (Mecklenburg-Western Pomerania, NE Germany, 53° 20’ N, 13° 12’ E). The predominant forest communities in the national park can be assigned to oligotrophic beech forests (*Luzulo-Fagetum*) on dystric cambisols and podzoluvisol soils, and to mesotrophic beech forests (*Milio-Fagetum*) on luvisols. Soils are developed on a parent material of loamy sand and the main humus type is moder. 268 ha of the Serrahn forest are part of the UNESCO World Natural Heritage Site “Primeval beech forests of the Carpathians and the ancient beech forest of Germany” and represent a prime example of natural beech forest dynamics. The climate is suboceanic-subcontinental with annual means for precipitation of 593 mm and for temperature of 7.8°C [32]. Elevation is approximately 100 m a.s.l.

### Forest history and structure

**Fig 1:**
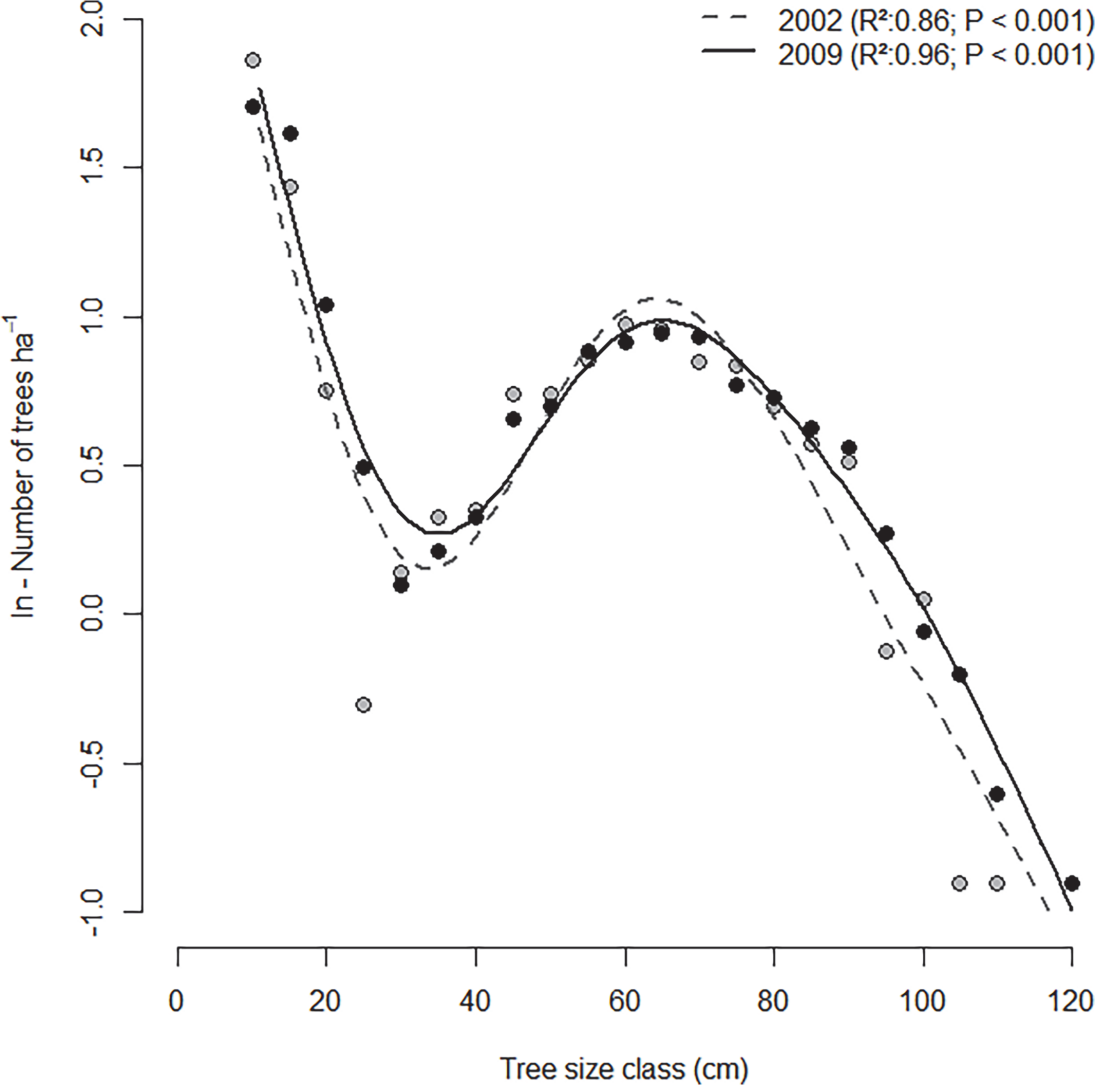
Stand structure of the investigated old-growth beech forest. The x-axis represents the upper boundaries of the tree size (DBH)-class. Regresssion lines were obtained by fitting generalized additive models (*gam* function in R, with five degrees of freedom). Grey dots: investigation year 2002; black dots: investigation year 2009.

The Serrahn forest is characterised by a long (>450 years) continuity of forest cover [33]. From the beginning of the 19^th^ century the Serrahn forest was used as a game park with low intensity silvicultural interventions. In 1960, it was declared a forest nature reserve and management ceased. During the last 40 years, stand structure became more heterogeneous over small spatial scales by shifting from mono-layered to multi-layered stands. These changes were mainly driven by increasing mortality rates of canopy trees in the late 1960s, which caused numerous canopy gaps and created conditions conducive to regeneration over large spatial scales. As a result the volume of dead wood considerably increased from 1967 to 2002 in the permanent plot (4 to 107 m^3^ ha^−1^) [32]. Thus, the current rotated sigmoid diameter distribution (Fig. 1) is mainly a function of self-thinning and mortality processes of old trees [34].

The canopy is dominated by *F*. *sylvatica* (96%). The other 4% is composed of about 3% Scots pine (*Pinus sylvestris*) and 1% Sessile oak (*Quercus petraea*). The understorey consists almost entirely of *F*. *sylvatica* (S1 Table). The age of the overstorey trees varied between 200 and 240 years with maximum values of 116 cm in diameter at breast height (DBH) and 44 m in height. The mean structural characteristics (initial conditions in 2002) are as follows [32]: stand volume 605 m^3^ ha^−1^, stand basal area 32.72 m^2^ ha^−1^ and stand density 263 stems ha^−1^. The mean height of the overstorey and understorey trees was 34.3 m and 11.2 m, respectively. The top height (the average height of the 20% largest-DBH trees) amounted to 38.4 m in the overstorey, and to 16.5 m in the understorey.

**Fig 2:**
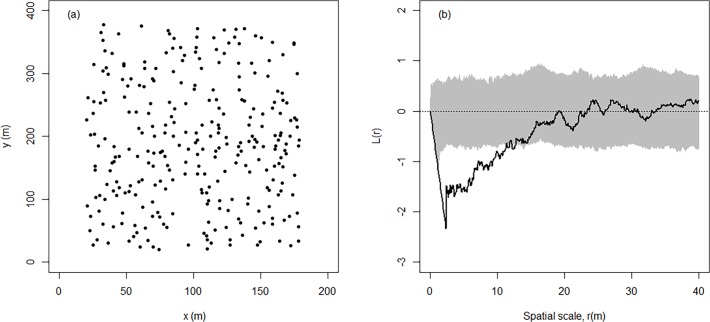
Spatial pattern (a) and corresponding spatial analysis (Ripley’s *L* function; (b)) of large-sized (DBH > 60 cm) beech trees in 2002. Values of *L*(r) above the 95% confidence envelope (determined by 199 Monte Carlo simulations; grey area) indicate spatial aggregation, those within the envelope indicate spatial randomness and those below the envelope indicate spatial regularity. The spatial tree pattern was analysed in R using the package *spatstat*.

**Fig 3:**
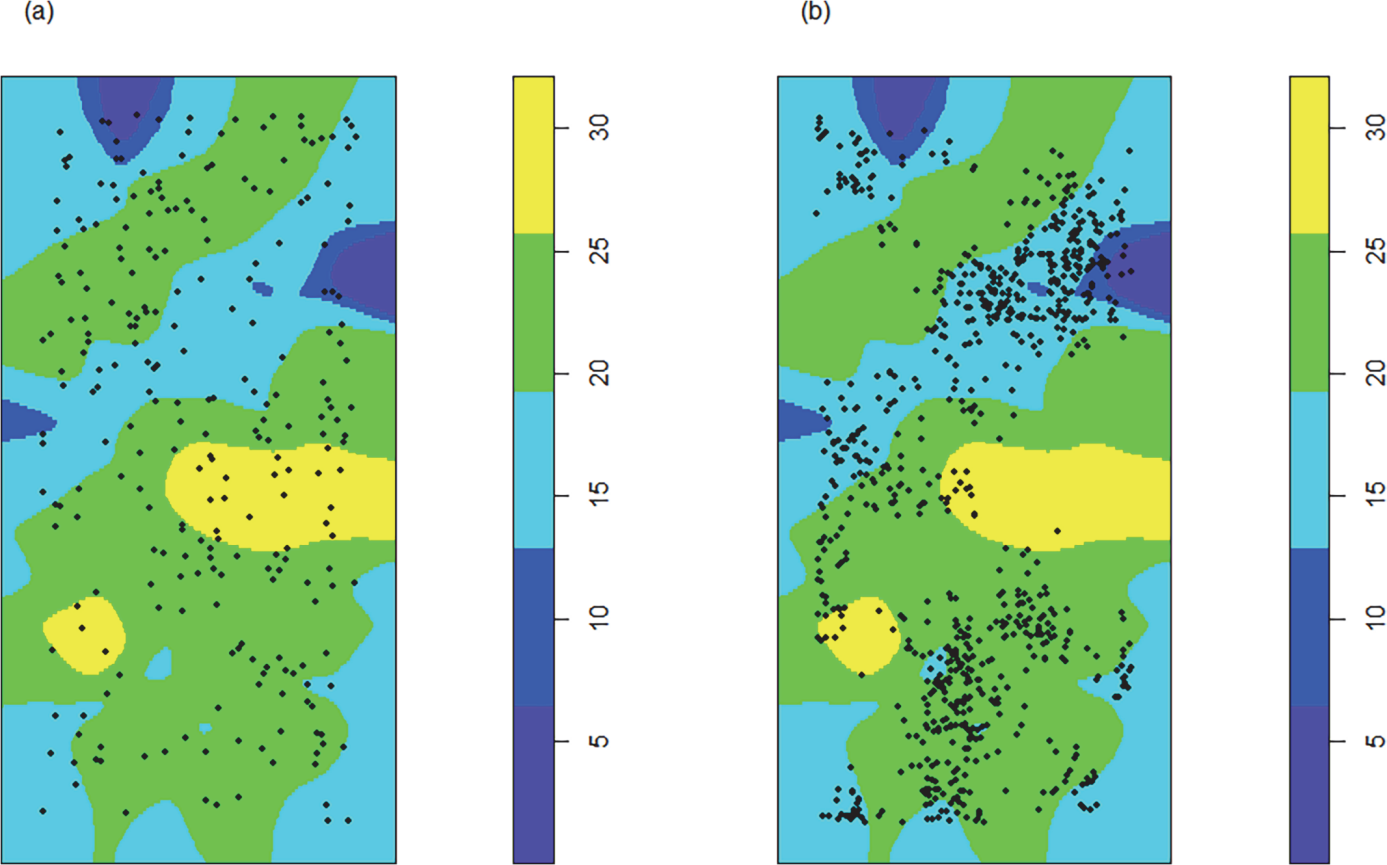
Local neighbourhood densities of large-sized (DBH > 60 cm) beech trees within a spatial distance of 20 m. Yellow and dark blue colours indicate highest and lowest densities of large-diameter trees. Tree densities were obtained by using the *localL* function (R package *spatstat*) with r = 20 m. Black dots indicate the initial spatial pattern of (a) dominant and co-dominant canopy beech trees with a DBH ≤ 60 cm and (b) beech trees growing in the understorey of the 8 ha study plot.

Another old-growth feature is the high abundance of large-sized (>60 cm in DBH) beech trees, which account for 40 stems per hectare (57% of the canopy dominants). Those trees were regularly distributed in the study plot at spatial scales of approx. 13 m, whereas for neighbourhood scales > 13m the tree spatial pattern became more random (Fig. 2). As a result the impact of large-diameter (> 60 cm) trees on the local growing conditions within a neighborhood scale of 20 m was almost equally high for all canopy dominants with a DBH ≤ 60 cm (Fig. 3A). In contrast, understory trees tended to aggregate in areas with low neighbourhood densities of large-diameter trees (Fig. 3B).

### Growth data

The research permission was provided by the administration of the Müritz National Park, Mecklenburg Vorpommern, Germany. No specific permissions were required for our activities. Our field studies did not involve any endangered species.

For all living trees with a DBH ≥ 7 cm in the study plot, stem diameter at 1.30 m, species, spatial position and crown class (dominant, co-dominant and suppressed) [35] were recorded (S2 Table). Annual basal area growth (BAI) was determined from two DBH measurements in 2002 and 2009, which represent a seven year growing period. An allometric equation describing tree height as a function of DBH was calibrated based on a subset of 243 height measurements of *F*. *sylvatica*. For *P*. *sylvestris* and *Q*. *petraea* the height of all trees within the study plot was recorded. Height measurements were determined with a Forestor VERTEX Hypsometer (Haglöf, Sweden).

Growth analyses focused on 545 dominant and co-dominant canopy beech trees (target trees). To account for edge effects, only target trees within a buffer zone of 20 m (see below) to the borders of the 8 ha plot were considered. The minimum radial distance of the study plot to forest edges was 150 m. As a result, the core zone amounted to 5.76 ha, and the buffer zone to 2.24 ha. All other individuals (*n*_*total*_ = 2770) were considered as neighbourhood trees (Table 1).

**Table 1:** Summary statistics of the investigated old-growth beech forest in north-eastern Germany.

	Overstorey trees		Understorey trees	
	Mean (SD)	Min.–Max.	Mean (SD)	Min.–Max.
Diameter at 1.30 m (cm)	61.5 (15.9)	25.7–115.5	9.7 (2.8)	7.0–22.7
Tree height (m)	35.4 (3.5)	24.9–43.7	13.2 (3.4)	7.9–24.0
Basal area growth (cm^2^ year^−1^)	31.3 (25.3)	0.7–178.7	5.0 (5.4)	0.2–36.1
Basal area all trees (m^2^ ha^−1^) ^A^	33.8 (7.6)	17.0–60.1	29.7 (7.2)	9.4–60.2
Basal area larger trees (m^2^ ha^−1^) ^A^	20.7 (12.0)	0.0–52.7	28.1 (7.7)	6.1–60.0
Crowding index ^A^	0.56 (0.13)	0.28–1.00	0.49 (0.12)	0.10–1.00
Shading index ^A^	0.35 (0.20)	0.00–0.88	0.47 (0.13)	0.16–1.00
n_trees_	545		815	

Values refer to the initial growing conditions in the core zone (5.76 ha) in 2002 of the modeling data set.

^A^ values refer to a neighbourhood radius of 20 m

### Competition indices

For distinguishing between above- (shading) and belowground (crowding) competition, we applied two competition indices (CI) according to [23]: An index of shading (CIS) was calculated as the total initial basal area of trees larger than the target tree (BAL) within a specified radius of the target tree. This index assumes that competing trees intercept light in relation to their stature, which typically results in a disproportionally higher light interception of larger trees compared to smaller neighbours [17]. An index of crowding (CIC) was calculated as the total initial basal area of all trees (BA) within this radius. This index assumes that all trees irrespective of their size compete for belowground resources (e.g. nutrients) and represents a proxy measure of belowground competition among trees when used in the same model as the other competition index that accounts more for aboveground competition [23]. In this context, it is worth mentioning the indirect assessment of belowground competition in our study, because we could not directly relate growth rates to measured root parameters such as fine root biomass or productivity. However, distant-dependent and distant-independent indices for crowding are assumed to act as proxies for belowground competition in tree growth studies, which account for both shading and crowding effects (e.g., [5,14,15,23]). To account for the distance-dependency of competition effects, we used a fixed radial distance approach. BAL and BA were computed for different radii (10, 15 and 20 m) and any tree within this distance was included as a neighbour. The optimum neighbourhood radius was determined by calculating the *R* ^2^ of the relationship between ln(BAI) and competition effects (BAL and BA). The area with a 20 m radius explained the highest amount of variation in BAI (*R* ^2^_10m_: 0.15; *R* ^2^_15m_: 0.22; *R* ^2^_20m_: 0.31) and was therefore selected for further analysis. To facilitate comparisons between shading and crowding effects, the competition indices were standardised by:

CI-shading(CISi)=BALi/BALmaxCI-crowding(CICi)=BAi/BAmax

where BAL_*i*_ and BA_*i*_ are the cumulative basal area of trees larger than the target tree and all neighbours within a 20 m radius of a target tree *i*, and BAL_*max*_ and BA_*max*_ are the maximum values for BAL_*i*_ and BA_*i*_ recorded in the study plot. Thus, CIs vary between 0 and 1 and indicate the minimum and maximum neighbourhood interactions observed for any target tree.

### Growth model

To assess the size and competition dependence of radial growth of dominant and co-dominant canopy beech trees, we applied a parametric growth function using a generalised least squares framework (GLS) [36]. This weighted linear regression approach was preferred, because it retains the structure of the data while accounting for a heteroscedastic variance and correlated within-group errors, and thus avoids biased inferences associated with logarithmic transformations [37].

Basal area growth of target tree *i* (growth_*i*_) was modelled as a functional relationship between tree size and the tree’s competitive status:

$${\text{growth}}_{i}=\alpha +\beta_{1}{\text{DBH}}_{i}+\beta_{2}{\text{DBH}}^{2}{\;}_{i}+\beta_{3}{\text{CIS}}_{i}+\beta_{4}{\text{CIC}}_{i}$$

where *α* is the mean basal area growth rate and *β*_1,2,3,4_ are estimated parameters of initial tree size (linear: DBH; non-linear: DBH^2^), shading (CIS, aboveground competition) and crowding effects (CIC, belowground competition). The importance of above- and belowground processes for basal area growth was assessed by fitting several alternative models accounting for size or size and competition effects. Moreover, we considered interaction terms between explanatory variables (Table 2).

**Table 2:** Model selection statistics.

Predictor	ΔAIC	*w*_*i*_	*R* ^2^
DBH	58.0	0.000	0.25
DBH + DBH^2^	55.4	0.000	0.26
DBH + CIS	9.7	0.004	0.31
DBH + DBH^2^ + CIS	26.6	0.000	0.33
DBH + CIC	11.2	0.002	0.30
DBH + DBH^2^ + CIC	25.5	0.000	0.34
DBH + CIS + CIC	11.6	0.002	0.31
DBH + DBH^2^ + CIS + CIC	13.2	0.001	0.30
DBH + CIS + CIC + DBH x CIS	13.6	0.001	0.31
DBH + DBH^2^ + CIS + CIC + DBH x CIS + DBH^2^ x CIS	10.1	0.004	0.30
DBH + CIS + CIC + DBH x CIC	11.1	0.002	0.32
DBH + DBH^2^ + CIS + CIC + DBH x CIC+ DBH^2^ x CIC	9.9	0.004	0.30
DBH + CIS + CIC + CIS x CIC	**0.7**	**0.400**	**0.33**
DBH + DBH^2^ + CIS + CIC + CIS x CIC	**0.0**	**0.576**	**0.31**

To address the skewed response and heteroscedasticity of the growth data, the residual error of the *i*-th target tree (*ε*_*i*_) was modelled using a variance function based on the power of tree size [36].

$$\text{var}\left(\varepsilon_{i}\right)=\sigma^{2}{\left|{\text{DBH}}_{i}\right|}^{2\delta }$$

where *δ* is a parameter to be estimated, which allows the variance to increase with tree size. Moreover, preliminary analyses indicated strong spatial correlation of the residuals. We therefore additionally included an exponential correlation structure in the variance-covariance terms [38]:

$$\gamma \;\left(s,\rho \right)\;=\left\{ \begin{array}{ll} c_{0}+\left(1-c_{0}\right)\left(1-e^{\frac{s}{\rho }}\right), & \text{if}\;s>0\\ 0\;, & \text{if}\;s=0 \end{array}\right.$$

where *ρ* is the estimated range, *s* the estimated distance and *c*_0_ the estimated nugget effect.

Models were selected based on the Akaike Information Criterion (AIC) and maximum likelihood (ML) estimations. Parameter estimates of the best-fitting model were based on the restricted maximum likelihood (REML) method [38]. Only models with an AIC difference (ΔAIC) ≤ 2 (compared with the best-fitting model) were considered as models with substantial support [39]. Models were fitted using the *gls* function from the *nlme* package in R [40].

The 14 candidate models describing basal area growth of dominant and co-dominant canopy beech (*Fagus sylvatica*) trees as a function of initial tree size (diameter at breast height, DBH), aboveground (shading, CIS) and belowground competition (crowding, CIC). The best-fitting models are highlighted in bold. ΔAIC is the difference in AIC (Akaike Information Criterion) with respect to the best-fitting model (lowest value of AIC). The Akaike weight (*w*_*i*_) is the relative likelihood of each model to be the best-fitting model, given the complete set of candidate models. *R* ^2^ is the variance explained by the model.

### Competition effects

We analysed changes in competition effects with various levels of shading and crowding by predicting the decline in potential growth of a target tree (expressed as the growth rate in the absence of competitors) as a function of the degree of competition. This allowed us to test whether target trees are more sensitive to changes in above- or belowground competition.

To more fully understand the mechanisms of biotic interactions, we further analysed how the intensity of tree–tree interactions was affected by competition. The intensity of competition was quantified for each target tree using the log response ratio [41]:

$$\text{LnRR}=\text{ln}\;\left({\text{G}}_{-\text{N}}/{\text{G}}_{+\text{N}}\right)$$

where G denotes the radial growth of a target tree either in absence (−) or presence (+) of local neighbourhood competitors. Positive LnRR-estimates indicate competition, while negative estimates imply that tree–tree interactions are facilitative. In the case of G_−N_, CI was set at 0. In the case of G_+N_, we used the average value of CIS and CIC (see Table 1) to account for potential differences in the strength of each competition mode (shading/crowding). G_−N_ and G_+N_ were predicted for every target tree based on our best-fitting model and LnRRs were calculated separately for each mode of competition. To evaluate changes in the response of neighbourhood interactions at various levels of above- and belowground competition, we predicted LnRRs at low (CI of 0.1) and high (CI of 0.6) levels of competitive stress. We predicted changes in LnRR as a function of tree size to further analyse tree size-related changes in the outcomes of competition. We distinguished between (i) medium-sized trees: DBH 30–60 cm, and (ii) large-sized trees: DBH 61–100 cm. Differences in LnRR between the levels of competition (high/low) were tested by analysis of variance (ANOVA). All statistical analyses were performed using R [40].

## Results

The minimum adequate models (MAM) according to the AIC included a tree size effect and interacting effects of shading and crowding (Akaike model weights of 0.40 and 0.58; Table 2). Thus, both above- and belowground competitive processes drive changes in individual tree growth patterns. However, comparisons of ΔAIC and *R* ^*2*^ indicated that the simpler MAM containing a linear size effect had substantially greater support than the MAM including a marginally significant non-linear response of basal area growth with tree size (DBH^2^: *L* = 2.71, *P* = 0.10; ΔAIC for the MAM with a non-linear size effect was only 0.7 points lower than for the model with a linear size effect; Table 2). Consequently, the model with a linear BAI-DBH relationship was considered as the best-supported growth model (Table 3). Simpler, alternative models that excluded the effects of either competition or the interplay between shading and crowding showed much larger AIC values. Graphical validation plots indicated unbiased estimates (S1 and S2 Figs). The best-supported model explained 33% of the variance in BAI, and the mean prediction error was −1.87 cm^2^ year^−1^.

**Table 3:** Parameter estimates of the best-supported growth model for dominant or co-dominant canopy beech (***Fagus sylvatica***) trees obtained by generalized least squares (GLS) regression.

	Estimate	SE	*P*-value
*Fixed effects*			
Intercept	53.692	9.542	<0.001
DBH	0.323	0.089	<0.001
CIS	−104.003	20.081	<0.001
CIC	−44.234	15.984	0.006
CIS * CIC	97.586	27.059	<0.001
*Random effects*			
*δ*	1.315		<0.001
*ρ*	17.559		<0.001
*c*_o_	0.650		<0.001
*σ*_(resid. error)_	0.084		

### Effects of size on tree radial growth

**Fig 4:**
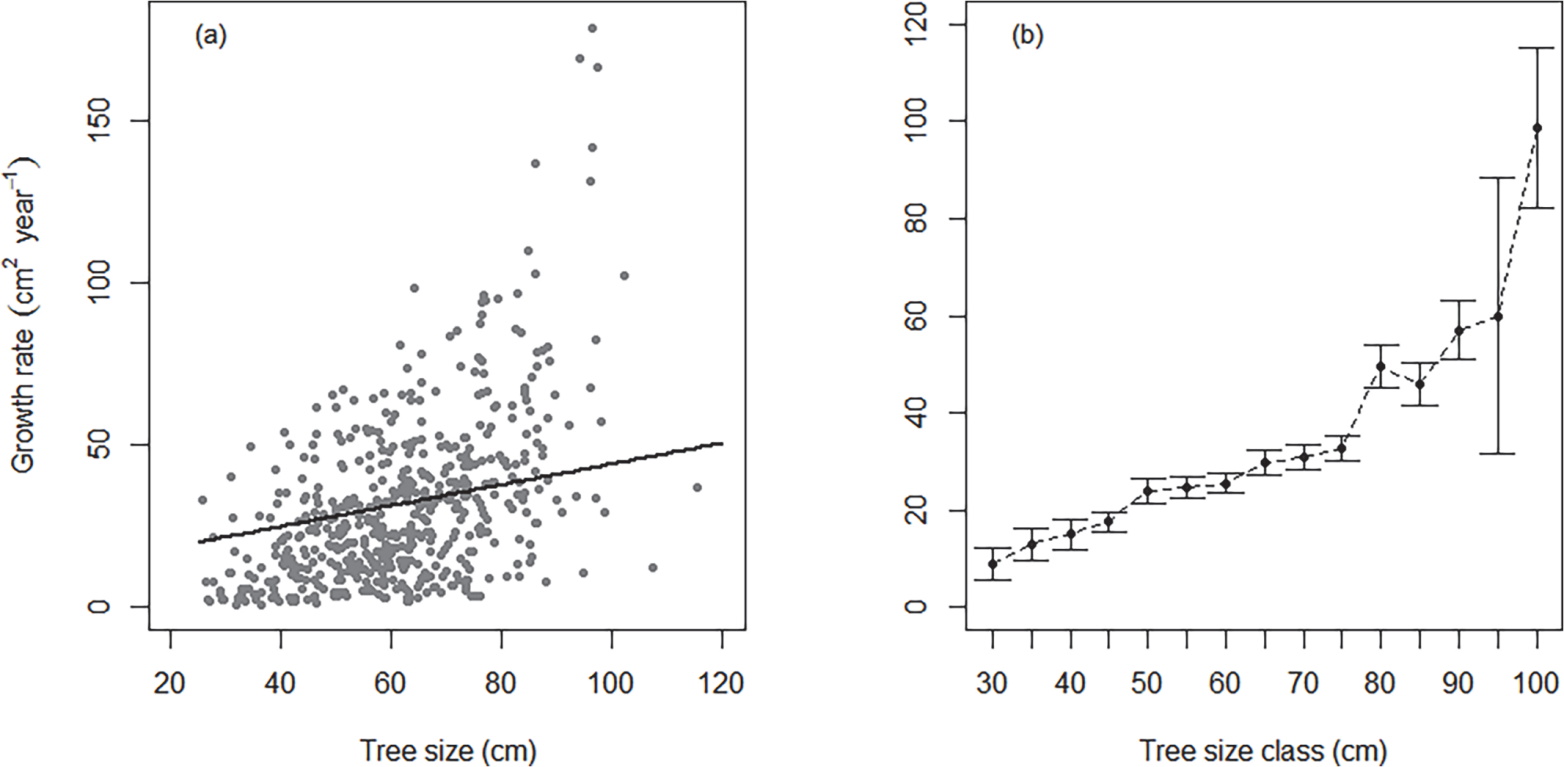
Radial growth rate as a function of tree size. (a) Predicted monotonic increase of basal area growth of dominant and co-dominant canopy beech (*Fagus sylvatica*) trees with trunk diameter (*β* = 0.323 ± 0.089; *P* < 0.001). Competition effects were kept fixed at their means (see Table 1). (b) Observed growth rates (mean ± SE) against tree size classes. The x-axis represents the upper boundaries of the tree size (DBH)-class.

Mean annual growth rates of beech increased continuously with DBH (Fig. 4A). For instance, the predicted growth of a large-sized tree with a DBH of 100 cm was 58% higher compared to a tree of 50 cm. Although growth pattern largely varied among individual trees of the same size (Fig. 4A), a distinct increase in average growth was obvious for trees > 75 cm (Fig. 4B). Mean annual growth was 32.6 cm^2^ year^−1^ in the 70–75 cm DBH range, 49.6 cm^2^ year^−1^ in the 75–80 cm DBH range and 98.8 cm^2^ year^−1^ in the 95–100 cm DBH range.

### Effects of above- and belowground competition on tree radial growth

**Fig 5:**
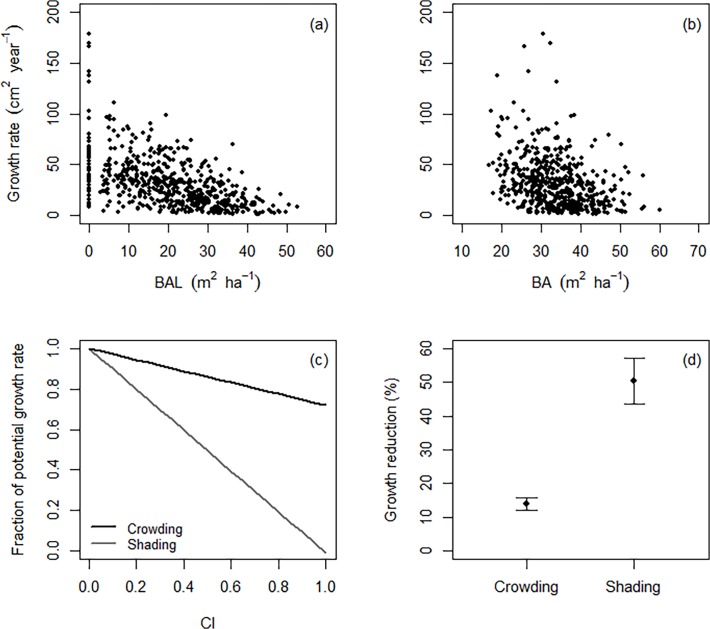
Effects of shading and crowding on radial growth. Growth rates of canopy dominants (*Fagus sylvatica*) in response to the cumulative basal area of (a) trees larger than the target tree (BAL; shading) and (b) all neighbours (BA; crowding) within a 20 m radius around a target tree. (c) Changes in competition response of canopy dominants with various levels of local neighbourhood competition. The response curve represents the predicted proportional decline in basal area growth as a function of shading and crowding effects, respectively. Competition effects are calculated for an overstorey beech tree of mean size and mean crowding or shading levels, while varying CI (see Table 1). (d) Relative growth reduction (mean ± SE) due to competition effects. Mean values were derived from the competition response curve in panel (c).

**Fig 6:**
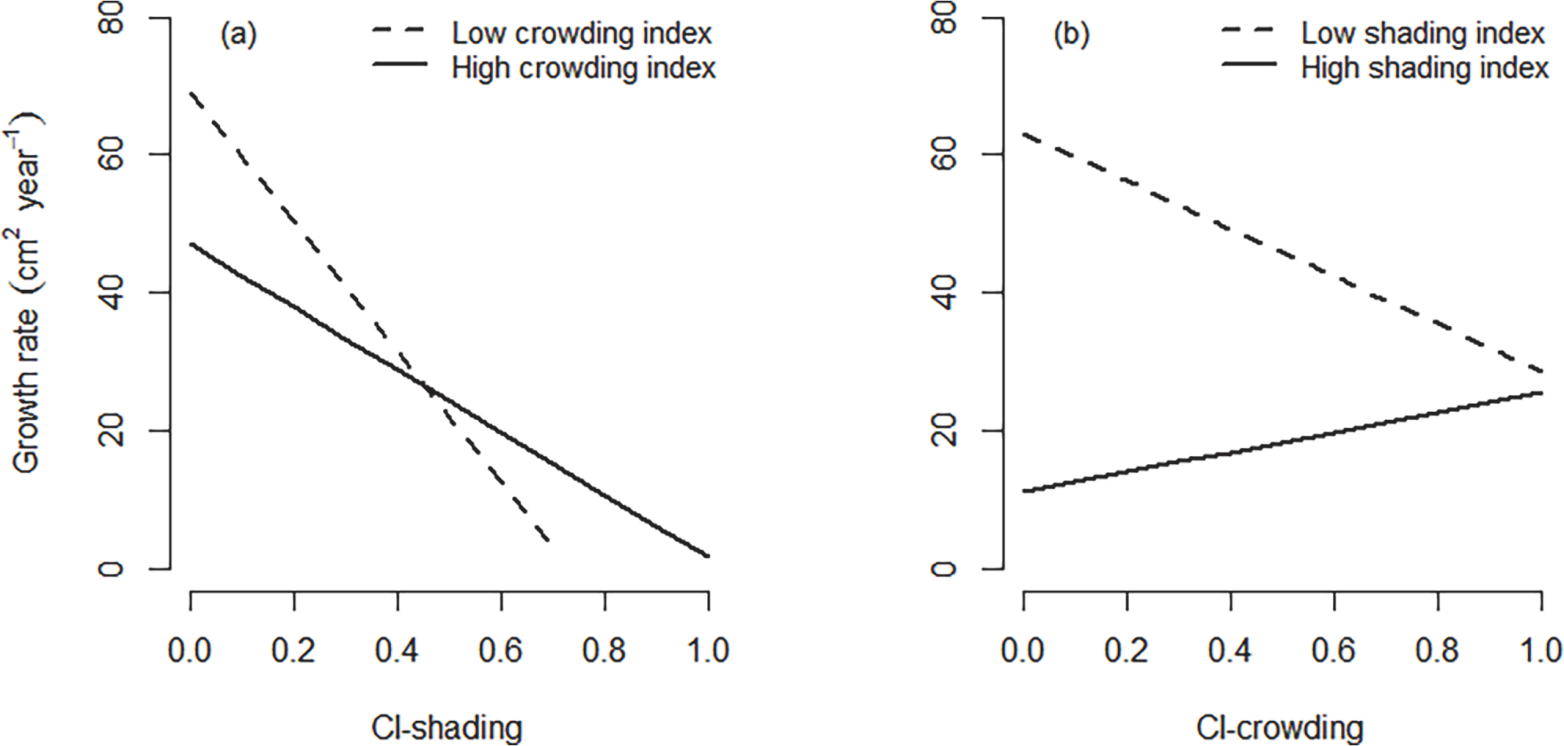
Variation in the effects of shading (a) and crowding (b) on radial growth rate at high and low level of competitive stress. The regression lines represent the estimated basal area growth for beech (*Fagus sylvatica*) of mean size growing in the overstorey (see Table 1) as predicted by the GLS-model.

Overall, radial growth decreased with increasing competition, although growth rates were immensely variable among trees experiencing the same level of competitive stress (Figs. 5A and 5B). Beech trees were less sensitive to changes in crowding conditions compared to variation in shading (Fig. 5C). Mean growth reduction due to local shading effects was 3.5-times higher than effects of crowding by neighbouring trees (*F*: 26.39, *P* < 0.001; Fig. 5D). However, the sensitivity to shading and crowding varied with the level of competitive stress. Changes in radial growth with increasing shading were less obvious at a high level of crowding (Fig. 6A). There was evidence of a shift to belowground facilitation for trees experiencing a high level of shading, where growth rates increased with increasing crowding (Fig. 6B).

**Fig 7:**
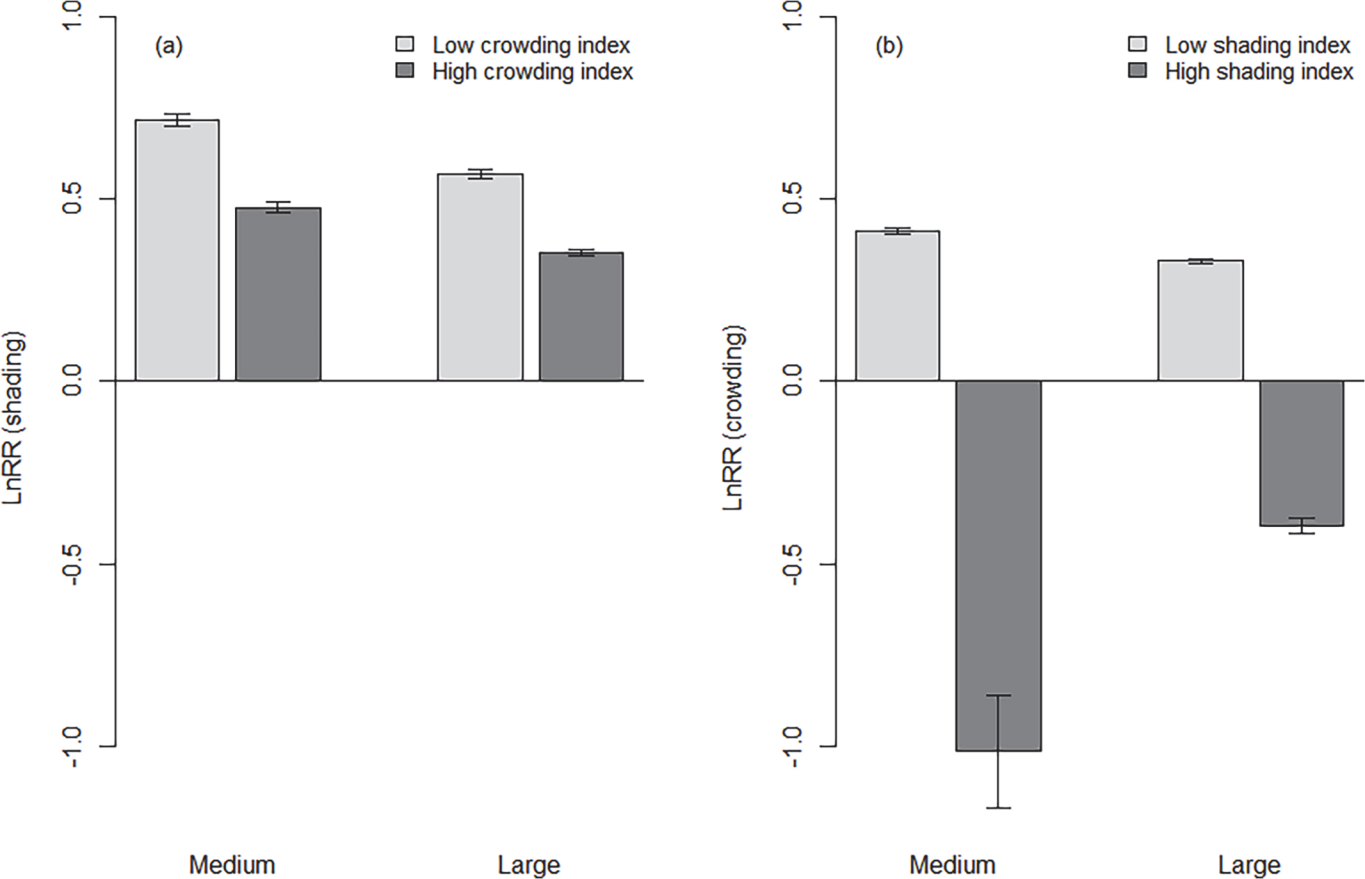
Variation in the intensity of neighbourhood interactions (LnRR) with tree size. (a) aboveground effects (shading), (b) belowground effects (crowding). Positive LnRR-values indicate competitive interactions, while negative values indicate facilitative interactions for medium-sized (DBH 30–60 cm) and large-sized (DBH 61–100 cm) dominant and co-dominant canopy beech (*Fagus sylvatica*) trees at low and high levels of competitive stress. Error bars represent 95% confidence intervals. Non-overlapping confidence intervals denote significant differences (*P* < 0.001) between stress levels.

We found antagonistic interactions between shading and crowding effects in which increasing competition for belowground resources was associated with decreased aboveground competition and vice versa (the light grey columns are always larger than the dark grey columns in Fig. 7). For example, LnRR (shading) was 34% (medium-sized trees) to 38% (large-sized trees) lower at high compared to low levels of crowding (both comparisons *P* < 0.001; Fig. 7A). Furthermore, at high levels of shading, radial growth was actually facilitated by a high density (crowding) of neighbouring trees (i.e., LnRR crowding showed negative values; both comparisons *P* < 0.001; Fig. 7B). There was also size-dependency in the magnitude of tree–tree interactions. Neighbourhood effects (LnRR shading and LnRR crowding) on target tree growth declined with tree size and tree size-related changes were most pronounced for crowding effects of trees experiencing a high level of shading (Fig. 7B). The decline in mean shading intensity with tree size was higher at a high (26%) compared to a low (21%) level of crowding (Fig. 7A).

## Discussion

We evaluated the effects of tree size and above- and belowground competition on individual radial tree growth of dominant and co-dominant beech canopy trees in an unmanaged old-growth forest. Our results provide evidence that growth rates generally decreased with increasing competition, but each mode of competition mitigates the effect of the other. In this context, we found that shading and crowding become less important with increasing tree size indicating size-asymmetry in both above- and belowground neighbourhood interactions. Here, we discuss the ecological significance of the observed growth strategies and their implications for forest community dynamics.

### Competitive interactions are less prevalent in the presence of large trees

Our results indicate that not only environmental gradients, but also the mode of competition (above- vs. belowground) and their interacting effects are important factors that determine the intensity and the outcome of inter–tree competition. Growth reduction due to shading was significantly lower for trees experiencing a high level of crowding. In contrast, beech trees were able to grow faster in neighbourhoods with high abundance of larger neighbours (i.e., high level of shading) due to facilitative effects of belowground interactions. These characteristics might partly result from the spatial arrangement of large and vigorous trees and their proximity to conspecific smaller neighbours, respectively. The regular-random spatial distribution of large-sized (> 60 cm in DBH) trees in our study and their high density strongly suggest that those individuals have a disproportional impact on the local shading and crowding conditions for a focal tree (see Figs. 2 and 3A). Natural late successional forests are associated with a wide range of tree sizes at a small spatial scale [42], thus high shading or crowding intensities (high values of BAL and BA) of structurally diverse stands depend primarily on the presence of large-diameter trees and not on a high abundance of smaller stems. This indicates that the importance of competition effects may vary with forest structure and larger trees may benefit or stimulate smaller neighbours. For example, competition intensity was found to be strongly related to the stand-level tree spatial pattern in an old growth boreal forest, where tree clustering locally intensified competition [16]. Moreover, old and large trees can operate as strong organizers of spatially-structured tree recruitment through competitive interactions [43] or facilitate regeneration establishment by integrating seedlings in existing mycorrhizal networks [44].

There are two plausible explanations for the observed higher radial increment of beech trees in more dense above- and belowground neighbourhoods as compared to BAI rates at low shading or crowding intensities. First, lower competition intensities for light at high levels of crowding likely arise as a result of a higher habitat heterogeneity induced by morphological adjustments and a long continuity in tree-tree interactions [30]. Morphological plasticity enables trees to reduce competitive pressure from neighbours [45,46,47,48], and thus improve their light absorption [12]. In the Serrahn forest, Schröter et al. [49] found that this adaptation mechanism also holds for old beech trees, which in turn would allow for shifts in carbon allocation pattern (i.e. allocation to the trunk instead of an allocation to branches) even at late-successional stages. Such shifts were observed in long-term unmanaged beech forests where crown efficiency (defined as growth per unit crown area) of *F*. *sylvatica* increased with increasing length of non-forestry use and stand density, particularly in the presence of allospecific neighbours [50]. We therefore assume that the lower importance of aboveground competition with increasing belowground competition is probably linked to an optimal light resource partitioning as a result of a higher structural complexity in crown sizes and shapes. Similar patterns were observed for adult trees in mixed-species forests [48]. Thus, optimal partitioning theory may explain the interacting effects between shading and crowding where high crowding intensities mitigate response to light limitations. This might hold for both medium- and large-sized trees, since we observed a size-independent plasticity of canopy dominants (*F*. *sylvatica*), as indicated by the similar decline in net shading effects with increasing belowground competition (see Fig. 7). However, the stimulating role of large-sized trees might not be evident in stands with a low growing stock and high anthropogenic disturbance intensity and frequency because morphological adjustments are minor as the system homogeneity is increased. The second possible explanation for large trees facilitating smaller trees is them being the primary contributors of a common mycorrhizal network or of an improved access to soil resources (e.g. by accelerating rates of nutrient cycling) [30,44,51]. There is evidence that those networks are involved in belowground transfers of carbon, nutrients (e.g. nitrogen, phosphorous) and water between ectomycorrhizal tree species (e.g. *F*. *sylvatica*), and thus can alter net competition effects [52,53,54]. However, the actual magnitude of such interplant transfers through directly connected fungal hyphae does not seem to be well understood [54]. We found that tree size-related changes in the intensity of neighbourhood interactions were context-specific (mode of competition) and varied when the interactions were dominated by above- or belowground processes (high or low level of the other mode). In our study belowground facilitation was caused by a high level of shading. Specifically, canopy dominants that were smaller in stature (DBH 30–60 cm; Fig. 7B) tended to have disproportionately higher facilitative effects belowground indicating that these trees mostly benefit from a spatial aggregation of larger trees in their local neighbourhood. Thus, in agreement with similar facilitative effects observed between seedlings and adult trees [44,55], we suggest that common mycorrhizal networks could be an important mechanism promoting growth rates of adult trees in conspecific neighbourhoods. Moreover, allocational plasticity enables canopy dominants to balance optimal production of root and canopy structures (e.g. optimising efficiencies of light interception and use vs. maximal depletion of shared soil resources to intensify competitive effects) [56,57,58]. As a result, adult trees can receive benefit from their neighbouring larger trees (high shading or crowding intensities) to achieve higher growth rates [59].

### Large trees play a key role for ecosystem functioning

The abundance of large-sized (> 60 cm in DBH) beech trees in our study cover a representative range of late-successional stages. However, we found no evidence for a size-related decline of growth rates during tree maturation (up to 100 cm in DBH). Instead, regardless of competitive stress, basal area growth of *F*. *sylvatica* continuously increased with size, which is in agreement with a continuous increase of BAI with age of mature beech trees (160–265 years) [60]. Enhanced CO_2_ levels in the recent decades might have contributed to increasing radial growth rates as trees age [61]. Similar results were found for long-living tree species (*Eucalyptus regnans* and *Sequoia sempervirens*) in old-growth forests located in Australia and North America, where aboveground wood production of un-suppressed individuals increased with size and age during the tree’s lifetime (largest and oldest trees: ‘*E*. *regnans’* 299 years / DBH 92 cm; ‘*S*. *sempervirens’* 1847 years / DBH 648 cm) [62]. Given the close correlation between basal area and diameter growth rates (*R* ^2^ = 0.89; S3 Fig) in our study, larger trees are assumed to be those which accumulate carbon in the trunk at even faster rates as they mature. Thus, suggesting that not only the amount of carbon, but also the rate of carbon sequestration is highest in old, large-sized trees [63]. However, the observed monotonic increase in growth rates with size might not necessarily be valid on the level of an individual tree, as individual-specific time series were not available [64]. In this context, we found a comparably low amount of variation in growth rates (33%) explained either directly or indirectly (via tree size) by competition. Similar results were observed for temperate tree species in a mixed-species primeval *Abieto-Fagetum* forest [65] and tropical tree species in an unmanaged old-growth forest [66]. This suggests that competition effects on tree radial growth are considerably less important in tree communities with a long continuity of population dynamics compared to frequently anthropogenic disturbed stands. Consequently, our results strongly highlight the importance of the abundance and spatial distribution of large-diameter trees in near-natural managed forests for the maintenance of ecosystem functioning.

## Conclusions

Interactions between neighbouring trees in long-term unmanaged communities may be more complex than commonly assumed, even at the intraspecific level. Recent studies have found evidence of such patterns in old-growth conifer forests [16,67]. Nonrandom demographic (density-dependent mortality and aggregated tree recruitment) processes can maintain tree patterns in a dynamic equilibrium [67], demonstrating that competitive interactions continue to affect forest structure and community processes over centuries [16,67]. Our research also has demonstrated that spatial aggregation of large-sized individuals could benefit growth of smaller conspecifics. It can therefore be considered that species competitive ability and neighbourhood competition intensity further depend on spatial aggregation patterns [16,68]. Thus, other factors such as forest structure or continuity of species interactions play a key role in regulating tree growth pattern and community dynamics in (near-) natural forest ecosystems.

Large-diameter and old trees are crucial components for maintaining biomass accumulation, carbon sequestration [62,63], structural heterogeneity [43], forest biodiversity [69] and forest integrity [70]. Our results additionally suggest that large-diameter trees have an important functional role for regulating biotic filtering processes. Moreover, the largest trees in our study were associated with the highest absolute radial growth rates, which might be a crucial mechanism for the maintenance of wood accumulation during stand development of old-growth forests [62]. This in turn emphasizes the need to reconsider the importance of large-diameter trees in (near-)natural forests to understand more fully interactions among conspecifics and allospecific neighbours, and thus forest community dynamics.

## Supporting Information

S1 Fig Residual plot of the best-fitting generalised least squares (GLS) regression model. (PDF)

S2 Fig Semivariogramm of the standardised residuals obtained by the best-fitting GLS model. (PDF)

S3 Fig Relationship between basal area and diameter growth rate of target trees. (PDF)

S1 Table Tree and stand characteristics of the 8 ha study plot of 2002 and 2009. (PDF)

S2 Table Observed growth rates and tree attributes of the modeling data set. (PDF)

## References

[1] Canham CD, Papaik MJ, Uriarte M, McWilliams WH, Jenkins JC, Twery MJ. Neighborhood analyses of canopy tree competition along environmental gradients in New England forests. Ecol Appl. 2006; 16: 540–554. 16711043 10.1890/1051-0761(2006)016[0540:naoctc]2.0.co;2

[2] Baribault TW, Kobe RK. Neighbour interactions strengthen with increased soil resources in a northern hardwood forest. J Ecol. 2011; 99: 1358–1372.

[3] Kunstler G, Albert CH, Courbaud B, Lavergne S, Thuiller W, Vieilledent G, et al Effects of competition on tree radial-growth vary in importance but not in intensity along climatic gradients. J Ecol. 2011 99: 300–312.

[4] Fichtner A, Sturm K, Rickert C, Härdtle W, Schrautzer J. Competition response of European beech *Fagus sylvatica* L. varies with tree size and abiotic stress: minimizing anthropogenic disturbances in forests. J Appl Ecol. 2012; 49: 1306–1315.

[5] Coates KD, Lilles EB, Astrup R. Competitive interactions across a soil fertility gradient in a multispecies forest. J Ecol. 2013; 101: 806–818.

[6] Lebourgeois F, Gomez M, Pinto P, Mérian P. Mixed stands reduce *Abies alba* tree-ring sensitivity to summer drought in the Vosges mountains, western Europe. For Ecol Manage. 2013; 303: 61–71.

[7] Forrester DI. The spatial and temporal dynamics of species interactions in mixed-species forests: from pattern to process. For Ecol Manage. 2014; 312: 282–292.

[8] Forrester DI, Vanclay JK, Forrester RI. The balance between facilitation and competition in mixtures of *Eucalyptus* and *Acacia* changes as stands develop. Oecologia. 2011; 166: 265–272. 10.1007/s00442-011-1937-9 21344256

[9] del Río M, Schütze G, Pretzsch H. Temporal variation of competition and facilitation in mixed species forests in Central Europe. Plant Biology. 2013; 10.1111/plb.12029 23581485

[10] García-Cervigón AI, Gazol A, Sanz V, Camarero JJ, Olano JM. The shifting nature of plant–plant interactions. Perspect Plant Ecol Evol Syst. 2013; 15: 226–236.

[11] He Q, Bertness MD, Altieri AH. Global shifts towards positive species interactions with increasing environmental stress. Ecol Lett. 2013; 16: 695–706. 10.1111/ele.12080 23363430

[12] Forrester DI, Albrecht AT. Light absorption and light-use efficiency in mixtures of *Abies alba* and *Picea abies* along a productivity gradient. For Ecol Manage. 2014; 328: 94–102.

[13] Mölder I, Leuschner C. European beech grows better and is less drought sensitive in mixed than in pure stands: tree neighbourhood effects on radial increment. Trees. 2014; 28: 777–792.

[14] Canham CD, LePage PT, Coates KD. A neighborhood analysis of canopy tree competition: effects of shading versus crowding. Can J For Res. 2004; 34: 778–787.

[15] Coates KD, Canham CD, Le Page PT. Above-versus below-ground competitive effects and responses of a guild of temperate tree species. J Ecol. 2009; 97: 118–130.

[16] Fraver S, D’Amato AW, Bradford JB, Jonsson BG, Jönsson M, Esseen PA. Tree growth and competition in an old-growth Picea abies forest of boreal Sweden: influence of tree spatial patterning. J Veg Sci. 2014; 25: 374–385.

[17] Schwinning S, Weiner J. Mechanisms determining the degree of size asymmetry in competition among plants. Oecologia. 1998; 113: 447–455. 28308024 10.1007/s004420050397

[18] Weiner J. Asymmetric competition in plant populations. Trends Ecol Evol. 1990; 5: 360–364. 10.1016/0169-5347(90)90095-U 21232393

[19] Larocque GR, Luckai N, Adhikary SN, Groot A, Bell FW, Sharma M. Competition theory—science and application in mixed forest stands: review of experimental and modelling methods and suggestions for future research. Environ Rev. 2013; 21: 71–84.

[20] Weiner J, Stoll P, Muller-Landau H, Jasentuliyana A. The effects of density, spatial pattern, and competitive symmetry on size variation in simulated plant populations. Am Nat. 2001; 158: 438–450. 10.1086/321988 18707338

[21] Cahill JF, Casper BB. Investigating the relationship between neighbour root biomass and belowground competition: field evidence for symmetric competition belowground. Oikos.2000; 90: 311–320.

[22] Xu H, Li Y. Water-use strategy of three central Asian desert shrubs and their responses to rain pulse events. Plant Soil. 2006; 285: 5–17.

[23] Coomes DA, Allen RB. Effects of size, competition and altitude on tree growth. J Ecol 95 2007;: 1084–1097.

[24] Rewald B, Leuschner C. Belowground competition in a broad-leaved temperate mixed forest: pattern analysis and experiments in four-species stand. Eur J For Res. 2009; 128: 387–398.

[25] Lei P, Scherer-Lorenzen M, Bauhus J. Belowground facilitation and competition in young tree species mixtures. For Ecol Manage. 2012; 265: 191–200.

[26] Beyer F, Hertel D, Jung K, Fender A-C, Leuschner C. Competition effects on fine root survival of *Fagus sylvatica* and *Fraxinus excelsior* . For Ecol Manage. 2013; 302: 14–22.

[27] Kiær LP, Weisbach AN, Weiner J. Root and shoot competition: a meta-analysis. J Ecol. 2013; 101: 1298–1321.

[28] Bertness MD, Callaway R. Positive interactions in communities. Trends Ecol Evol. 1994; 9: 191–193. 10.1016/0169-5347(94)90088-4 21236818

[29] Brooker RW, Maestre FT, Callaway RM, Lortie CL, Cavieres LA, Kunstler G, et al Facilitation in plant communities: the past the present, and the future. J Ecol. 2008; 96: 18–34.

[30] McIntire EJB, Fajardo A. Facilitation as a ubiquitous driver for biodiversity. New Phytol. 2014; 201: 403–416. 24102266 10.1111/nph.12478

[31] Fajardo A, McIntire EJB. Under strong niche overlap conspecifics do not compete but help each other to survive: facilitation at the intraspecific level. J Ecol. 2011; 99: 642–650.

[32] von Oheimb G, Westphal C, Tempel H, Härdtle W. Structural pattern of a near-natural beech forest (*Fagus sylvatica*) (Serrahn, North-east Germany). For Ecol Manage. 2005; 212: 253–263.

[33] Dieckmann O, Großmann M. Weltnaturerbe “Alte Buchenwälder Deutschlands” Teilgebiet Serrahn. AFZ Der Wald Sonderheft. 2012; 12–15.

[34] Westphal C, Tremer N, von Oheimb G, Hansen J, von Gadow K, Härdtle W. Is the reverse J-shaped diameter distribution universally applicable in European virgin beech forests? For Ecol Manage. 2006; 223, 75–83.

[35] Oliver CD, Larson BC. Forest stand dynamics New York: Wiley & Sons 1996; 520 p.

[36] Pinheiro JC, Bates DM. Mixed-effects models in S and S-Plus New York: Springer 2004; 528 p.

[37] Fortin M, Daigle G, Ung CH, Begin J, Archambault L. A variance-covariance structure to take into account repeated measurements and heteroscedasticity in growth modelling. Eur J For Res. 2007; 126: 573–585.

[38] Zuur AF, Ieno EN, Walker N, Saveliev P, Smith GM. Mixed effects models and extensions in ecology with R New York: Springer 2009; 574 p.

[39] Burnham KP, Anderson DR. Model selection and multimodel inference: a practical information-theoretical approach New York: Springer 2002; 488 p.

[40] R Development Core Team. R: A language and environment for statistical computing R Foundation for Statistical Computing, Vienna, Austria 2012; Available: http://www.R-project.org/.

[41] Hedges LV, Gurevitch J, Curtis PS. The meta-analysis of response ratios in experimental ecology. Ecology. 1999; 80: 1150–1156.

[42] Piovesan G, Di Filippo A, Alessandrini A, Biondi F, Schirone B. Structure, dynamics and dendroecology of an old-growth *Fagus* forest in the Apennines. J Veg Sci. 2005; 16: 13–28.

[43] Lutz JA, Larson AJ, Freund JA, Swanson ME, Bible KJ. The importance of large-diameter trees to forest structural heterogeneity. PLoS ONE. 2013; 8: e82784 10.1371/journal.pone.0082784 24376579 PMC3869720

[44] Simard SW. The foundational role of mycorrhizal networks in self-organization of interior Douglas-fir forests. For Ecol Manage. 2009; 258: 95–107.

[45] Lang AC, Härdtle W, Bruelheide H, Geißler C, Nadrowski K, Schuldt A, Yu M, von Oheimb G. Tree morphology responds to neighborhood competition and slope in species-rich forests of subtropical China. For Ecol Manage. 2010; 260: 1708–1715.

[46] Seidel D, Leuschner C, Müller A, Krause B. Crown plasticity in mixed forests—quantifying asymmetry as a measure of competition using terrestrial laser scanning. For Ecol Manage. 2011; 261: 2123–2132.

[47] Dieler J, Pretzsch H. Morphological plasticity of European beech (*Fagus sylvatica* L.) in pure and mixed-species stands. For Ecol Manage. 2013; 295: 97–108.

[48] Pretzsch H. Canopy space filling and tree crown morphology in mixed-species stands compared with monocultures. For Ecol Manage. 2014; 327: 251–264.

[49] Schröter M, Härdtle W, von Oheimb G. Crown plasticity and neighborhood interactions of European beech (Fagus sylvatica L.) in an old-growth forest. Eur J For Res. 2012; 131: 787–798.

[50] Fichtner A, Sturm K, Rickert C, von Oheimb G, Härdtle W. Crown size-growth relationships of European beech (*Fagus sylvatica* L.) are driven by the interplay of disturbance intensity and inter-specific competition. For Ecol Manage. 2013; 302: 178–184.

[51] Rothe A, Binkley D. Nutritional interactions in mixed species forests: a synthesis. Can J For Res. 2001; 31: 1855–1870.

[52] Simard SW, Perry DA, Jones MD, Myrold DD, Durall DM, Molina R. Net transfer of carbon between ectomycorrhizal tree species in the field. Nature. 1997; 388: 579–582.

[53] Lukac M, Godbold DL. Soil ecology in northern forests United Kingdom: Cambridge University Press 2011; 256 p.

[54] Simard SW, Beiler KJ, Bingham MA, Deslippe JR, Philip LJ, Teste FP. Mycorrhizal networks: mechanisms, ecology and modeling. Fungal Biol Rev. 2012; 26: 39–60.

[55] Teste FP, Simard SW, Durall DM. Role of mycorrhizal networks and tree proximity in ectomycorrhizal colonization of planted seedlings. Fungal Ecol. 2009; 2: 21–30.

[56] Weiner J. Allocation, plasticity and allometry in plants. PPEES. 2004; 6: 207–215.

[57] Craine JM, Fargione J, Sugita S. Supply pre-emption, not concentration reduction, is the mechanism of competition for nutrients. New Phytol. 2005; 166: 933–940. 15869653 10.1111/j.1469-8137.2005.01386.x

[58] Onoda Y, Saluñga JB, Akutsu K, Aiba S, Yahara T, Anten NPR. Trade-off between light interception efficiency and light use efficiency: implications for species coexistence in one-sided light competition. J Ecol. 2014; 102: 167–175.

[59] Hertel D, Strecker T, Müller-Haubold H, Leuschner C. Fine root biomass and dynamics in beech forests across a precipitation gradient—is optimal resource partitioning theory applicable to water-limited mature trees? J Ecol. 2013; 101: 1183–1200.

[60] Härdtle W, Niemeyer T, Assmann T, Baiboks S, Fichtner A, Friedrich U, et al Long-term trends in tree-ring width and isotope signatures (*δ* ^13^C, *δ* ^15^N) of *Fagus sylvatica* L. on soils with contrasting water supply. Ecosystems. 2013; 16: 1413–1428. 10.1111/ele.12145 23837659

[61] Peñuelas J, Hunt JM, Ogaya R, Jump AS. Twentieth century changes of tree-ring *δ* ^13^C at the southern range-edge of *Fagus sylvatica*: increasing water-use efficiency does not avoid the growth decline induced by warming at low altitudes. Glob Change Biol. 2008; 14:1076–88.

[62] Sillett SC, Van Pelt R, Koch GW, Ambrose AR, Carroll AL, Antoine ME, et al Increasing wood production through old age in tall trees. For Ecol Manage. 2010; 259: 976–994.

[63] Stephenson NL, Das AJ, Condit R, Russo SE, Baker PJ, Beckman NG, et al Rate of tree carbon accumulation increases continuously with tree size. Nature. 2014; 507: 90–93. 10.1038/nature12914 24429523

[64] Eastaugh CS, Thurnher C, Hasenauer H, Vanclay JK. Stephenson et al.’s ecological fallacy. 2014; arxiv.org/abs/1403.0630 [q-bio.QM].

[65] Bošela M, Petráš R, Šeben V, Mecko J, Marušák R. Evaluating competitive interactions between trees in mixed forests in the Western Carpathians: Comparison between long-term experiments and SIBYLA simulations. For Ecol Manage. 2013; 15: 577–588.

[66] Rüger N, Berger U, Hubbell SP, Vieilledent G, Condit R. Growth strategies of tropical tree species: disentangling light and size effects. PLoS ONE. 2011; 6: e25330 10.1371/journal.pone.0025330 21966498 PMC3178650

[67] Lutz JA, Larson AJ, Furniss TJ, Donato DC, Freund JA, Swanson ME, et al Spatially nonrandom tree mortality and ingrowth maintain equilibrium pattern in an old-growth *Pseudotsuga*–*Tsuga* forest. Ecology. 2014; 95: 2047–2054. 25230456 10.1890/14-0157.1

[68] Semchenko M Abakumova M, Lepik A, Zobel K. Plants are least suppressed by their frequent neighbours: the relationship between competitive ability and spatial aggregation patterns. J Ecol. 2013; 101: 1313–1321.

[69] Brunet J, Fritz Ö, Richnau G. Biodiversity in European beech forests—a review with recommendations for sustainable forest management. Ecol Bull. 2010; 53: 77–94.

[70] Lindenmayer DB, Laurance WF, Franklin JF. Global decline in large old trees. Science. 2012; 338: 1305–1306. 10.1126/science.1231070 23224548

