# Supplementary figures and images for: Facilitative-Competitive Interactions in an Old-Growth Forest: The Importance of Large-Diameter Trees as Benefactors and Stimulators for Forest Community Assembly
Source: PLoS One. 2015 Mar 24;10(3):e0120335. doi: 10.1371/journal.pone.0120335 (PMC4372556; doi:10.1371/journal.pone.0120335)

**S1 Fig. Residual plot of the best-fitting generalised least squares (GLS) regression model.**

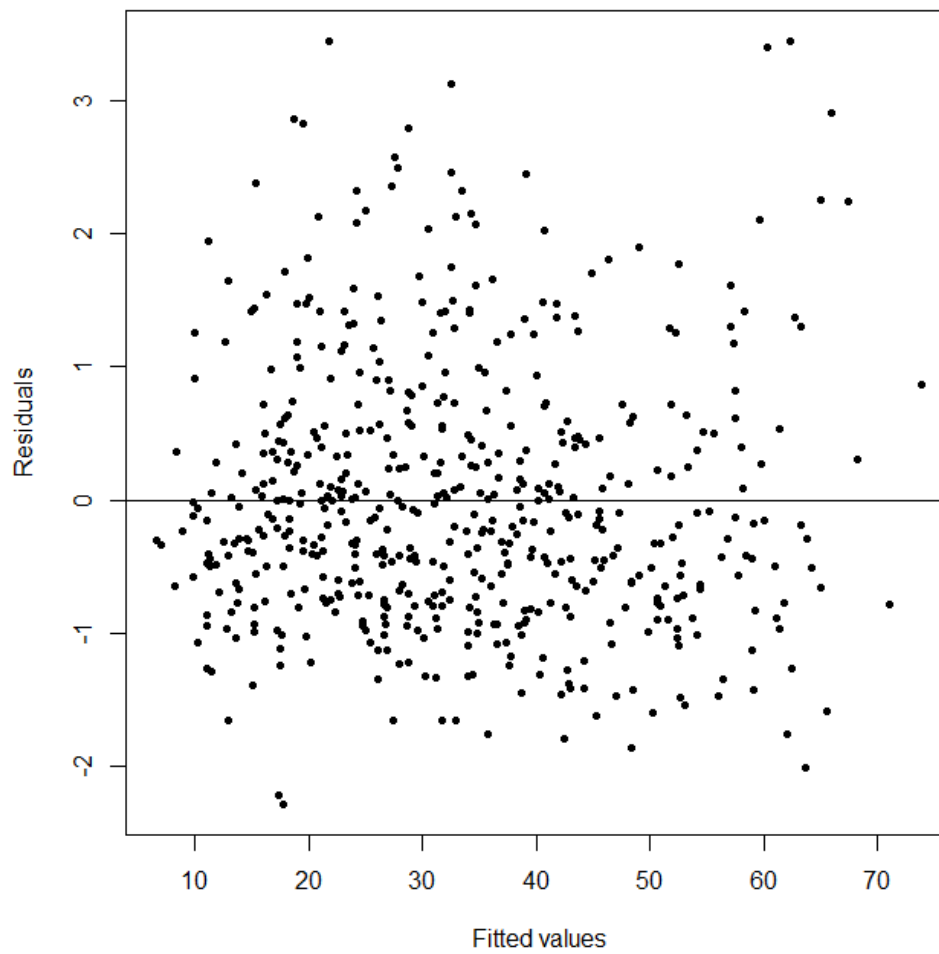

Supplement: S1 Fig — (PDF) [file pone.0120335.s001.pdf]

**S2 Fig. Semivariogram of the standardised residuals obtained by the best-fitting GLS model.**

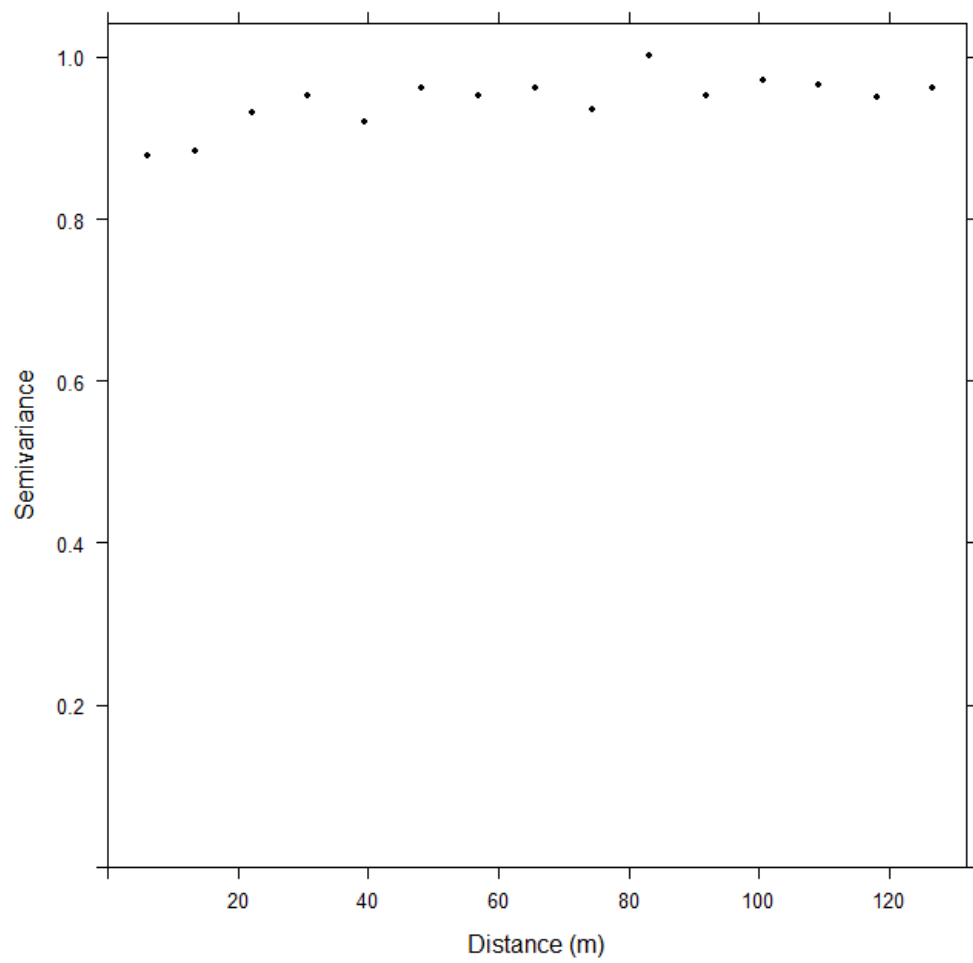

Supplement: S2 Fig — (PDF) [file pone.0120335.s002.pdf]

**S3 Fig. Relationship between basal area and diameter growth rate of target trees.**

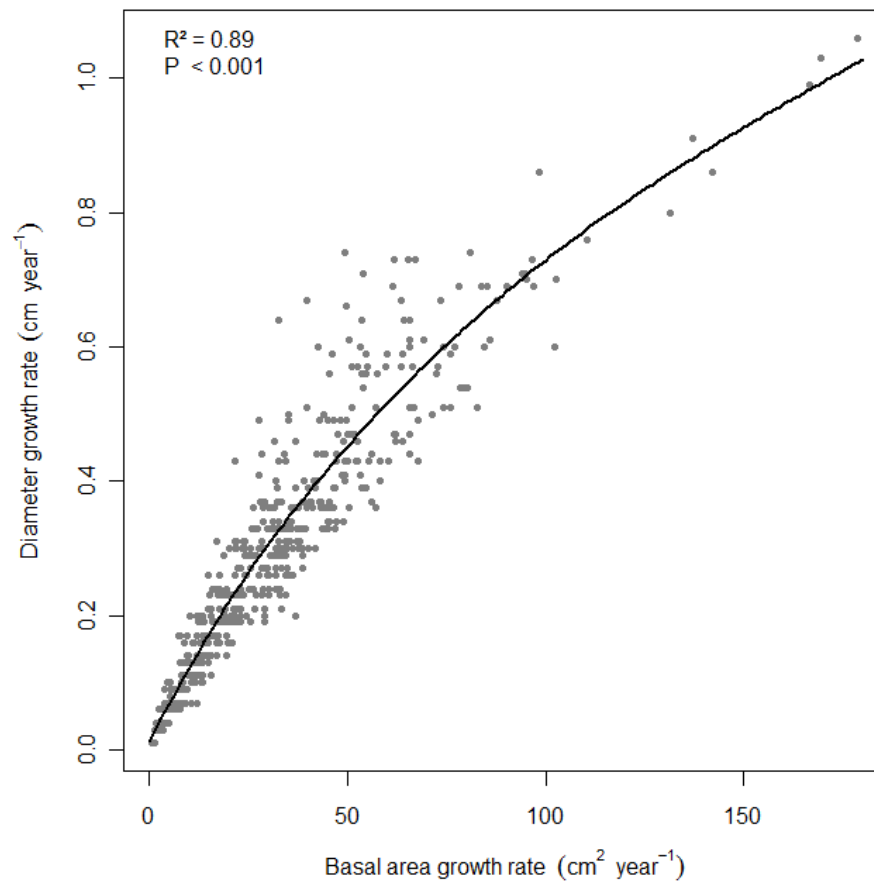

Supplement: S3 Fig — (PDF) [file pone.0120335.s003.pdf]
