# Supplementary material for: Facilitative-Competitive Interactions in an Old-Growth Forest: The Importance of Large-Diameter Trees as Benefactors and Stimulators for Forest Community Assembly
Source: PLoS One. 2015 Mar 24;10(3):e0120335. doi: 10.1371/journal.pone.0120335 (PMC4372556; doi:10.1371/journal.pone.0120335)
Supplement: S1 Table — (PDF) [file pone.0120335.s004.pdf]

S1 Table. Tree and stand characteristics of the 8 ha study plot of 2002 and 2009.

	Fagus sylvatica		*Pinus sylvestris*		*Quercus petraea*		Other species	
	2002	2009	2002	2009	2002	2009	2002	2009
Overstorey trees								
Stand density (N ha^{-1})	97.0	96.0	3.0	3.0	2.0	1.0	-	-
Stand basal area (m^2 ha^{-1})	30.0	31.6	0.9	0.8	0.6	0.4	-	-
Stand volume (m^3 ha^{-1})	572.1	613.2	12.7	13.5	14.5	9.0	-	-
Diameter at 1.30 (cm)	60.1	62.4	59.9	59.9	77.2	77.9	-	-
Understorey trees								
Stand density (N ha^{-1})	163	221	-	-	-	-	1.0	1.0
Stand basal area (m^2 ha^{-1})	1.2	2.0	-	-	-	-	0.01	0.01
Stand volume (m^3 ha^{-1})	5.7	10.6	-	-	-	-	0.03	0.05
Diameter at 1.30 (cm)	9.5	10.1	-	-	-	-	9.7	10.3
