# Supplementary material for: Facilitative-Competitive Interactions in an Old-Growth Forest: The Importance of Large-Diameter Trees as Benefactors and Stimulators for Forest Community Assembly
Source: PLoS One. 2015 Mar 24;10(3):e0120335. doi: 10.1371/journal.pone.0120335 (PMC4372556; doi:10.1371/journal.pone.0120335)
Supplement: S2 Table — (PDF) [file pone.0120335.s005.pdf]

S2 Table. Observed growth rates and tree attributes of the modeling data set.

Stratum	X-coordinates	Y-coordinates	DBH 2002 (cm)	DBH 2009 (cm)	BAI (cm^2 $year^{-1}$)	BA (r=10 m)	BA (r=15 m)	BA (r=20 m)	BAL (r=10 m)	BAL (r=15 m)	BAL (r=20 m)
Understorey	100.6378	353.4414	22.70	23.00	1.56	36.50	31.72	39.57	36.31	31.53	39.36
Understorey	77.4089	192.6660	21.90	25.20	19.88	3.90	32.32	30.96	2.84	30.05	28.66
Understorey	126.2830	351.1153	21.70	21.90	0.99	71.01	40.61	36.74	70.67	40.36	36.02
Understorey	39.2554	196.5310	21.20	23.30	11.47	30.06	30.02	31.23	25.19	25.96	27.19
Understorey	45.3582	204.1783	20.00	22.70	14.57	4.79	17.40	24.78	1.36	12.45	21.10
Understorey	57.7066	370.7643	19.80	19.90	0.45	49.41	33.36	21.89	49.41	32.91	20.97
Understorey	95.7972	84.2150	19.40	23.80	25.67	8.91	15.70	15.53	0.00	5.94	6.86
Understorey	85.4501	82.7327	19.40	22.30	15.46	23.32	25.81	23.80	13.36	17.81	17.03
Understorey	110.9975	290.8623	19.00	24.30	32.05	9.90	24.77	28.27	0.00	16.97	22.51
Understorey	175.5435	105.1715	19.00	24.20	31.27	26.22	43.90	27.76	24.02	41.26	24.85
Understorey	26.6823	218.7785	18.90	22.50	19.63	26.00	21.44	27.13	24.71	18.57	24.68
Understorey	115.0848	131.3976	18.70	20.20	7.05	35.10	31.63	29.65	30.62	27.98	26.84
Understorey	43.0870	191.1980	18.60	23.70	30.04	26.28	29.15	25.56	21.61	24.80	21.52
Understorey	96.7302	93.8437	18.50	20.10	7.50	12.06	20.37	17.45	3.76	11.99	10.54
Understorey	137.5000	344.8000	18.20	22.50	23.78	30.26	46.80	38.84	28.45	43.27	36.15
Understorey	117.5968	291.2030	18.20	22.50	23.78	18.69	13.28	25.11	9.37	6.65	19.78
Understorey	57.4017	189.6224	18.10	19.30	5.36	15.52	16.82	28.30	10.01	12.72	25.25
Understorey	86.5931	185.4384	18.00	19.30	5.82	15.56	27.71	33.10	13.76	25.61	31.43
Understorey	56.6990	200.1169	17.90	19.70	8.32	22.53	31.08	22.96	17.59	27.01	20.03
Understorey	145.7441	297.6806	17.70	21.00	16.77	54.39	33.07	29.40	43.41	24.25	22.79
Understorey	145.8773	334.1422	17.40	20.90	17.79	24.48	22.48	31.81	19.47	17.53	27.80
Understorey	34.1388	347.3001	17.30	22.80	31.53	20.10	24.98	37.01	16.08	22.03	34.35
Understorey	112.6972	148.7983	17.20	23.30	36.07	43.14	43.58	34.50	43.14	42.27	33.13
Understorey	61.3267	176.2905	17.20	18.50	5.59	66.10	39.69	41.09	65.10	38.02	39.65
Understorey	81.4348	92.8994	17.20	18.40	5.12	7.26	17.38	22.51	2.08	12.40	17.51
Understorey	49.7415	192.9503	17.20	18.30	4.65	6.58	23.32	24.05	3.31	19.60	21.55
Understorey	94.5064	95.8499	17.10	22.60	31.29	12.35	10.98	17.67	0.00	0.63	9.11
Understorey	78.0006	106.4348	17.00	19.80	13.32	30.94	26.20	22.03	28.35	22.10	17.13
Understorey	53.4836	197.6170	17.00	17.60	2.41	8.94	13.61	35.19	5.89	10.83	32.94
Understorey	100.1082	312.3995	16.90	19.10	9.97	24.43	25.08	31.16	23.23	23.69	29.34
Understorey	83.7912	91.5994	16.80	20.10	16.11	9.03	25.50	23.19	2.31	17.66	16.52
Understorey	91.1478	82.0074	16.50	20.70	21.49	23.12	15.57	23.57	16.02	7.69	16.50
Understorey	179.7533	300.6680	16.40	21.30	26.11	37.60	25.39	27.98	34.53	22.40	25.47
Understorey	120.9254	298.6809	16.40	18.00	6.75	20.98	17.85	25.19	15.86	12.66	20.64
Understorey	87.7234	93.8961	16.40	16.70	1.13	14.49	11.81	17.09	9.13	6.08	12.55
Understorey	93.3111	98.9049	16.20	19.60	16.25	14.61	10.15	22.10	3.36	2.57	15.89
Understorey	96.4527	91.4860	16.20	17.00	3.12	11.15	16.10	18.18	4.66	9.62	12.79
Understorey	22.5756	182.0550	15.90	19.70	18.42	37.65	22.97	31.25	32.97	18.80	27.54
Understorey	45.6351	196.8584	15.90	16.00	0.36	11.16	24.38	24.35	9.32	22.81	22.04
Understorey	139.2418	294.4861	15.80	21.00	27.54	43.49	30.07	24.29	38.25	23.48	17.29
Understorey	91.1705	75.9835	15.70	20.60	25.34	23.49	16.59	22.61	17.09	12.03	16.44
Understorey	84.9065	86.3961	15.70	17.00	5.15	22.93	26.98	25.57	17.53	22.03	20.68

Understorey	90.9500	105.9756	15.60	18.20	11.38	12.28	8.91	25.69	2.03	2.35	20.64
Understorey	144.7568	334.8071	15.60	15.70	0.35	24.86	22.68	27.19	20.56	19.22	24.15
Understorey	90.8859	97.3334	15.50	16.60	4.23	15.30	10.98	14.74	9.08	5.72	10.21
Understorey	86.6980	178.8009	15.40	17.20	7.31	31.45	40.52	33.07	31.00	38.99	31.88
Understorey	153.7907	325.2651	15.40	17.00	6.39	36.88	40.89	28.13	31.44	34.76	22.75
Understorey	32.9465	204.2252	15.40	16.70	5.06	40.48	30.25	28.81	37.47	27.26	26.32
Understorey	86.2217	73.3367	15.10	18.90	17.74	21.33	33.30	36.64	16.73	28.69	32.57
Understorey	88.2689	97.2698	15.00	20.70	30.12	14.31	10.93	13.55	1.28	1.12	6.06
Understorey	81.8781	22.6972	15.00	18.00	13.13	24.52	23.36	36.46	18.23	18.06	31.08
Understorey	35.1823	210.1823	15.00	17.10	8.55	5.10	30.43	23.77	0.00	27.49	21.46
Understorey	110.3169	81.3222	15.00	16.00	3.70	5.79	18.53	26.44	2.99	16.00	23.76
Understorey	148.3329	289.4101	14.90	17.40	10.46	23.63	26.90	23.19	18.21	20.62	17.57
Understorey	88.1725	102.1995	14.90	17.00	8.51	12.45	10.41	23.20	4.92	4.26	18.44
Understorey	75.0146	182.5196	14.90	16.20	4.92	42.96	42.98	33.61	41.57	42.05	32.37
Understorey	97.6118	108.4848	14.80	18.00	14.07	21.13	29.08	23.04	13.72	23.19	18.52
Understorey	149.2406	290.4273	14.80	17.70	12.46	23.52	27.51	23.41	17.42	20.27	17.38
Understorey	48.9860	194.4015	14.70	16.30	6.14	8.92	23.32	24.35	6.27	20.62	22.30
Understorey	86.2696	108.9642	14.70	15.80	4.04	7.70	19.92	13.81	3.31	15.95	10.18
Understorey	112.3654	80.8980	14.60	16.90	9.32	25.52	22.82	25.24	22.08	20.33	22.80
Understorey	81.7854	196.0304	14.50	15.10	2.07	28.15	28.12	26.53	27.75	27.34	25.52
Understorey	117.7876	124.3761	14.40	18.60	19.51	35.90	26.86	33.01	32.28	22.78	29.66
Understorey	134.7388	115.8534	14.40	17.50	13.25	6.11	14.89	21.58	0.79	10.45	18.08
Understorey	119.3338	296.2469	14.30	15.50	4.34	14.18	17.85	20.99	9.14	13.02	16.28
Understorey	110.5527	297.0017	14.30	15.40	3.94	17.76	18.88	23.41	12.80	13.48	18.64
Understorey	97.7855	77.9493	14.30	15.10	2.78	8.12	14.47	20.64	3.55	10.67	16.92
Understorey	137.5777	308.8997	14.20	18.30	18.72	46.21	42.39	29.84	41.92	36.61	23.96
Understorey	63.0887	192.6666	14.20	16.50	9.11	9.82	24.15	30.20	7.89	21.62	27.90
Understorey	97.4010	102.8391	14.20	15.60	5.12	12.14	34.38	24.59	7.77	30.34	21.08
Understorey	47.9425	338.5975	14.00	15.70	6.31	21.77	45.39	30.65	18.78	43.28	28.42
Understorey	80.2426	91.1709	14.00	15.60	5.89	8.10	32.15	26.09	4.52	29.79	23.01
Understorey	114.3624	137.4616	13.90	18.50	21.47	51.04	38.91	34.90	49.81	37.32	32.99
Understorey	97.2030	114.9192	13.90	15.00	3.84	19.54	29.10	28.33	15.52	26.15	25.62
Understorey	91.5330	120.7689	13.90	14.40	1.64	10.78	27.37	31.69	8.55	25.34	29.66
Understorey	160.4217	313.7796	13.80	16.30	9.85	27.92	20.84	33.78	21.57	14.02	27.75
Understorey	30.7502	204.3331	13.80	14.80	3.43	56.04	30.03	28.59	54.20	27.82	26.79
Understorey	93.4452	81.4880	13.70	14.20	1.62	8.52	15.24	17.69	6.08	11.61	14.51
Understorey	157.9221	301.1134	13.60	16.60	12.18	23.37	35.56	31.56	16.32	28.14	25.43
Understorey	94.2338	100.9464	13.60	16.60	12.18	14.95	9.63	25.30	8.88	4.40	21.03
Understorey	151.5733	303.8942	13.50	16.60	12.63	53.87	39.02	34.86	44.51	30.62	28.63
Understorey	32.1710	347.3700	13.50	15.70	8.29	22.16	19.45	34.67	19.21	17.41	32.58
Understorey	133.2259	118.6987	13.40	17.80	19.75	6.89	8.29	25.50	0.00	4.03	21.48
Understorey	150.8409	344.3486	13.40	16.30	11.55	26.92	28.39	26.69	23.86	26.05	24.51
Understorey	128.5837	309.3358	13.40	16.00	10.09	25.33	26.59	30.34	20.76	23.42	26.64
Understorey	113.0181	297.3864	13.40	15.40	7.36	8.15	21.12	18.09	4.15	15.97	13.59
Understorey	158.7412	329.6478	13.40	14.90	5.27	47.42	32.97	29.80	43.93	29.46	25.95
Understorey	67.2553	189.0451	13.40	13.80	1.26	16.28	28.20	40.75	15.04	26.86	39.52
Understorey	97.8097	66.8762	13.30	15.60	8.64	15.03	20.15	19.54	9.99	16.51	16.27
Understorey	101.1029	21.4825	13.30	14.80	5.23	38.67	29.57	31.28	32.09	24.10	26.60
Understorey	127.9301	133.5097	13.20	17.00	16.12	4.62	24.11	23.04	0.00	20.67	19.95
Understorey	155.4565	326.2597	13.20	16.10	11.42	36.26	34.11	28.06	32.16	28.93	23.26
Understorey	142.8571	285.5139	13.20	15.70	9.51	8.41	20.64	27.03	3.35	16.40	22.60

Understorey	42.2547	204.1332	13.20	15.10	6.84	6.57	19.30	28.40	4.71	16.97	26.45
Understorey	122.3411	277.2720	13.20	15.10	6.84	31.54	26.37	22.05	26.06	21.61	17.97
Understorey	121.9775	306.5137	13.20	14.90	6.01	14.14	18.64	22.34	10.45	16.21	19.86
Understorey	139.4972	300.6489	13.20	14.00	2.59	47.49	33.96	32.37	44.11	30.98	29.12
Understorey	140.7174	115.1997	13.10	16.90	16.03	5.22	18.27	26.80	1.56	14.72	24.24
Understorey	57.9847	182.7107	13.10	13.40	0.91	24.07	29.56	35.43	22.62	28.61	34.47
Understorey	76.7191	22.2496	13.00	17.80	21.76	44.29	37.61	35.54	41.45	33.60	30.88
Understorey	178.7622	97.8366	13.00	17.20	18.19	18.17	25.72	21.62	13.78	22.47	19.07
Understorey	143.3376	282.7468	13.00	16.80	15.95	6.77	15.42	25.21	1.54	10.64	21.02
Understorey	24.8698	175.8650	13.00	16.20	12.78	37.60	24.60	29.96	34.42	20.58	26.86
Understorey	59.9366	186.6342	13.00	15.20	8.05	13.60	23.49	38.99	11.63	21.95	37.47
Understorey	42.7605	23.2855	13.00	15.10	7.61	52.51	38.40	38.22	47.55	33.35	34.16
Understorey	91.7768	114.4491	13.00	14.60	5.53	6.97	27.94	30.21	2.82	24.95	27.96
Understorey	39.2521	23.5621	12.90	16.00	12.21	40.24	33.28	35.88	34.20	28.21	31.69
Understorey	153.4114	302.6177	12.80	16.10	13.14	53.55	29.21	34.28	45.89	21.38	28.55
Understorey	124.0450	293.8995	12.80	15.30	9.28	10.60	20.73	15.03	8.27	17.33	10.34
Understorey	63.0491	206.2760	12.80	13.50	2.18	60.70	38.25	29.96	60.32	37.27	29.32
Understorey	108.9056	82.7415	12.70	14.50	6.22	18.12	18.87	27.03	16.06	16.82	24.83
Understorey	125.9396	308.2466	12.70	13.30	1.83	13.18	24.01	23.31	11.14	22.27	21.95
Understorey	40.5240	24.6287	12.60	14.30	5.78	39.82	37.40	36.33	35.41	33.26	32.65
Understorey	88.8047	105.6513	12.60	13.70	3.52	11.83	22.35	21.17	8.32	19.61	18.57
Understorey	91.2037	102.8936	12.50	17.70	23.69	14.13	10.39	26.45	4.95	3.91	21.40
Understorey	88.3162	299.1715	12.50	16.10	14.46	23.37	30.77	29.15	20.19	28.14	26.91
Understorey	153.1372	315.8642	12.50	15.60	11.93	44.65	31.06	38.46	40.05	24.36	32.67
Understorey	118.0149	288.4724	12.50	14.50	6.96	17.31	20.15	26.14	11.82	14.97	22.29
Understorey	31.4812	22.4802	12.50	13.30	2.46	53.82	39.50	36.82	49.67	36.36	34.25
Understorey	115.9730	299.6119	12.50	13.00	1.49	7.34	19.95	24.72	5.99	17.87	22.34
Understorey	54.0895	201.3288	12.50	13.00	1.49	24.28	24.59	21.40	23.34	23.57	20.51
Understorey	139.3356	279.5432	12.50	13.00	1.49	26.37	17.01	14.11	23.15	13.45	11.19
Understorey	126.2905	311.3267	12.40	13.90	4.93	42.57	22.76	22.53	39.18	20.89	20.73
Understorey	95.9416	189.6047	12.40	13.80	4.56	53.64	35.40	42.26	52.31	34.36	41.68
Understorey	35.3360	345.8700	12.40	13.60	3.82	21.46	31.60	29.33	18.54	29.69	27.79
Understorey	139.5696	308.9642	12.30	15.30	11.31	65.68	38.12	29.86	60.85	32.89	25.14
Understorey	55.4502	201.9837	12.30	15.20	10.83	21.81	23.78	24.96	20.00	22.24	23.31
Understorey	122.6653	307.9420	12.30	13.60	4.16	31.64	23.56	23.35	29.55	21.85	21.87
Understorey	97.9545	92.0457	12.30	13.20	2.76	10.22	19.92	18.05	7.31	17.00	15.41
Understorey	38.1263	216.9562	12.30	12.50	0.57	3.06	4.34	13.76	0.73	2.48	12.33
Understorey	142.9518	349.1914	12.20	16.60	18.56	26.56	34.05	38.38	24.02	31.98	36.74
Understorey	32.6050	236.2749	12.20	13.80	5.24	28.45	21.35	30.88	27.56	20.40	29.93
Understorey	75.4293	37.2925	12.10	17.10	21.99	28.26	18.37	24.63	24.25	15.74	21.83
Understorey	112.4443	83.5527	12.10	14.50	8.46	37.51	27.32	23.99	35.86	25.81	22.00
Understorey	104.4505	289.2981	12.00	14.00	6.73	37.66	31.88	33.69	32.87	27.64	29.60
Understorey	88.1819	79.8523	12.00	14.00	6.73	23.12	25.85	22.68	20.84	22.46	19.45
Understorey	92.7514	193.1314	12.00	13.70	5.55	26.03	43.01	38.06	25.03	42.36	37.53
Understorey	148.0710	348.8360	12.00	13.20	3.72	23.03	41.96	33.27	21.56	40.73	32.11
Understorey	37.5003	185.3045	12.00	12.40	1.13	43.51	34.14	35.97	43.01	33.09	35.21
Understorey	78.3679	85.6816	12.00	12.30	0.84	32.24	36.62	31.37	30.99	35.47	29.61
Understorey	142.4374	294.2957	11.90	15.70	15.01	47.49	31.71	24.20	41.99	26.82	19.12
Understorey	147.3007	305.2111	11.90	15.20	12.48	37.62	37.57	38.87	30.12	32.48	33.91
Understorey	146.9909	128.4544	11.90	14.80	10.57	0.69	14.55	27.52	0.00	13.24	26.06
Understorey	102.8514	88.0285	11.90	12.80	2.68	18.65	17.77	22.52	16.88	15.89	20.54

Understorey	49.2017	360.9120	11.80	15.40	13.89	24.33	25.43	32.53	23.22	23.84	31.11
Understorey	146.5393	307.5849	11.80	14.80	10.97	60.08	33.74	38.60	53.53	28.63	34.24
Understorey	123.5705	294.6892	11.80	13.80	6.64	10.93	20.55	15.27	9.38	18.29	11.85
Understorey	86.9680	109.6915	11.80	13.00	3.66	8.28	19.95	22.19	4.94	17.42	20.19
Understorey	101.8404	93.0003	11.80	12.20	1.11	18.18	18.28	31.17	16.20	16.26	29.30
Understorey	167.5912	305.0912	11.70	15.70	15.89	10.54	22.00	25.56	5.29	17.14	20.92
Understorey	32.1500	349.8842	11.70	14.70	10.91	26.45	19.82	34.72	24.45	17.68	33.03
Understorey	97.3698	57.9924	11.70	14.40	9.54	22.11	22.94	20.60	19.77	19.95	18.37
Understorey	112.8769	135.7934	11.70	13.50	5.82	44.84	39.13	34.76	44.58	38.21	33.60
Understorey	148.2699	304.5716	11.70	13.20	4.70	38.09	37.64	38.89	33.22	33.86	35.23
Understorey	84.7496	89.2191	11.70	12.60	2.64	10.13	16.47	23.35	8.28	14.34	21.18
Understorey	105.0872	20.6413	11.70	11.90	0.54	26.01	36.65	35.15	22.28	33.85	32.55
Understorey	133.6193	136.0322	11.60	15.70	16.33	10.70	25.83	26.21	9.13	23.52	24.05
Understorey	140.9648	328.4648	11.60	13.00	4.30	16.99	34.02	31.50	13.89	30.77	29.00
Understorey	96.2546	95.1103	11.50	14.80	12.18	12.16	16.45	17.96	8.51	12.33	14.07
Understorey	154.0682	310.3276	11.50	14.60	11.23	11.06	43.30	31.23	4.42	37.96	26.37
Understorey	101.1852	62.6877	11.50	14.60	11.23	38.81	18.78	22.07	35.80	15.91	19.80
Understorey	135.1998	276.4335	11.50	14.60	11.23	28.75	15.78	21.05	25.77	12.29	17.53
Understorey	156.5536	276.3930	11.50	14.50	10.77	13.49	17.76	24.21	10.59	13.96	20.99
Understorey	122.3208	135.5315	11.50	14.40	10.31	41.21	32.62	26.46	39.21	30.81	24.60
Understorey	126.4707	116.5407	11.50	13.50	6.51	18.86	15.12	29.97	14.99	11.43	27.33
Understorey	161.0116	340.4505	11.50	13.30	5.74	24.56	22.36	27.72	23.74	20.58	25.70
Understorey	75.0698	82.9997	11.50	13.10	4.99	27.51	48.54	31.41	25.91	46.80	29.61
Understorey	88.3818	134.1576	11.50	12.50	2.92	42.37	38.61	26.54	41.83	37.98	25.88
Understorey	38.9421	214.9001	11.50	12.20	1.97	3.25	6.01	11.35	1.12	4.42	10.05
Understorey	61.9833	191.3936	11.50	11.80	0.80	16.80	23.59	29.53	15.97	23.02	28.84
Understorey	80.5123	40.9445	11.40	16.00	18.89	4.08	10.50	23.60	0.73	6.93	20.99
Understorey	112.9722	75.2406	11.40	15.80	17.77	5.05	24.76	20.04	3.63	23.05	17.69
Understorey	161.9498	29.5871	11.40	12.60	3.55	27.75	42.86	34.78	27.09	41.77	34.01
Understorey	142.1951	325.9980	11.40	12.60	3.55	25.20	31.37	27.01	22.18	28.61	24.07
Understorey	86.7842	101.6562	11.40	12.40	2.89	13.14	10.51	19.95	10.21	8.12	17.84
Understorey	72.6727	177.1623	11.40	11.80	1.08	69.35	42.28	34.33	68.99	41.67	33.90
Understorey	35.7181	177.4745	11.40	11.60	0.53	36.44	41.58	44.87	36.26	41.05	43.90
Understorey	37.8874	36.0932	11.30	13.40	6.81	24.86	33.08	37.75	23.14	31.62	36.11
Understorey	44.2337	20.8155	11.30	13.10	5.65	38.15	39.00	37.42	34.26	35.35	33.94
Understorey	119.1339	279.8198	11.30	12.10	2.24	28.37	18.10	28.13	23.19	13.96	25.11
Understorey	80.2564	32.7930	11.20	13.90	9.24	5.29	28.58	26.89	1.37	25.16	23.41
Understorey	76.2372	80.3590	11.20	13.10	5.99	52.54	33.91	36.22	51.02	31.66	34.05
Understorey	98.2350	281.9714	11.20	12.50	3.84	60.94	44.05	32.69	57.87	40.48	28.97
Understorey	161.1096	304.2646	11.20	12.50	3.84	14.93	18.73	31.56	10.18	14.88	27.76
Understorey	127.9975	109.2252	11.20	12.40	3.50	36.82	37.31	34.77	35.05	35.22	32.79
Understorey	139.2096	303.5053	11.10	14.10	10.50	56.85	45.16	32.66	52.61	41.38	28.84
Understorey	176.4095	313.0130	11.10	13.20	6.72	13.13	25.69	30.44	9.56	22.97	27.45
Understorey	97.3092	83.3779	11.10	13.00	5.95	8.90	15.92	17.86	6.27	13.52	15.38
Understorey	41.4951	32.8409	11.10	12.70	4.85	26.91	37.91	35.50	25.75	36.70	34.11
Understorey	147.6334	302.3395	11.10	12.00	2.51	37.51	38.03	33.35	33.16	34.79	30.08
Understorey	112.9325	293.6849	11.00	14.30	11.81	9.57	17.47	27.01	5.86	13.06	22.69
Understorey	113.0162	282.3820	11.00	14.30	11.81	31.39	33.01	25.44	24.85	27.86	21.20
Understorey	23.6081	165.4764	11.00	14.10	10.89	12.69	23.60	43.41	9.33	19.84	40.28
Understorey	175.6038	93.7710	11.00	13.70	9.12	24.30	17.65	30.82	21.63	15.46	29.12
Understorey	138.6118	61.5466	11.00	12.70	5.17	48.22	39.57	32.35	48.08	39.44	31.91

Understorey	66.5083	109.9209	11.00	12.60	4.81	0.97	48.17	39.42	0.00	47.61	38.75
Understorey	33.4329	210.4138	11.00	11.30	0.77	4.87	33.37	22.81	2.74	31.92	21.51
Understorey	166.5848	306.2900	10.90	14.70	14.15	11.19	14.69	26.08	6.57	9.76	21.52
Understorey	73.8862	182.9727	10.90	13.30	7.81	43.65	38.29	33.70	42.70	37.38	33.05
Understorey	77.7095	98.3673	10.90	12.40	4.43	25.49	15.39	30.21	23.74	13.71	28.76
Understorey	107.7172	292.3223	10.90	12.30	4.08	18.01	24.44	34.90	14.79	21.00	31.65
Understorey	146.4101	342.0399	10.90	12.20	3.75	26.21	28.95	27.36	24.23	27.32	26.01
Understorey	146.5622	286.1909	10.90	12.10	3.42	7.61	13.96	25.92	4.41	10.84	22.98
Understorey	93.3707	303.8125	10.90	12.00	3.10	17.74	26.37	29.63	15.17	24.55	27.83
Understorey	125.0620	132.0997	10.80	15.80	20.53	24.99	20.98	24.65	21.42	17.53	21.86
Understorey	151.2566	311.0286	10.80	13.60	9.42	23.73	43.42	33.85	18.78	38.99	29.47
Understorey	157.9404	338.4750	10.80	13.50	9.00	15.21	29.75	34.41	13.44	27.16	31.79
Understorey	121.9674	65.3945	10.80	12.50	5.09	5.87	37.19	33.22	5.69	36.80	32.76
Understorey	88.4046	68.9367	10.80	12.30	4.39	29.73	36.76	36.17	26.80	34.44	34.18
Understorey	75.3455	34.8940	10.80	12.20	4.05	28.62	27.51	24.41	26.11	25.57	22.53
Understorey	124.8135	306.0057	10.80	11.60	2.15	19.43	28.90	24.27	18.38	27.79	22.95
Understorey	76.5500	68.0891	10.80	11.40	1.58	54.83	57.25	39.25	52.42	55.88	37.96
Understorey	149.4381	258.0524	10.70	14.20	12.53	5.98	21.28	27.22	4.20	19.90	25.67
Understorey	126.1722	315.2615	10.70	12.50	5.41	33.43	20.03	22.53	31.57	18.66	21.20
Understorey	146.4931	337.3780	10.70	12.00	3.69	28.96	19.21	28.96	27.14	17.54	27.18
Understorey	146.8557	301.6497	10.70	11.70	2.74	37.53	43.03	33.48	33.41	39.79	30.46
Understorey	123.7382	119.7982	10.70	11.60	2.43	14.93	27.41	32.28	11.93	25.33	30.68
Understorey	158.0288	303.0820	10.70	11.50	2.14	25.69	31.74	33.31	22.22	28.88	30.26
Understorey	149.5861	319.7620	10.70	10.80	0.24	51.79	33.30	38.79	48.98	30.63	36.32
Understorey	125.2268	156.4960	10.60	15.80	21.47	29.56	41.93	44.88	28.44	41.43	44.46
Understorey	162.6073	312.2995	10.50	14.30	13.81	12.26	16.86	32.23	7.64	11.80	27.52
Understorey	123.7487	264.8910	10.50	14.20	13.33	16.80	40.75	30.17	15.02	38.05	27.65
Understorey	137.8573	116.5844	10.50	12.90	7.59	5.62	8.73	24.25	2.69	5.80	22.14
Understorey	161.7432	43.7440	10.50	11.90	3.96	8.81	17.02	25.18	7.84	16.10	24.41
Understorey	161.8547	326.0372	10.50	11.70	3.31	35.53	32.85	26.93	33.04	29.73	24.28
Understorey	83.3954	88.8297	10.50	11.20	1.81	8.66	26.91	25.79	7.49	25.97	24.55
Understorey	129.4973	129.2678	10.50	10.80	0.74	5.38	14.94	21.10	4.18	13.67	20.06
Understorey	90.2640	240.7940	10.40	13.40	10.03	31.37	34.29	35.17	30.03	32.66	33.79
Understorey	23.2408	180.1231	10.40	13.00	8.34	38.41	22.93	28.84	35.51	20.67	26.40
Understorey	122.2693	310.8499	10.40	11.40	2.67	31.24	22.69	24.81	30.37	21.65	23.82
Understorey	147.0849	340.9886	10.40	11.30	2.37	29.93	29.05	31.37	28.63	27.82	30.01
Understorey	153.4000	300.0000	10.40	11.30	2.37	52.90	30.15	31.34	50.91	27.01	28.82
Understorey	146.3448	302.8739	10.40	10.50	0.24	38.18	43.18	33.33	35.81	41.24	31.26
Understorey	93.8638	69.8207	10.30	13.20	9.53	16.94	17.03	32.82	13.12	13.98	30.56
Understorey	21.7070	147.3733	10.30	12.90	8.28	43.79	24.56	26.99	40.14	21.84	24.41
Understorey	90.3679	79.0958	10.30	12.30	5.97	24.26	13.98	22.74	22.19	11.50	20.45
Understorey	145.3430	270.9106	10.30	11.70	3.90	3.35	15.67	21.52	0.00	12.80	19.05
Understorey	145.7272	279.7500	10.30	11.40	2.95	5.51	15.33	20.81	2.45	12.36	18.33
Understorey	103.3170	302.4099	10.30	11.30	2.65	29.53	19.48	17.63	27.24	17.44	16.01
Understorey	49.7068	181.2197	10.30	11.20	2.35	33.17	31.74	33.04	32.46	31.25	32.70
Understorey	95.4903	57.9903	10.20	13.20	9.90	22.19	15.56	26.95	20.74	13.02	24.88
Understorey	28.7477	128.7477	10.20	12.60	7.43	22.58	29.81	35.15	20.33	28.48	33.68
Understorey	141.0939	327.7082	10.20	12.30	6.29	24.67	33.88	31.73	22.58	31.14	29.42
Understorey	128.8965	277.6282	10.20	11.80	4.52	29.42	25.26	21.22	27.23	22.27	18.25
Understorey	115.1575	283.1791	10.20	11.50	3.54	32.07	26.97	25.29	27.08	23.49	22.46
Understorey	158.8984	315.8270	10.20	11.40	3.23	29.30	21.51	29.72	25.80	18.56	26.87

Understorey	57.1000	249.7976	10.20	11.30	2.93	10.61	34.01	34.48	9.79	33.38	33.89
Understorey	40.9906	231.2973	10.20	11.20	2.63	5.55	16.52	19.46	5.31	15.88	18.55
Understorey	99.9774	176.7536	10.20	10.60	0.97	48.33	38.75	44.01	48.04	38.50	43.72
Understorey	145.6853	330.0160	10.20	10.40	0.47	34.09	35.86	26.78	31.60	33.72	25.13
Understorey	131.0610	334.8906	10.20	10.30	0.23	27.55	39.79	29.51	26.49	38.77	28.59
Understorey	34.4288	230.6654	10.10	13.90	13.47	1.18	20.83	23.37	0.00	19.47	21.94
Understorey	37.9118	24.2928	10.10	13.30	10.70	40.27	38.91	38.17	35.92	35.02	35.01
Understorey	140.8609	126.2901	10.10	13.20	10.26	2.10	4.06	18.85	0.55	2.06	16.81
Understorey	45.4999	356.9542	10.10	12.40	6.99	24.34	33.06	26.43	22.12	31.30	24.71
Understorey	146.5333	310.0325	10.10	12.00	5.52	58.44	45.41	38.66	54.86	41.57	35.48
Understorey	116.1711	121.5864	10.10	12.00	5.52	35.85	42.91	33.17	33.15	41.15	31.46
Understorey	147.1273	44.4946	10.10	11.80	4.83	12.94	24.85	26.95	12.05	23.76	26.18
Understorey	95.3020	104.8753	10.10	11.70	4.49	13.11	21.48	24.16	9.81	19.03	22.36
Understorey	26.5448	23.4920	10.10	11.30	3.20	34.01	43.61	35.90	32.13	41.70	34.51
Understorey	107.8808	292.0500	10.10	11.00	2.31	18.09	24.65	34.92	16.18	21.91	32.34
Understorey	95.0830	76.3371	10.10	10.90	2.03	10.13	17.25	14.01	8.59	15.63	12.50
Understorey	24.9105	186.1745	10.00	12.00	5.83	35.73	28.97	33.74	34.36	27.46	32.34
Understorey	123.4027	132.0803	10.00	12.00	5.83	26.43	25.16	25.01	24.45	23.30	23.42
Understorey	92.1602	101.5142	10.00	11.60	4.45	15.42	10.77	29.92	12.94	8.75	27.96
Understorey	124.0135	276.4137	10.00	11.20	3.18	31.40	25.84	20.51	27.70	22.84	17.93
Understorey	161.0461	279.6888	10.00	11.10	2.88	13.35	29.87	23.90	11.12	27.37	21.69
Understorey	146.3668	102.0471	10.00	11.00	2.58	46.24	36.56	48.02	45.65	35.88	47.11
Understorey	104.2501	101.1388	10.00	11.00	2.58	27.13	38.88	30.18	25.66	37.65	28.59
Understorey	124.6405	286.3212	10.00	10.80	2.01	15.45	19.54	24.81	13.03	17.49	22.71
Understorey	96.1193	198.5322	10.00	10.70	1.74	46.25	44.41	39.71	45.82	44.09	39.41
Understorey	97.9778	190.5044	10.00	10.50	1.21	25.63	40.22	43.72	25.34	39.87	43.46
Understorey	126.9544	146.6946	9.90	14.60	17.88	26.05	44.16	42.72	25.31	43.19	41.76
Understorey	63.8231	354.6141	9.90	12.30	7.27	9.84	23.77	26.82	9.08	23.37	26.18
Understorey	141.9476	36.8957	9.90	11.90	5.79	24.52	26.49	25.57	23.36	25.65	24.67
Understorey	32.9890	125.4187	9.90	11.70	5.09	46.43	39.32	32.66	45.33	38.13	31.61
Understorey	173.2169	84.3160	9.90	11.50	4.42	17.90	38.32	41.58	16.88	37.11	40.84
Understorey	141.8415	278.0400	9.90	11.30	3.77	26.74	16.82	14.06	24.61	14.15	11.54
Understorey	128.0541	123.8979	9.90	11.00	2.85	6.98	20.00	16.04	5.06	18.34	14.81
Understorey	31.5822	343.6380	9.90	10.90	2.56	30.76	23.91	38.47	29.49	23.00	37.57
Understorey	170.3240	327.0964	9.90	10.70	1.99	30.85	17.42	26.85	28.06	15.36	24.94
Understorey	139.0427	114.3385	9.80	13.00	10.48	5.07	13.63	29.67	2.27	10.59	27.44
Understorey	129.0138	115.3105	9.80	12.60	8.80	15.02	15.03	33.50	11.49	12.01	31.13
Understorey	57.0537	324.0106	9.80	11.10	3.43	23.89	26.49	32.59	22.90	25.38	31.66
Understorey	159.2307	313.6334	9.80	10.40	1.44	29.08	21.95	33.77	26.72	19.70	31.59
Understorey	176.4235	91.0233	9.80	10.30	1.18	12.03	16.95	39.63	11.21	16.49	39.11
Understorey	94.4810	114.6343	9.80	10.10	0.69	7.54	20.99	31.09	5.19	19.62	29.96
Understorey	87.8138	91.0863	9.70	14.40	17.67	14.26	16.98	23.74	12.48	13.09	20.35
Understorey	41.4364	354.5283	9.70	12.20	7.55	47.05	27.48	24.35	44.44	25.20	22.99
Understorey	150.6747	308.5735	9.70	11.70	5.70	36.28	50.45	34.42	32.31	47.04	31.05
Understorey	136.9501	111.9501	9.70	11.30	4.34	15.14	29.08	36.53	13.14	27.14	34.99
Understorey	102.7497	26.4314	9.70	11.20	4.02	46.22	39.58	34.79	44.81	37.47	32.69
Understorey	59.1320	182.9383	9.70	10.90	3.10	41.65	34.90	35.62	41.16	34.38	35.29
Understorey	129.0458	322.5136	9.70	10.00	0.68	12.09	24.75	30.02	11.58	23.77	29.08
Understorey	82.8057	104.0634	9.70	10.00	0.68	8.35	21.13	19.43	7.56	20.04	18.29
Understorey	164.7134	323.8869	9.60	12.90	10.77	36.01	23.35	21.96	31.55	20.06	18.86
Understorey	40.9655	344.9317	9.60	12.10	7.49	27.21	37.43	30.05	24.20	35.81	28.73

Understorey	175.0000	100.0000	9.60	12.00	7.11	28.64	36.39	27.82	27.29	35.00	26.52
Understorey	153.4110	301.8908	9.60	10.10	1.16	54.03	29.29	34.56	52.42	27.49	32.62
Understorey	83.5491	185.1884	9.60	9.70	0.22	27.08	34.09	42.77	26.73	33.77	42.49
Understorey	125.3240	278.0830	9.50	11.90	7.05	38.22	25.70	21.53	34.60	22.57	18.64
Understorey	94.4575	230.0550	9.50	11.80	6.68	18.19	24.80	32.68	16.54	23.67	31.52
Understorey	104.2646	286.1096	9.50	11.40	5.27	27.65	40.07	31.45	24.86	36.72	28.23
Understorey	170.4284	280.7603	9.50	10.80	3.34	27.29	28.47	21.59	25.82	27.24	19.95
Understorey	88.2317	106.9617	9.50	10.50	2.47	11.67	21.24	21.07	9.66	19.51	19.51
Understorey	146.6763	289.9588	9.50	10.50	2.47	9.46	32.13	21.02	6.90	29.57	18.95
Understorey	116.8257	88.7404	9.50	10.40	2.19	50.73	38.61	29.96	50.41	38.12	29.46
Understorey	130.2857	104.4352	9.50	10.20	1.66	33.82	45.74	34.45	33.07	45.13	33.61
Understorey	118.4553	281.6140	9.50	9.90	0.91	29.75	19.06	28.28	26.66	16.49	26.33
Understorey	122.1558	309.9988	9.50	9.60	0.22	31.07	22.67	25.27	30.70	22.14	24.72
Understorey	92.7827	36.3288	9.40	13.90	16.31	3.64	13.58	24.34	0.00	10.78	21.01
Understorey	86.2725	25.7882	9.40	13.30	13.35	6.88	22.07	23.73	1.78	17.58	20.06
Understorey	95.1801	44.0594	9.40	12.50	9.77	2.63	18.85	32.16	0.48	17.06	30.45
Understorey	84.0116	35.1858	9.40	12.20	8.54	6.90	12.70	21.87	3.26	9.98	19.55
Understorey	90.9166	70.1738	9.40	12.00	7.76	30.10	17.62	34.31	27.55	15.45	32.61
Understorey	37.8664	35.4936	9.40	11.70	6.63	38.12	29.74	36.05	36.95	28.80	34.98
Understorey	137.5759	121.1997	9.40	11.60	6.27	6.29	4.45	14.06	3.92	2.36	12.37
Understorey	105.7259	285.5458	9.40	11.30	5.22	28.60	46.56	29.23	25.52	43.11	26.18
Understorey	175.7147	108.1688	9.40	11.20	4.89	26.55	43.99	26.24	26.16	43.37	25.38
Understorey	99.1020	105.1656	9.40	11.10	4.56	21.40	34.37	27.03	18.81	32.26	25.37
Understorey	40.7325	217.6731	9.40	10.30	2.17	2.46	5.66	12.59	1.49	4.53	11.54
Understorey	86.0731	356.0122	9.40	10.00	1.39	33.05	29.26	36.43	32.70	28.89	36.11
Understorey	151.2655	328.4769	9.40	10.00	1.39	54.92	34.42	29.08	53.14	32.40	27.61
Understorey	118.1603	279.9204	9.40	9.70	0.66	28.63	18.13	28.31	25.87	15.69	26.45
Understorey	122.1975	304.5220	9.40	9.50	0.21	20.88	19.47	23.17	20.38	18.95	22.48
Understorey	89.6000	78.6373	9.40	9.50	0.21	24.57	13.97	22.77	23.60	12.43	21.49
Understorey	122.7191	130.9000	9.30	11.90	7.70	26.75	25.32	24.72	24.81	23.62	23.26
Understorey	97.7530	35.2530	9.30	10.70	3.58	26.13	39.00	29.54	25.29	37.96	28.38
Understorey	112.2156	56.6325	9.30	10.60	3.28	43.68	34.99	29.44	43.43	34.53	28.83
Understorey	114.7045	122.0488	9.30	10.50	2.99	35.62	48.49	32.96	34.16	47.74	32.05
Understorey	78.0902	30.1430	9.30	10.30	2.42	5.80	28.63	30.10	4.00	27.15	28.80
Understorey	59.5076	183.8660	9.30	10.00	1.63	25.03	28.25	40.09	24.87	27.84	39.82
Understorey	145.7214	292.0572	9.30	10.00	1.63	36.50	32.42	23.02	34.61	30.41	21.32
Understorey	59.3779	247.2111	9.30	9.90	1.37	49.72	44.40	34.33	49.11	43.59	33.74
Understorey	49.9676	179.9111	9.30	9.80	1.13	33.24	38.59	31.33	33.07	38.44	31.21
Understorey	39.5521	230.6382	9.30	9.80	1.13	1.27	16.55	22.27	1.27	16.13	21.61
Understorey	175.1109	330.3530	9.30	9.80	1.13	40.01	19.25	21.03	38.93	17.91	19.62
Understorey	37.2930	22.1043	9.30	9.60	0.66	40.85	33.66	45.96	39.69	32.76	45.07
Understorey	89.0170	60.7939	9.20	13.50	15.10	21.85	30.29	28.90	19.06	27.38	26.29
Understorey	138.1749	290.4231	9.20	11.70	7.26	15.96	25.74	30.33	14.56	23.30	27.82
Understorey	125.9393	122.2549	9.20	11.20	5.48	7.08	26.00	20.71	4.56	23.90	19.29
Understorey	21.7252	377.9975	9.20	10.70	3.85	9.75	35.98	31.24	8.10	34.97	30.54
Understorey	89.7051	32.6901	9.20	10.30	2.68	21.44	15.77	11.18	20.03	14.27	9.56
Understorey	138.7267	305.7711	9.20	9.90	1.61	50.39	42.08	34.48	49.13	41.14	33.40
Understorey	29.2596	114.4863	9.10	11.70	7.58	15.55	40.19	41.25	14.73	39.05	40.17
Understorey	153.4741	306.5338	9.10	11.70	7.58	37.71	47.24	32.50	33.39	43.46	29.21
Understorey	162.5000	324.1000	9.10	11.60	7.21	36.43	30.32	25.46	33.39	27.29	22.73
Understorey	119.8385	116.1588	9.10	11.20	5.77	28.34	46.21	39.42	26.40	44.67	38.07

Understorey	125.3393	79.1717	9.10	10.60	3.82	31.98	27.40	28.61	31.67	27.15	28.30
Understorey	20.1324	329.7693	9.10	10.30	2.94	26.25	30.42	29.80	26.25	29.91	29.26
Understorey	52.9129	323.0036	9.10	10.30	2.94	17.97	36.42	28.60	16.97	35.83	27.87
Understorey	123.6983	125.1955	9.10	10.30	2.94	26.95	23.74	23.29	26.28	22.67	22.43
Understorey	32.1673	344.1836	9.10	10.10	2.38	36.33	25.98	37.21	35.61	25.32	36.47
Understorey	118.5859	278.6568	9.10	10.00	2.11	28.29	18.27	27.90	25.14	15.36	25.81
Understorey	66.2901	108.9289	9.10	9.90	1.85	15.29	51.84	36.31	14.57	51.44	35.89
Understorey	95.9053	299.0689	9.10	9.80	1.59	13.96	33.85	30.97	12.05	32.10	29.40
Understorey	145.9709	306.8596	9.10	9.60	1.11	60.54	40.72	38.57	59.29	39.70	37.54
Understorey	149.6091	338.9868	9.10	9.50	0.87	29.91	24.04	34.50	28.98	23.05	33.46
Understorey	87.7059	136.7982	9.10	9.40	0.64	42.90	31.84	31.16	42.57	31.53	30.73
Understorey	142.3753	295.3020	9.10	9.30	0.42	47.53	31.87	26.07	46.30	30.92	25.18
Understorey	91.3577	32.9094	9.00	13.90	17.98	20.27	15.43	18.59	16.81	11.14	14.54
Understorey	135.9314	139.4275	9.00	13.70	16.93	21.82	33.94	30.62	21.55	32.73	29.44
Understorey	62.9292	45.6888	9.00	12.20	9.91	41.85	49.64	43.11	41.59	49.09	42.17
Understorey	43.4101	342.1175	9.00	12.20	9.91	34.23	30.49	33.79	31.55	28.33	32.30
Understorey	134.8940	125.3455	9.00	12.20	9.91	5.21	5.79	16.65	2.41	3.28	14.78
Understorey	20.4065	143.4580	9.00	11.10	5.73	23.05	33.08	27.11	20.97	31.54	25.63
Understorey	39.0987	343.0753	9.00	11.10	5.73	16.88	37.27	25.56	14.83	35.87	24.36
Understorey	157.2000	262.5000	9.00	11.00	5.39	12.79	27.03	31.38	11.00	25.67	30.09
Understorey	159.0849	302.7775	9.00	10.70	4.41	24.76	31.83	31.40	22.41	29.59	28.93
Understorey	136.4495	102.8547	9.00	10.50	3.79	62.13	43.96	38.46	61.05	42.90	37.74
Understorey	91.0946	107.3701	9.00	10.50	3.79	11.55	9.05	25.73	9.38	7.32	24.29
Understorey	99.0471	97.5377	9.00	10.50	3.79	10.59	24.76	25.56	9.38	23.18	24.12
Understorey	101.2302	51.8239	9.00	10.40	3.49	21.90	35.36	29.21	21.47	34.69	28.53
Understorey	128.7094	318.4363	9.00	10.40	3.49	31.72	35.72	21.14	30.82	34.44	19.89
Understorey	81.0073	51.0593	9.00	10.30	3.19	58.78	28.24	23.03	57.95	27.13	21.83
Understorey	42.6289	198.0159	9.00	10.20	2.91	31.53	24.88	22.97	30.91	24.28	22.33
Understorey	39.9548	206.3950	9.00	10.20	2.91	7.08	20.38	21.08	5.88	19.08	19.96
Understorey	91.0732	119.9915	9.00	10.10	2.63	10.89	27.79	31.89	9.07	26.50	30.83
Understorey	132.2008	281.0422	9.00	10.10	2.63	29.03	24.09	22.09	28.35	22.64	19.81
Understorey	107.0767	102.1636	9.00	10.00	2.36	44.48	46.67	33.96	44.17	45.80	32.94
Understorey	28.6660	212.7848	9.00	10.00	2.36	18.45	28.57	28.61	16.74	27.51	27.60
Understorey	99.7136	298.8894	9.00	10.00	2.36	13.20	20.07	28.08	11.15	18.32	26.50
Understorey	96.2419	22.9182	9.00	9.80	1.83	23.94	27.01	31.94	21.78	25.47	30.66
Understorey	107.6937	281.2303	9.00	9.70	1.58	64.55	32.69	27.75	61.65	29.99	25.54
Understorey	139.6666	337.8820	9.00	9.50	1.09	23.70	26.08	31.44	23.07	25.21	30.70
Understorey	28.9603	213.5612	9.00	9.50	1.09	17.71	28.58	28.53	16.69	27.72	27.69
Understorey	179.7279	99.5024	9.00	9.40	0.86	48.09	23.08	25.01	47.87	22.67	24.60
Understorey	94.3567	81.2830	9.00	9.40	0.86	8.08	15.72	18.05	7.90	14.85	16.98
Understorey	79.5871	86.9498	9.00	9.30	0.64	7.84	31.89	32.17	7.12	31.51	31.46
Understorey	90.3988	123.3184	9.00	9.30	0.64	20.92	26.56	31.41	19.80	25.74	30.77
Understorey	89.0170	298.2939	9.00	9.30	0.64	23.77	31.28	27.02	22.20	29.63	25.72
Understorey	67.0831	112.5919	9.00	9.20	0.42	1.32	31.19	51.58	0.64	30.65	51.27
Understorey	25.1873	225.4636	8.90	11.90	9.02	33.09	31.49	29.68	32.28	30.68	28.50
Understorey	40.2391	340.9435	8.90	10.90	5.34	29.63	39.77	30.61	28.16	38.50	29.53
Understorey	95.9416	52.1047	8.90	10.80	5.01	11.20	19.67	24.33	10.37	18.67	23.14
Understorey	84.9622	283.6600	8.90	10.40	3.75	46.50	29.61	40.05	44.36	27.49	38.30
Understorey	117.3699	324.5534	8.90	10.40	3.75	30.24	31.03	30.04	30.24	30.47	29.46
Understorey	53.0311	314.2500	8.90	10.30	3.46	28.18	33.04	29.33	27.65	32.74	28.82
Understorey	23.5262	369.9373	8.90	10.20	3.17	16.26	23.41	37.46	14.98	22.45	36.81

Understorey	122.5968	137.9234	8.90	10.20	3.17	21.01	32.86	30.27	21.01	32.86	29.89
Understorey	55.9058	229.2908	8.90	9.60	1.56	18.31	33.65	29.56	17.47	33.00	28.86
Understorey	153.7750	322.0833	8.90	9.50	1.32	30.44	44.76	28.81	28.73	43.15	27.52
Understorey	118.0149	275.9724	8.90	9.40	1.08	28.31	16.77	32.61	25.23	14.36	30.94
Understorey	75.2355	29.4938	8.90	9.10	0.41	34.17	37.11	34.68	33.58	36.62	33.96
Understorey	85.0725	360.4304	8.90	9.10	0.41	52.09	26.53	34.06	51.79	26.22	33.79
Understorey	140.2444	361.8873	8.90	9.00	0.20	33.68	39.55	39.45	33.37	39.41	39.20
Understorey	123.8979	278.0541	8.90	9.00	0.20	17.36	26.30	21.57	16.11	24.78	19.94
Understorey	89.7000	45.7104	8.80	12.40	11.47	2.06	13.78	24.84	0.87	11.72	22.95
Understorey	88.6697	30.0667	8.80	12.10	10.18	20.48	13.49	18.96	19.07	10.36	16.14
Understorey	160.1766	306.3590	8.80	11.60	8.17	15.19	17.50	35.05	11.26	14.24	31.80
Understorey	30.6704	166.2532	8.80	11.00	5.97	10.45	50.05	34.41	9.83	48.92	33.11
Understorey	94.1553	293.7062	8.80	10.90	5.63	39.20	35.83	28.53	36.99	33.23	26.44
Understorey	90.3983	252.6096	8.80	10.90	5.63	56.70	30.67	22.22	55.83	29.37	20.78
Understorey	23.2756	24.0554	8.80	10.30	3.72	44.87	40.90	33.03	44.71	40.03	32.26
Understorey	122.4452	251.0453	8.80	10.30	3.72	33.60	24.23	25.66	33.06	23.41	24.51
Understorey	74.3794	199.3794	8.80	10.10	3.14	41.05	32.87	35.18	41.05	32.82	34.79
Understorey	20.7492	154.2943	8.80	10.10	3.14	43.94	33.06	32.49	43.34	32.10	31.66
Understorey	98.0374	289.3580	8.80	10.10	3.14	60.87	34.35	31.31	59.00	32.49	28.74
Understorey	150.0000	269.4000	8.80	10.00	2.85	4.02	10.30	27.28	1.46	8.24	25.66
Understorey	155.0372	275.7978	8.80	9.90	2.58	13.94	17.78	21.38	11.68	15.64	19.55
Understorey	132.5612	258.3975	8.80	9.80	2.31	31.12	16.79	29.78	30.33	15.69	28.92
Understorey	109.8501	295.1958	8.80	9.70	2.05	18.17	19.02	21.25	16.77	17.33	19.66
Understorey	41.5688	219.2716	8.80	9.10	0.62	1.66	4.86	9.41	1.03	4.25	8.90
Understorey	72.5070	181.2530	8.80	8.90	0.20	79.68	44.38	37.68	79.52	44.30	37.57
Understorey	87.5000	21.7000	8.80	8.90	0.20	7.03	22.64	33.67	6.22	21.99	32.92
Understorey	102.7170	264.8618	8.70	11.10	6.62	17.71	23.72	29.91	15.84	21.48	27.26
Understorey	91.0507	42.2119	8.70	11.10	6.62	3.43	3.46	24.02	2.11	1.60	22.43
Understorey	142.6704	321.6387	8.70	10.40	4.29	33.80	36.30	25.35	31.63	34.56	23.38
Understorey	70.7593	64.0308	8.70	10.20	3.69	53.72	44.06	42.90	53.03	43.06	42.05
Understorey	135.6480	308.9347	8.70	10.10	3.39	32.07	41.81	34.48	31.38	40.58	32.94
Understorey	164.3628	307.4713	8.70	9.90	2.83	12.33	9.34	31.50	11.18	7.44	29.43
Understorey	79.5648	66.9858	8.70	9.80	2.55	40.76	62.09	37.14	38.92	61.18	36.14
Understorey	126.2439	336.8348	8.70	9.70	2.29	34.81	38.64	25.03	34.07	38.12	24.50
Understorey	133.4960	116.2827	8.70	9.60	2.03	7.50	14.93	20.41	6.48	14.30	19.78
Understorey	32.0513	364.3894	8.70	9.50	1.78	21.84	30.47	26.07	21.14	29.31	25.12
Understorey	98.2850	84.5417	8.70	9.50	1.78	8.86	15.33	18.12	7.89	14.46	17.07
Understorey	48.4802	349.2748	8.70	9.40	1.53	27.99	35.26	36.62	26.88	34.07	35.63
Understorey	121.8434	122.5195	8.70	9.40	1.53	25.54	25.50	30.94	25.14	24.79	30.50
Understorey	85.6219	183.0041	8.70	9.30	1.29	15.80	33.86	32.64	15.67	33.57	32.41
Understorey	34.7097	23.6697	8.70	9.00	0.62	40.39	35.69	40.63	39.27	34.86	39.90
Understorey	133.4961	348.3745	8.70	9.00	0.62	79.73	40.27	38.88	79.73	39.94	38.39
Understorey	29.7543	206.3092	8.70	9.00	0.62	35.67	33.46	25.81	34.99	32.78	25.18
Understorey	82.0989	68.3519	8.70	8.80	0.20	53.97	59.53	37.80	53.21	59.03	37.29
Understorey	97.0252	119.2942	8.70	8.80	0.20	18.57	23.89	34.20	18.09	23.38	33.74
Understorey	157.6492	109.1159	8.60	11.70	9.22	74.57	37.77	40.67	74.32	37.06	40.00
Understorey	101.6357	67.1189	8.60	11.50	8.43	13.10	20.63	15.66	11.56	19.31	14.18
Understorey	168.0867	298.0071	8.60	11.00	6.57	28.90	26.33	20.83	27.29	24.32	18.55
Understorey	122.7982	286.0434	8.60	10.80	5.87	15.12	25.73	24.43	12.68	23.65	22.27
Understorey	25.0837	114.0978	8.60	10.40	4.56	21.21	27.22	39.85	20.41	26.28	38.82
Understorey	156.1194	27.4724	8.60	10.30	4.25	29.87	41.20	33.34	29.26	40.46	32.75

Understorey	21.9707	163.2454	8.60	10.20	3.95	14.10	30.87	43.86	12.57	29.68	42.65
Understorey	70.9155	315.0729	8.60	10.20	3.95	30.80	32.42	24.47	30.30	32.14	24.03
Understorey	22.1290	112.7782	8.60	10.10	3.65	10.23	20.89	37.16	9.91	19.65	36.12
Understorey	109.6837	277.0583	8.60	9.80	2.80	64.83	31.99	23.64	60.70	29.09	21.42
Understorey	76.0160	69.7290	8.60	9.70	2.53	79.76	53.40	39.55	78.83	52.49	38.76
Understorey	155.9667	288.7683	8.60	9.60	2.27	25.52	19.39	25.03	23.85	17.82	23.36
Understorey	51.7500	128.0311	8.60	9.50	2.01	53.55	53.20	46.23	53.55	52.98	46.11
Understorey	133.6060	123.9205	8.60	9.40	1.76	6.03	5.52	12.12	5.66	5.17	11.74
Understorey	76.3361	122.0066	8.60	9.20	1.28	66.83	34.92	36.97	66.83	34.61	36.31
Understorey	171.9292	276.1578	8.60	9.10	1.05	30.31	28.58	22.60	29.44	27.67	21.78
Understorey	161.5000	325.0000	8.60	9.00	0.83	36.40	32.81	25.45	34.82	31.21	24.13
Understorey	79.8481	71.2462	8.60	8.90	0.61	28.38	48.78	37.44	27.42	48.35	36.93
Understorey	41.4744	31.3604	8.60	8.90	0.61	27.25	38.06	34.24	26.77	37.49	33.87
Understorey	24.9182	131.4645	8.50	11.60	9.15	1.97	28.43	28.55	0.40	26.85	27.19
Understorey	166.0427	304.0754	8.50	11.60	9.15	11.09	27.15	26.35	8.04	24.13	23.25
Understorey	74.0547	61.3547	8.50	10.80	6.17	61.60	44.68	45.50	60.25	43.13	44.40
Understorey	175.8893	89.9432	8.50	10.60	5.49	11.43	20.60	44.52	10.57	20.02	43.93
Understorey	72.5377	49.0471	8.50	10.60	5.49	21.93	33.11	40.26	20.74	32.10	39.24
Understorey	139.2051	323.2658	8.50	10.50	5.16	43.64	28.31	28.57	41.98	25.91	26.68
Understorey	25.5839	244.1745	8.50	10.00	3.62	59.78	36.89	36.11	59.08	36.28	35.67
Understorey	106.5818	53.8000	8.50	10.00	3.62	33.08	30.60	33.44	32.91	30.21	33.00
Understorey	159.1734	286.7414	8.50	10.00	3.62	32.17	21.17	31.22	30.27	18.88	29.27
Understorey	145.1838	324.7967	8.50	10.00	3.62	40.98	38.84	27.48	38.68	37.37	25.86
Understorey	64.2365	72.3481	8.50	9.70	2.77	16.03	30.18	41.91	15.64	29.84	41.56
Understorey	102.6355	289.4860	8.50	9.30	1.74	37.51	31.14	35.72	36.49	29.44	33.85
Understorey	97.7436	263.5766	8.50	9.30	1.74	10.25	23.68	28.35	8.95	22.16	26.83
Understorey	88.7256	40.1283	8.50	9.10	1.27	4.39	4.26	15.30	4.03	3.70	14.76
Understorey	108.7391	103.1808	8.50	8.70	0.39	48.07	44.77	34.33	47.94	44.42	33.81
Understorey	164.0391	304.2286	8.40	11.30	8.30	10.95	26.67	33.48	9.04	24.38	30.80
Understorey	163.0414	94.3787	8.40	10.90	6.82	32.96	37.55	40.61	32.48	36.86	40.12
Understorey	171.7520	84.4149	8.40	10.80	6.46	17.47	24.27	44.99	16.74	23.67	44.55
Understorey	152.5432	263.0406	8.40	10.80	6.46	2.68	19.83	31.77	0.81	18.12	30.46
Understorey	76.0846	271.3337	8.40	10.70	6.12	26.01	29.17	31.70	24.40	27.80	30.29
Understorey	174.7473	326.9398	8.40	9.60	2.75	28.90	20.37	25.42	27.64	18.65	24.10
Understorey	104.0496	85.5495	8.40	9.50	2.48	22.00	17.25	22.22	21.11	16.59	21.39
Understorey	158.6011	343.5226	8.40	9.00	1.25	25.66	16.11	25.83	24.84	15.29	25.13
Understorey	112.5000	278.7000	8.40	9.00	1.25	28.47	33.17	21.72	25.52	31.08	20.01
Understorey	63.7515	20.4015	8.40	8.90	1.03	58.30	64.49	46.11	57.51	63.99	45.58
Understorey	75.1675	372.0985	8.40	8.90	1.03	33.87	32.17	33.58	33.60	31.86	33.41
Understorey	178.3829	88.7313	8.40	8.70	0.60	5.00	28.92	41.88	4.85	28.78	41.75
Understorey	109.9864	299.6145	8.40	8.70	0.60	32.84	17.75	24.05	32.41	17.14	23.22
Understorey	21.5654	375.7931	8.40	8.60	0.39	14.98	39.07	33.98	14.39	38.62	33.70
Understorey	70.2225	106.0335	8.40	8.60	0.39	2.16	32.89	31.01	1.83	32.68	30.67
Understorey	59.7830	321.9474	8.30	11.20	8.23	23.98	24.70	32.00	23.00	23.39	30.96
Understorey	138.0540	280.2710	8.30	11.20	8.23	26.44	17.00	15.88	24.57	14.63	13.53
Understorey	47.8945	347.8945	8.30	11.00	7.48	28.71	28.84	37.64	27.19	27.30	36.35
Understorey	83.4562	268.2038	8.30	10.50	5.73	10.75	22.21	38.39	8.44	20.38	36.75
Understorey	68.3303	49.4540	8.30	10.20	4.75	21.26	46.98	45.48	21.09	46.38	44.95
Understorey	172.3641	335.2146	8.30	9.80	3.55	28.13	33.24	23.57	26.89	31.89	22.23
Understorey	30.1998	203.3768	8.30	9.70	3.27	56.00	29.42	28.76	55.32	28.30	27.82
Understorey	81.8692	66.7923	8.30	9.50	2.72	41.18	59.06	37.74	39.95	58.09	36.91

Understorey	115.7572	114.4571	8.30	9.30	2.20	49.89	45.27	41.67	49.13	44.70	41.16
Understorey	91.7301	284.5009	8.30	9.30	2.20	38.70	42.51	33.84	36.98	40.86	32.17
Understorey	146.1988	302.9952	8.30	9.00	1.47	38.26	42.99	33.35	37.55	42.33	32.60
Understorey	148.2812	342.9933	8.30	8.90	1.24	29.82	27.61	29.24	29.37	27.08	28.70
Understorey	37.6047	243.4991	8.30	8.80	1.02	16.89	43.18	29.83	16.89	42.90	29.60
Understorey	99.2991	352.1868	8.30	8.70	0.80	41.46	38.40	43.22	41.46	38.40	43.18
Understorey	95.7006	70.8450	8.30	8.70	0.80	17.62	17.09	20.37	16.72	16.33	19.86
Understorey	73.3905	215.1146	8.30	8.60	0.59	47.22	59.41	41.25	47.22	59.27	41.15
Understorey	86.3017	68.5092	8.30	8.60	0.59	29.11	42.87	38.35	28.51	42.36	37.85
Understorey	100.6526	304.9572	8.30	8.50	0.39	29.48	18.25	24.73	28.99	17.79	24.14
Understorey	155.2986	39.8591	8.30	8.40	0.19	13.47	26.53	22.34	12.84	26.25	22.14
Understorey	106.0335	245.2225	8.20	11.80	10.99	18.38	25.54	26.52	16.30	24.09	24.92
Understorey	178.8091	111.0626	8.20	11.30	8.94	31.29	36.47	33.78	30.80	35.60	33.04
Understorey	95.1492	309.1159	8.20	11.00	7.79	2.55	32.43	27.12	1.27	31.22	25.97
Understorey	78.7613	270.5661	8.20	10.80	7.06	19.09	27.63	37.39	16.71	25.81	35.81
Understorey	147.1000	112.5000	8.20	10.50	6.01	25.22	26.06	34.02	24.57	25.24	33.16
Understorey	38.9940	26.8783	8.20	10.40	5.68	40.41	51.57	32.45	38.86	50.30	31.31
Understorey	25.0693	125.8789	8.20	9.90	4.10	2.27	19.72	29.00	1.63	18.97	28.27
Understorey	98.1703	62.7163	8.20	9.90	4.10	23.26	18.89	21.14	21.95	17.94	20.13
Understorey	160.2748	41.1385	8.20	9.80	3.81	28.10	22.44	31.94	27.38	21.63	31.38
Understorey	157.5175	40.2362	8.20	9.50	2.96	20.49	19.93	22.31	19.78	19.22	21.76
Understorey	169.2550	278.9000	8.20	9.50	2.96	24.86	28.53	18.54	23.62	27.59	17.11
Understorey	93.1479	117.5854	8.20	9.00	1.69	3.65	26.66	32.05	2.77	26.06	31.54
Understorey	150.0282	298.3906	8.20	8.80	1.23	54.75	29.03	30.03	54.41	28.38	29.44
Understorey	24.3198	208.9069	8.20	8.70	1.00	48.04	33.30	28.31	47.86	32.81	27.68
Understorey	70.0702	245.6180	8.20	8.50	0.58	64.49	49.82	40.98	64.33	49.61	40.55
Understorey	124.3289	69.6075	8.20	8.50	0.58	22.09	42.43	32.53	21.96	42.37	32.46
Understorey	151.7554	59.0310	8.20	8.30	0.19	48.68	43.30	35.56	48.68	43.18	35.37
Understorey	32.7533	152.6697	8.10	10.40	5.96	68.82	39.58	33.70	68.14	38.78	32.82
Understorey	53.1712	321.4551	8.10	10.20	5.30	17.80	33.04	29.19	17.50	32.76	28.56
Understorey	100.3910	231.8543	8.10	10.20	5.30	18.59	17.77	28.50	16.93	16.37	27.48
Understorey	131.8220	116.2815	8.10	10.10	4.98	7.81	12.70	25.54	6.33	11.83	24.70
Understorey	46.8358	203.5837	8.10	9.60	3.48	6.75	14.44	21.53	6.06	13.92	20.91
Understorey	93.1421	293.9905	8.10	9.30	2.67	39.69	26.46	31.75	38.39	24.51	30.20
Understorey	159.6660	294.2682	8.10	9.10	2.15	33.77	23.20	27.14	33.31	22.47	26.22
Understorey	59.8733	234.2070	8.10	9.00	1.91	54.11	33.84	31.55	53.53	33.30	31.12
Understorey	84.3678	285.4040	8.10	8.90	1.67	46.55	36.50	39.98	44.85	34.90	38.88
Understorey	71.5631	241.7262	8.10	8.90	1.67	40.53	51.63	38.68	40.03	51.29	38.27
Understorey	38.3153	370.2573	8.10	8.90	1.67	42.77	32.01	27.45	42.47	31.49	26.92
Understorey	152.1012	275.9355	8.10	8.80	1.44	4.01	9.99	22.25	2.46	8.99	21.20
Understorey	169.3150	276.0794	8.10	8.70	1.21	41.09	28.92	20.85	40.55	28.18	20.05
Understorey	101.7365	22.3481	8.10	8.60	0.99	44.93	29.85	33.32	44.29	29.05	32.75
Understorey	30.8261	214.6205	8.10	8.50	0.78	9.58	28.65	28.69	9.12	28.19	28.24
Understorey	100.8205	304.2210	8.10	8.40	0.58	29.95	18.35	24.81	29.63	17.97	24.31
Understorey	140.8457	303.7157	8.10	8.30	0.38	37.29	42.90	34.77	36.85	42.50	34.44
Understorey	45.2735	181.7574	8.10	8.20	0.19	37.38	33.77	37.19	37.38	33.77	37.15
Understorey	78.4679	42.2732	8.00	11.00	8.41	4.92	24.27	26.31	2.60	22.53	25.10
Understorey	85.2413	267.2756	8.00	10.90	8.04	10.44	22.30	37.43	7.56	19.99	35.42
Understorey	81.8989	37.6204	8.00	10.10	5.25	4.74	4.63	28.97	3.70	3.52	28.11
Understorey	150.4808	317.0748	8.00	10.00	4.94	52.15	30.69	34.86	50.61	28.86	33.16
Understorey	140.7746	323.2106	8.00	10.00	4.94	33.03	28.75	28.93	32.17	27.24	27.47

Understorey	139.5667	128.3074	8.00	10.00	4.94	2.98	4.42	15.21	2.58	3.98	14.71
Understorey	133.5001	264.7776	8.00	9.90	4.63	53.38	29.06	21.54	51.88	27.46	20.25
Understorey	151.5976	296.5602	8.00	9.80	4.32	55.09	30.71	24.53	53.32	28.94	23.04
Understorey	126.2108	121.1153	8.00	9.70	4.02	5.83	25.60	25.33	4.96	24.67	24.72
Understorey	147.8231	279.5961	8.00	9.70	4.02	7.13	14.99	18.03	4.94	13.22	16.31
Understorey	135.0377	111.5471	8.00	9.50	3.45	15.78	29.08	26.40	15.21	28.45	25.84
Understorey	151.0181	276.8367	8.00	9.50	3.45	5.41	9.96	22.68	3.18	8.40	21.07
Understorey	154.3069	29.4599	8.00	9.10	2.38	26.78	41.40	28.67	26.59	41.00	28.37
Understorey	150.6228	349.3837	8.00	9.00	2.13	25.58	27.14	28.49	25.03	26.61	27.87
Understorey	162.5000	321.3000	8.00	9.00	2.13	43.44	27.46	26.13	41.56	25.83	24.84
Understorey	151.8755	54.4184	8.00	8.80	1.65	56.64	34.88	36.16	56.13	34.30	35.83
Understorey	151.3101	52.8096	8.00	8.80	1.65	45.29	33.81	36.15	44.64	33.23	35.82
Understorey	176.6483	341.5796	8.00	8.80	1.65	30.56	24.90	26.73	29.87	24.29	26.28
Understorey	130.4992	262.5960	8.00	8.60	1.20	32.46	35.33	28.98	31.28	34.45	28.28
Understorey	128.8000	319.0818	8.00	8.50	0.98	13.42	35.76	21.17	12.96	35.24	20.64
Understorey	113.8357	94.3714	8.00	8.40	0.77	39.08	30.24	42.82	38.81	29.98	42.64
Understorey	83.8331	300.1149	8.00	8.30	0.57	22.83	24.97	33.71	21.64	24.20	33.13
Understorey	144.0388	359.3383	8.00	8.20	0.37	31.80	31.40	32.39	31.67	31.28	32.28
Understorey	146.8204	278.0284	8.00	8.20	0.37	6.69	14.91	18.25	6.08	14.18	17.35
Understorey	145.8183	276.1691	8.00	8.20	0.37	5.53	14.67	14.87	4.78	13.73	14.01
Understorey	103.0058	92.9226	8.00	8.10	0.18	28.51	17.13	31.83	28.37	16.94	31.64
Understorey	108.0580	283.3443	7.90	12.80	16.77	36.83	37.21	29.80	31.21	32.75	25.81
Understorey	31.5596	128.6361	7.90	10.50	6.88	22.51	25.18	36.44	21.40	24.53	35.70
Understorey	147.5663	310.9854	7.90	10.50	6.88	57.42	45.49	36.18	55.56	43.18	33.87
Understorey	100.0631	223.0417	7.90	9.50	3.70	26.18	32.12	38.37	25.49	31.53	37.73
Understorey	121.5960	267.5420	7.90	9.50	3.70	21.08	35.98	29.18	19.55	33.97	27.51
Understorey	116.4034	159.4236	7.90	9.40	3.42	28.56	33.53	39.55	28.56	33.46	39.46
Understorey	21.1217	373.1469	7.90	9.20	2.87	14.82	32.66	35.56	13.58	31.92	35.06
Understorey	51.5031	235.6949	7.90	9.20	2.87	44.51	32.64	22.07	44.01	32.10	21.59
Understorey	115.8442	115.5111	7.90	9.10	2.61	50.99	45.41	38.00	50.43	44.93	37.59
Understorey	77.1880	37.4086	7.90	9.10	2.61	4.53	14.35	24.90	3.94	13.92	24.44
Understorey	41.6346	26.5049	7.90	8.90	2.11	39.43	40.05	38.95	38.67	39.48	38.41
Understorey	105.3683	282.9588	7.90	8.20	0.56	45.31	46.13	31.79	44.41	44.92	30.67
Understorey	96.2424	111.1898	7.90	8.10	0.37	8.34	27.83	29.30	7.70	27.54	29.07
Understorey	99.0258	186.5258	7.90	8.00	0.18	57.43	38.84	35.75	57.16	38.72	35.65
Understorey	90.3730	107.8934	7.90	8.00	0.18	11.87	21.12	25.82	11.56	20.92	25.70
Understorey	109.1208	289.7741	7.80	12.10	13.75	10.65	24.85	35.12	6.75	21.17	31.88
Understorey	163.7671	320.5003	7.80	12.00	13.29	41.56	24.56	22.26	37.22	20.91	19.05
Understorey	103.4717	228.0179	7.80	10.50	7.18	18.16	31.90	25.24	16.65	30.50	24.17
Understorey	58.2798	338.0790	7.80	10.40	6.83	50.55	27.41	26.36	49.27	26.34	25.47
Understorey	35.2801	156.9340	7.80	10.30	6.48	37.19	53.43	33.85	36.78	52.76	33.25
Understorey	74.1260	36.4248	7.80	10.30	6.48	28.72	18.80	31.61	27.16	17.67	30.46
Understorey	86.4783	256.1031	7.80	9.60	4.24	41.82	33.53	26.96	40.47	32.32	25.86
Understorey	142.4031	272.1229	7.80	9.60	4.24	25.86	15.88	19.08	24.04	14.27	17.57
Understorey	92.1942	31.2294	7.80	9.50	3.95	20.60	15.52	19.05	19.75	14.32	18.03
Understorey	162.1445	93.1631	7.80	9.40	3.66	32.78	39.84	30.66	32.78	39.84	30.55
Understorey	70.9121	314.2880	7.80	9.40	3.66	41.86	32.44	24.51	41.58	32.25	24.16
Understorey	157.8924	289.3221	7.80	9.40	3.66	24.72	22.07	25.30	23.66	20.76	23.81
Understorey	88.6263	69.6114	7.80	9.30	3.38	30.27	36.84	36.43	28.90	35.98	35.58
Understorey	22.1129	143.0500	7.80	9.30	3.38	42.64	24.48	26.82	42.31	24.25	26.47
Understorey	156.1208	265.2251	7.80	9.20	3.11	12.98	25.32	23.70	11.94	24.20	22.67

Understorey	160.4561	329.0137	7.80	9.00	2.59	41.02	32.54	27.08	39.58	30.99	25.79
Understorey	151.3610	321.0929	7.80	8.80	2.09	43.15	38.71	32.11	42.51	37.76	31.28
Understorey	31.3103	129.5847	7.80	8.70	1.85	22.43	25.07	36.51	22.43	24.93	36.31
Understorey	86.7397	299.6568	7.80	8.70	1.85	17.24	30.79	32.50	16.04	29.65	31.66
Understorey	113.0665	281.4753	7.80	8.70	1.85	30.58	33.05	24.27	28.05	31.35	22.78
Understorey	29.2898	191.0996	7.80	8.60	1.62	50.18	50.89	31.82	50.04	50.82	31.67
Understorey	49.8718	209.6659	7.80	8.50	1.39	29.38	22.14	16.95	28.99	21.67	16.48
Understorey	135.8738	321.7871	7.80	8.40	1.17	36.30	21.81	29.29	35.86	21.22	28.77
Understorey	60.9653	217.8481	7.80	8.20	0.75	48.08	31.63	34.76	47.95	31.46	34.52
Understorey	90.7557	134.4552	7.80	8.20	0.75	35.31	27.48	31.83	35.15	27.33	31.71
Understorey	56.9590	232.7287	7.80	8.20	0.75	31.33	33.69	25.17	31.08	33.39	24.88
Understorey	73.6476	105.1982	7.80	8.10	0.56	2.81	24.59	26.08	2.65	24.33	25.74
Understorey	148.5323	316.0846	7.80	8.00	0.36	51.70	33.36	34.75	51.55	32.98	34.29
Understorey	139.3736	289.5808	7.80	8.00	0.36	18.18	25.59	30.18	18.06	25.46	29.86
Understorey	110.9633	289.2364	7.80	8.00	0.36	10.67	25.19	28.60	9.97	24.56	27.92
Understorey	98.8584	64.2990	7.80	7.90	0.18	23.41	19.75	18.85	23.00	19.44	18.68
Understorey	90.9420	245.0527	7.70	11.80	12.74	37.39	34.86	32.88	36.46	33.59	31.36
Understorey	79.1042	48.7763	7.70	10.00	5.75	33.78	28.22	26.42	33.20	27.60	25.63
Understorey	34.5162	129.4375	7.70	9.60	4.50	46.47	30.14	31.42	46.28	29.83	31.13
Understorey	43.2224	94.9575	7.70	9.50	4.20	11.32	22.41	51.84	11.32	22.41	51.84
Understorey	67.2553	64.0451	7.70	9.30	3.63	16.43	46.35	40.82	16.43	46.26	40.44
Understorey	104.6396	68.7701	7.70	9.00	2.82	19.58	20.52	15.79	18.49	20.04	15.27
Understorey	114.5964	120.3240	7.70	8.90	2.56	34.41	50.10	30.21	34.06	49.94	29.90
Understorey	108.2509	118.8311	7.70	8.50	1.60	59.24	42.07	31.91	59.07	41.85	31.71
Understorey	140.0915	119.4314	7.70	8.40	1.37	6.87	9.10	16.83	6.72	8.96	16.67
Understorey	95.8408	83.4877	7.70	8.40	1.37	9.14	15.72	15.60	9.14	15.58	15.15
Understorey	76.1825	132.9121	7.70	8.20	0.95	40.15	41.00	44.86	40.15	40.85	44.74
Understorey	95.6221	115.6178	7.70	8.10	0.75	5.14	29.35	31.12	4.66	29.07	30.92
Understorey	30.1046	31.4174	7.70	8.00	0.55	46.69	28.50	29.37	46.40	28.25	29.23
Understorey	153.6160	276.4610	7.70	8.00	0.55	4.48	18.80	24.51	3.79	18.35	23.89
Understorey	86.7457	51.1310	7.70	8.00	0.55	25.77	32.70	22.95	25.77	32.63	22.77
Understorey	158.6356	289.6531	7.70	7.80	0.18	32.82	21.76	25.12	32.53	21.58	24.81
Understorey	117.3601	285.4225	7.70	7.80	0.18	17.99	20.46	24.20	17.60	20.10	23.75
Understorey	159.5272	292.1467	7.60	12.70	17.45	32.41	22.22	25.36	30.25	18.72	21.68
Understorey	149.1007	117.9095	7.60	10.80	8.90	24.63	12.72	37.59	23.85	12.19	36.77
Understorey	125.7326	136.9776	7.60	10.60	8.15	19.57	23.76	31.22	19.31	23.31	30.62
Understorey	25.1082	134.8506	7.60	10.50	7.78	2.32	21.73	33.73	1.04	20.53	32.52
Understorey	162.6570	104.4973	7.60	10.00	6.03	51.53	38.86	40.75	51.53	38.65	40.45
Understorey	172.4336	97.7866	7.60	10.00	6.03	34.99	28.78	36.49	34.77	28.38	36.09
Understorey	23.6132	158.9993	7.60	9.50	4.46	24.39	31.85	40.30	24.06	31.20	39.77
Understorey	85.9213	92.7092	7.60	9.40	4.16	12.79	11.70	23.67	11.85	10.78	22.96
Understorey	20.5003	126.2671	7.60	9.10	3.32	21.63	16.33	16.74	21.30	15.97	16.27
Understorey	120.3823	227.2602	7.60	9.00	3.05	49.60	36.45	37.97	49.42	35.80	37.57
Understorey	103.0240	24.6287	7.60	8.90	2.79	47.41	39.88	36.92	46.74	39.07	36.24
Understorey	77.5359	33.8436	7.60	8.80	2.53	4.76	27.80	22.46	4.57	27.72	22.09
Understorey	96.9863	92.2297	7.60	8.60	2.04	11.69	16.06	18.42	11.35	15.64	17.91
Understorey	43.5799	221.1830	7.60	8.40	1.58	1.51	4.09	13.40	1.23	3.80	13.05
Understorey	62.6408	125.8889	7.60	8.30	1.36	63.74	42.22	42.15	63.43	42.08	41.99
Understorey	50.9826	246.8485	7.60	8.00	0.74	37.07	24.55	36.05	36.66	24.24	35.85
Understorey	32.8428	24.5769	7.60	7.90	0.54	54.03	39.70	33.99	53.62	39.52	33.89
Understorey	103.0690	303.2911	7.60	7.90	0.54	30.57	19.55	17.53	30.29	19.31	17.23

Understorey	96.5149	282.3001	7.60	7.80	0.35	60.50	43.41	32.90	59.67	42.86	32.15
Understorey	51.8320	23.4478	7.50	11.50	12.12	54.39	44.29	45.48	53.30	42.65	43.66
Understorey	85.0936	273.3240	7.50	10.50	8.08	28.70	24.37	37.30	26.38	22.13	35.48
Understorey	169.4479	318.9790	7.50	10.30	7.35	16.46	23.10	22.21	13.42	20.97	20.41
Understorey	76.6069	42.4455	7.50	10.00	6.31	4.12	24.24	31.68	3.11	23.54	31.07
Understorey	116.5703	252.3500	7.50	9.90	5.98	44.29	20.59	20.43	43.73	19.93	19.25
Understorey	157.5518	284.3258	7.50	9.50	4.71	8.21	26.11	24.08	6.41	24.38	22.55
Understorey	80.4459	260.2422	7.50	9.40	4.41	28.70	23.99	34.89	27.89	23.07	33.84
Understorey	96.7829	308.0695	7.50	9.40	4.41	20.04	32.62	26.50	19.11	31.70	25.67
Understorey	83.3374	26.0237	7.50	9.30	4.12	7.51	26.61	34.40	6.40	25.64	33.38
Understorey	151.3040	35.6202	7.50	9.30	4.12	27.61	22.95	25.01	26.90	22.32	24.46
Understorey	37.5000	48.1000	7.50	9.20	3.83	33.90	32.61	40.72	33.90	32.52	40.54
Understorey	175.0000	127.0000	7.50	9.10	3.55	19.25	23.23	32.95	19.05	23.06	32.81
Understorey	102.5901	112.7266	7.50	9.10	3.55	49.68	29.99	31.20	48.98	28.80	30.44
Understorey	128.7856	267.9064	7.50	9.10	3.55	28.67	27.92	27.25	27.41	26.73	26.12
Understorey	138.9760	295.8709	7.50	9.10	3.55	44.35	30.25	26.32	43.73	29.59	25.65
Understorey	24.4596	370.8742	7.50	9.00	3.28	17.07	23.44	37.85	16.24	22.85	37.41
Understorey	30.9180	37.1337	7.50	8.90	3.02	37.05	27.16	37.19	36.89	26.81	36.91
Understorey	151.8894	319.5513	7.50	8.70	2.50	44.90	29.70	39.43	44.44	28.91	38.57
Understorey	116.0183	36.1585	7.50	8.70	2.50	65.52	53.28	34.18	65.52	53.21	34.05
Understorey	80.7053	37.2352	7.50	8.70	2.50	4.75	15.09	29.19	4.75	15.02	28.98
Understorey	164.6595	346.8540	7.50	8.70	2.50	19.35	21.91	26.27	19.00	21.58	25.83
Understorey	112.5000	281.6000	7.50	8.50	2.02	30.59	33.05	24.14	28.61	31.69	22.90
Understorey	113.8221	284.4075	7.50	8.40	1.79	19.19	26.87	25.37	17.85	25.90	24.34
Understorey	168.7909	286.8315	7.50	8.20	1.34	21.82	15.77	26.06	21.39	14.95	25.49
Understorey	131.1819	276.6564	7.50	8.20	1.34	29.73	19.85	24.83	29.29	19.20	24.06
Understorey	96.2558	68.2953	7.50	8.00	0.93	16.18	13.96	19.99	15.76	13.59	19.74
Understorey	148.8897	116.8721	7.50	7.90	0.73	24.76	16.84	39.63	24.76	16.77	39.59
Understorey	101.1151	21.5818	7.50	7.80	0.54	39.07	29.96	31.35	38.92	29.83	31.25
Understorey	132.0677	276.3738	7.50	7.80	0.54	30.22	16.35	24.41	29.94	16.03	24.03
Understorey	90.0869	198.7053	7.50	7.70	0.35	25.47	45.93	37.43	25.34	45.87	37.40
Understorey	176.0403	266.6723	7.50	7.70	0.35	13.62	41.22	32.92	13.62	41.11	32.70
Understorey	64.1144	123.9871	7.50	7.60	0.17	63.77	42.52	41.19	63.77	42.52	41.19
Understorey	76.7202	76.2045	7.50	7.60	0.17	68.91	33.62	30.79	68.91	33.50	30.65
Understorey	98.7887	239.6943	7.40	9.70	5.60	18.84	30.17	21.35	17.71	28.77	20.35
Understorey	87.6745	234.9985	7.40	9.50	4.97	50.07	33.94	37.28	49.67	33.52	36.65
Understorey	63.9760	358.3709	7.40	9.40	4.67	13.90	22.49	33.15	13.49	22.18	32.79
Understorey	140.2952	35.4320	7.40	9.30	4.37	24.09	22.07	25.46	23.71	21.58	25.05
Understorey	73.0597	225.9239	7.40	9.20	4.08	33.80	48.34	38.76	33.67	48.07	38.31
Understorey	95.4696	263.1972	7.40	9.20	4.08	10.47	23.93	28.37	9.38	22.60	27.00
Understorey	133.0566	120.2802	7.40	8.80	2.98	8.22	9.07	16.59	8.04	8.99	16.38
Understorey	31.4367	150.9046	7.40	8.50	2.23	50.42	39.92	29.24	50.42	39.84	29.03
Understorey	119.8540	268.6708	7.40	8.50	2.23	21.45	26.88	27.04	20.41	25.40	25.87
Understorey	26.8840	163.8192	7.40	8.40	2.00	11.62	39.09	44.78	11.47	39.02	44.58
Understorey	47.2124	342.6642	7.40	8.40	2.00	38.79	37.02	31.71	38.37	36.52	31.27
Understorey	174.0582	329.2068	7.40	8.30	1.77	28.23	19.89	25.28	27.80	19.08	24.47
Understorey	146.3325	109.8095	7.40	8.10	1.33	23.31	33.26	44.33	23.00	33.12	44.26
Understorey	87.6291	366.1977	7.40	8.00	1.12	46.35	41.48	35.13	46.35	41.30	35.00
Understorey	57.1009	248.8638	7.40	8.00	1.12	28.24	34.26	33.01	27.83	33.89	32.80
Understorey	144.9478	104.3000	7.40	7.80	0.72	46.94	39.76	41.83	46.94	39.76	41.83
Understorey	39.6748	365.4934	7.40	7.80	0.72	25.51	36.03	31.88	25.22	35.79	31.60

Understorey	94.0961	234.4202	7.40	7.80	0.72	12.17	29.49	28.75	12.04	29.19	28.51
Understorey	82.5718	95.3408	7.40	7.60	0.35	9.80	19.04	22.80	9.53	18.92	22.73
Understorey	122.8046	311.4444	7.40	7.60	0.35	31.60	20.39	22.00	31.60	20.27	21.83
Understorey	28.1124	193.8814	7.40	7.50	0.17	55.27	42.10	30.92	55.27	42.10	30.89
Understorey	37.0498	214.4085	7.40	7.50	0.17	3.95	4.21	23.89	3.56	3.98	23.73
Understorey	87.6187	256.7990	7.30	11.10	11.09	41.58	33.64	21.59	39.87	31.80	19.89
Understorey	83.0548	275.3903	7.30	9.20	4.33	28.61	30.01	39.36	27.20	28.49	37.98
Understorey	154.5446	311.5372	7.30	9.00	3.76	32.56	43.57	31.05	32.03	42.46	29.97
Understorey	107.5683	301.8870	7.30	9.00	3.76	30.99	20.40	19.45	30.01	19.76	18.69
Understorey	42.0220	359.6975	7.30	8.90	3.48	16.89	32.81	32.88	15.80	31.88	32.31
Understorey	97.5684	230.1301	7.30	8.70	2.95	26.57	26.33	30.60	26.15	25.78	30.15
Understorey	143.0242	280.9240	7.30	8.50	2.45	5.93	15.75	18.42	4.98	14.87	17.59
Understorey	94.9478	29.3000	7.30	8.40	2.21	28.28	18.31	29.00	27.66	17.89	28.64
Understorey	115.1884	283.2742	7.30	8.40	2.21	32.23	26.97	25.33	30.88	26.05	24.29
Understorey	103.7532	63.0945	7.30	8.20	1.75	29.07	18.54	19.82	28.33	18.22	19.56
Understorey	84.6167	72.1167	7.30	8.10	1.53	30.94	44.07	36.81	30.52	43.81	36.49
Understorey	33.3739	119.1609	7.30	8.00	1.31	46.16	45.93	47.73	46.16	45.87	47.66
Understorey	53.0559	26.4905	7.30	8.00	1.31	51.82	43.09	44.59	51.82	42.96	44.39
Understorey	67.7000	109.0067	7.30	8.00	1.31	1.20	40.17	36.44	1.20	40.17	36.33
Understorey	63.6110	241.1340	7.30	8.00	1.31	41.08	44.27	35.51	40.95	43.96	35.23
Understorey	117.0604	276.1370	7.30	8.00	1.31	28.51	16.95	32.78	27.52	15.84	31.89
Understorey	99.2713	275.8231	7.30	7.90	1.10	39.19	37.01	31.92	38.31	35.88	31.18
Understorey	83.0039	296.7075	7.30	7.70	0.71	22.63	33.53	37.97	22.09	33.06	37.64
Understorey	23.2480	135.7480	7.30	7.70	0.71	1.92	20.12	30.30	1.92	20.12	30.30
Understorey	91.2675	25.4960	7.30	7.70	0.71	23.14	18.09	23.69	22.99	18.02	23.65
Understorey	40.3842	29.8001	7.30	7.60	0.52	28.32	38.59	36.24	28.18	38.48	36.17
Understorey	132.1367	335.3840	7.30	7.50	0.34	27.68	39.77	29.54	27.68	39.71	29.51
Understorey	108.6776	297.8416	7.30	7.50	0.34	16.46	17.60	22.66	16.33	17.48	22.43
Understorey	139.7233	361.9957	7.30	7.40	0.17	52.93	39.67	39.47	52.93	39.67	39.47
Understorey	59.0860	117.9594	7.25	8.20	1.85	47.96	63.86	60.15	47.82	63.72	60.04
Understorey	100.9386	268.4261	7.20	9.60	5.82	18.93	32.14	37.49	16.41	30.12	35.43
Understorey	174.6876	298.4737	7.20	9.50	5.50	16.49	28.32	26.78	14.99	27.16	25.49
Understorey	113.4370	301.8794	7.20	9.50	5.50	21.94	20.36	21.86	21.05	19.48	21.01
Understorey	96.8458	275.3634	7.20	9.10	4.28	46.13	36.66	31.52	45.12	34.80	29.71
Understorey	70.7693	290.5098	7.20	8.90	3.72	36.79	38.69	44.83	36.44	38.00	44.10
Understorey	45.7695	362.0322	7.20	8.80	3.45	25.54	24.26	33.15	24.77	23.74	32.65
Understorey	98.4408	229.4204	7.20	8.80	3.45	26.21	28.83	28.38	25.72	28.11	27.83
Understorey	159.3902	26.9960	7.20	8.70	3.18	49.54	42.85	35.41	49.54	42.77	35.24
Understorey	111.4143	276.4015	7.20	8.50	2.67	27.89	32.25	27.25	25.60	30.61	25.99
Understorey	80.3389	289.7662	7.20	8.40	2.42	8.17	45.67	34.01	6.97	44.81	33.23
Understorey	111.2452	122.2753	7.20	8.30	2.18	59.22	41.97	37.22	59.22	41.97	37.13
Understorey	80.3329	272.1208	7.20	8.20	1.95	19.01	28.03	37.83	18.24	27.16	37.11
Understorey	76.1846	51.9715	7.20	8.20	1.95	59.26	32.96	25.57	59.26	32.89	25.46
Understorey	178.8627	361.8882	7.20	8.10	1.73	27.93	36.74	32.20	27.93	36.68	32.10
Understorey	21.1881	162.9553	7.20	7.70	0.89	14.99	30.80	41.21	14.99	30.80	41.18
Understorey	125.3664	335.4936	7.20	7.60	0.70	34.90	31.33	29.30	34.47	31.14	29.12
Understorey	110.3521	88.4968	7.20	7.50	0.52	48.52	31.07	23.48	48.52	31.01	23.42
Understorey	21.7710	367.6390	7.20	7.40	0.34	16.25	34.92	43.15	16.25	34.92	43.11
Understorey	78.9779	360.3627	7.20	7.40	0.34	48.45	25.69	22.33	48.32	25.58	22.23
Understorey	36.1016	23.6016	7.20	7.30	0.16	40.66	32.82	38.29	40.53	32.77	38.26
Understorey	141.9739	56.3894	7.20	7.30	0.16	35.47	45.38	37.31	35.47	45.38	37.31

Understorey	97.8521	63.4968	7.20	7.30	0.16	23.74	19.67	22.70	23.61	19.55	22.63
Understorey	176.9881	94.2117	7.10	11.00	11.33	17.56	25.84	31.06	16.35	24.78	30.16
Understorey	23.1837	230.6806	7.10	10.90	10.91	36.82	40.52	31.14	36.49	40.07	30.38
Understorey	136.1823	104.0700	7.10	10.50	9.31	54.79	44.31	38.52	53.51	43.26	37.74
Understorey	136.6533	136.2535	7.10	10.50	9.31	22.27	19.17	24.15	22.02	18.85	23.70
Understorey	78.6071	22.1477	7.10	10.20	8.17	34.97	34.10	39.97	33.72	32.96	38.45
Understorey	34.7749	154.3521	7.10	9.60	6.09	67.52	45.10	33.25	67.34	44.84	32.97
Understorey	91.8762	47.8097	7.10	9.40	5.44	1.92	17.41	28.97	1.37	17.09	28.45
Understorey	135.3945	35.3945	7.10	9.30	5.13	22.91	27.87	27.14	22.50	27.54	26.77
Understorey	165.7076	301.6344	7.10	9.30	5.13	6.29	26.97	22.14	5.12	25.92	20.78
Understorey	143.7098	265.9421	7.10	9.20	4.83	6.86	28.10	20.90	5.82	26.69	19.66
Understorey	42.9873	360.1064	7.10	8.60	3.15	28.60	32.01	33.28	27.89	31.49	32.90
Understorey	37.9580	347.9900	7.10	8.60	3.15	34.24	27.60	29.34	33.67	27.05	28.81
Understorey	32.6969	169.9328	7.10	8.50	2.89	26.68	43.96	38.73	26.51	43.81	38.52
Understorey	61.3511	79.4713	7.10	8.40	2.64	20.73	35.35	33.14	20.73	35.35	33.04
Understorey	152.4412	307.5133	7.10	8.40	2.64	38.28	51.14	31.69	38.12	50.74	31.09
Understorey	25.6779	340.8317	7.10	8.20	2.16	28.30	33.17	36.54	28.13	32.95	36.34
Understorey	113.0094	215.2652	7.10	8.20	2.16	65.09	47.05	36.17	64.94	46.85	35.98
Understorey	140.7150	296.3331	7.10	8.20	2.16	45.42	38.55	29.95	45.14	38.30	29.62
Understorey	157.3474	43.0367	7.10	8.10	1.93	9.36	20.96	29.00	9.05	20.75	28.88
Understorey	171.9045	284.4045	7.10	8.10	1.93	22.10	15.61	24.92	21.71	14.87	24.35
Understorey	160.3398	46.0234	7.10	8.00	1.71	14.66	24.25	28.72	14.66	24.11	28.65
Understorey	82.1479	89.4153	7.10	8.00	1.71	9.62	35.02	25.49	9.06	34.71	25.28
Understorey	146.6791	41.2086	7.10	8.00	1.71	12.92	27.66	24.28	12.77	27.52	24.17
Understorey	126.5800	97.4757	7.10	7.90	1.49	58.23	55.04	46.83	58.23	55.04	46.69
Understorey	119.6393	240.3845	7.10	7.80	1.28	42.14	23.34	30.05	42.14	23.34	29.94
Understorey	41.1135	367.6607	7.10	7.70	1.08	11.13	33.01	31.15	10.98	32.88	30.94
Understorey	107.7461	277.3682	7.10	7.60	0.88	56.04	32.13	24.90	55.38	31.72	24.51
Understorey	93.4532	289.5615	7.10	7.40	0.51	29.88	33.64	32.27	29.75	33.29	32.05
Understorey	90.6297	188.8285	7.10	7.30	0.33	16.68	30.64	38.51	16.68	30.58	38.48
Understorey	115.5103	282.8421	7.10	7.30	0.33	32.12	26.88	25.50	31.73	26.65	25.27
Understorey	100.1630	177.4614	7.10	7.20	0.16	46.62	42.35	44.05	46.62	42.35	44.05
Understorey	133.7296	309.3613	7.10	7.20	0.16	32.43	43.27	33.86	32.43	43.27	33.79
Understorey	63.2372	200.5162	7.10	7.20	0.16	37.68	31.84	30.25	37.68	31.84	30.22
Understorey	40.5686	206.2916	7.10	7.20	0.16	6.84	16.42	25.08	6.72	16.31	25.02
Understorey	114.2835	289.1059	7.10	7.20	0.16	18.93	18.40	20.35	18.93	18.29	20.29
Understorey	96.7231	98.6886	7.00	9.90	7.39	13.81	25.51	25.44	13.09	24.50	24.35
Understorey	97.7014	123.6329	7.00	9.40	5.71	10.03	21.13	35.77	9.04	20.07	34.97
Understorey	40.0359	346.3436	7.00	9.20	5.08	34.61	38.39	28.26	33.49	37.53	27.45
Understorey	131.7525	152.1940	7.00	9.00	4.49	57.47	52.30	36.59	57.30	52.23	36.50
Understorey	132.8240	27.0964	7.00	8.90	4.20	13.32	25.53	35.28	13.32	25.53	35.11
Understorey	172.4937	319.4976	7.00	8.90	4.20	10.36	19.68	25.49	7.98	18.40	24.69
Understorey	176.0023	123.9562	7.00	8.80	3.92	0.39	23.59	31.85	0.21	23.51	31.76
Understorey	142.6173	168.3867	7.00	8.70	3.64	35.72	35.09	39.11	35.72	35.09	39.07
Understorey	108.5229	274.2307	7.00	8.70	3.64	55.43	30.72	27.07	52.62	28.69	25.63
Understorey	122.0766	288.1697	7.00	8.70	3.64	19.60	18.41	24.68	18.70	17.50	23.65
Understorey	175.7060	308.0692	7.00	8.70	3.64	26.71	23.72	21.80	25.79	22.46	20.83
Understorey	94.5560	36.7432	7.00	8.40	2.86	3.19	13.71	26.72	3.01	13.50	26.44
Understorey	82.8020	292.3753	7.00	8.30	2.61	9.02	44.56	35.61	7.82	43.72	34.92
Understorey	127.6461	159.2281	7.00	8.20	2.37	29.27	39.53	41.68	29.27	39.53	41.68
Understorey	32.1817	349.6921	7.00	7.90	1.69	27.20	19.91	34.78	27.07	19.66	34.60

Understorey	106.3638	69.8207	7.00	7.90	1.69	21.80	20.19	15.31	21.52	20.00	15.17
Understorey	149.1572	46.9398	7.00	7.70	1.26	12.93	27.43	33.63	12.93	27.37	33.60
Understorey	118.3177	335.4954	7.00	7.50	0.87	25.77	32.09	37.93	25.77	32.03	37.90
Understorey	20.4230	114.1841	7.00	7.30	0.50	10.81	26.83	34.68	10.81	26.83	34.68
Understorey	153.1696	331.7973	7.00	7.30	0.50	30.91	33.77	30.10	30.65	33.65	30.00
Understorey	44.9954	212.7617	7.00	7.30	0.50	8.97	14.74	14.33	8.71	14.57	14.17
Understorey	76.0348	91.9821	7.00	7.20	0.33	25.70	31.65	25.59	25.70	31.65	25.59
Understorey	99.4395	68.5835	7.00	7.20	0.33	14.53	20.70	18.15	14.53	20.70	18.15
Overstorey	61.4552	130.7113	115.50	116.90	36.94	13.06	30.02	27.00	0.00	0.00	0.00
Overstorey	56.0876	57.9335	107.50	108.00	12.15	53.59	29.12	35.33	0.00	0.00	0.00
Overstorey	78.8891	116.3891	102.30	106.50	102.35	17.98	20.23	17.43	0.00	0.00	0.00
Overstorey	110.1420	35.5024	98.50	99.80	29.30	38.32	33.01	33.04	0.00	0.00	0.00
Overstorey	51.7672	33.3143	98.00	100.50	57.08	18.85	35.45	36.87	0.00	0.00	0.00
Overstorey	145.6978	301.2984	97.20	104.10	166.52	12.46	30.65	25.91	0.00	0.00	0.00
Overstorey	73.2125	78.8990	97.00	100.60	82.72	28.38	27.70	29.18	0.00	0.00	6.41
Overstorey	66.1554	34.5225	97.00	98.50	33.41	10.31	40.86	37.61	0.00	11.22	6.31
Overstorey	29.6396	81.2701	96.50	103.90	178.67	4.14	23.47	30.52	0.00	0.00	0.00
Overstorey	165.0961	113.5489	96.50	102.50	142.04	37.76	36.22	26.97	0.00	0.00	0.00
Overstorey	42.5328	127.6760	96.20	99.20	67.79	41.44	38.05	43.10	0.00	0.00	8.54
Overstorey	79.3331	218.2502	96.10	101.70	131.31	10.41	33.69	34.05	0.00	0.00	0.00
Overstorey	66.4394	236.4408	94.70	95.20	10.71	26.97	22.22	30.09	0.00	0.00	0.00
Overstorey	77.4979	60.4044	94.10	101.30	169.48	34.06	33.72	32.35	0.00	0.00	0.00
Overstorey	178.4500	55.9756	93.40	95.00	34.39	7.48	13.83	28.19	0.00	0.00	8.95
Overstorey	27.0572	102.8316	92.20	94.80	56.07	37.60	22.36	25.05	0.00	0.00	0.00
Overstorey	171.9870	200.4972	91.00	92.40	29.25	42.66	59.17	41.70	0.00	0.00	0.00
Overstorey	126.1080	348.0419	89.90	91.50	33.14	32.10	38.10	28.91	0.00	0.00	0.00
Overstorey	67.7597	251.6080	89.70	92.80	65.63	11.87	21.75	31.11	0.00	0.00	5.66
Overstorey	124.1825	54.7200	88.70	92.30	76.02	27.92	23.31	33.68	0.00	0.00	0.00
Overstorey	33.6300	195.5476	88.40	91.20	58.18	6.42	39.96	25.76	0.00	0.00	0.00
Overstorey	59.1000	212.5000	88.30	92.10	80.15	20.06	23.02	18.89	0.00	0.00	0.00
Overstorey	26.5045	151.9966	88.30	90.20	38.86	22.18	24.77	27.81	0.00	0.00	0.00
Overstorey	156.3806	87.7976	88.10	88.50	7.96	18.64	27.65	25.10	0.00	0.00	0.00
Overstorey	101.0245	280.8104	87.50	91.30	79.47	20.41	28.10	27.97	0.00	0.00	0.00
Overstorey	40.1140	113.1760	87.50	89.80	46.94	45.56	55.37	51.37	0.00	10.93	11.77
Overstorey	135.9413	258.2449	87.40	89.80	49.01	15.85	7.87	19.79	0.00	0.00	5.32
Overstorey	39.9727	56.4417	86.70	88.50	36.11	55.45	31.10	33.03	0.00	0.00	7.29
Overstorey	177.2683	214.3215	86.50	90.30	78.62	17.62	47.18	47.22	0.00	9.49	11.19
Overstorey	90.8992	285.4616	86.50	90.10	74.24	18.36	34.33	28.59	0.00	9.26	5.21
Overstorey	85.7702	244.2300	86.50	89.20	54.86	1.88	34.51	29.05	0.00	0.00	5.38
Overstorey	119.6697	85.2394	86.50	89.00	50.63	13.93	29.52	23.70	0.00	0.00	0.00
Overstorey	131.8200	157.3135	86.40	87.70	25.77	37.91	42.09	40.79	0.00	0.00	0.00
Overstorey	117.8509	129.0322	86.10	91.00	102.75	16.47	18.78	25.49	0.00	0.00	0.00
Overstorey	139.0307	153.6955	86.00	87.30	25.66	60.45	44.11	37.03	19.23	8.55	4.81
Overstorey	134.0502	274.0838	85.90	92.30	137.15	8.89	6.22	18.94	0.00	0.00	0.00
Overstorey	26.5455	146.2648	85.90	88.00	41.96	22.44	16.32	22.84	20.34	9.04	5.08
Overstorey	172.3364	26.1207	85.50	89.00	71.27	49.97	40.59	24.41	21.62	19.12	10.75
Overstorey	44.4710	168.3494	85.50	86.30	15.56	33.90	37.01	30.17	0.00	0.00	0.00
Overstorey	109.6162	110.3121	85.20	88.20	60.38	43.95	31.98	28.84	0.00	0.00	0.00
Overstorey	61.0983	375.1937	85.00	86.00	19.41	36.30	22.24	21.18	0.00	0.00	0.00
Overstorey	55.0320	40.7678	85.00	85.70	13.52	36.71	34.51	39.42	25.25	22.00	19.67
Overstorey	69.5928	95.3773	84.80	90.10	110.31	8.90	12.77	23.14	0.00	0.00	6.33

Overstorey	76.8126	175.8452	84.50	87.70	64.12	46.04	32.20	36.39	0.00	0.00	0.00
Overstorey	25.0000	236.2000	84.50	87.20	53.65	24.12	21.46	29.64	0.00	0.00	0.00
Overstorey	39.0837	158.1475	84.50	86.00	29.20	31.64	36.17	28.87	0.00	17.31	14.58
Overstorey	52.0490	111.6207	84.20	86.00	35.10	48.92	46.69	37.99	0.00	8.96	11.19
Overstorey	152.3470	62.0740	84.00	87.40	67.98	29.33	34.83	37.56	0.00	0.00	0.00
Overstorey	166.0739	125.9576	84.00	87.30	65.87	18.40	24.78	31.59	0.00	11.67	6.57
Overstorey	166.5657	151.3999	84.00	86.40	47.18	38.50	36.43	30.38	0.00	0.00	0.00
Overstorey	40.8328	183.6823	84.00	86.30	45.13	8.87	31.77	29.61	0.00	9.24	5.20
Overstorey	74.0737	20.0160	83.70	85.40	32.90	26.39	40.00	31.37	0.00	11.20	12.36
Overstorey	164.0455	33.7648	83.40	87.60	84.54	4.20	23.59	32.01	0.00	8.80	15.70
Overstorey	160.6593	200.9326	83.20	85.20	38.69	40.25	35.78	48.53	0.00	9.49	5.34
Overstorey	30.5285	252.5780	83.10	84.20	20.92	32.04	29.59	32.43	0.00	0.00	4.75
Overstorey	65.8807	175.9059	83.00	83.50	9.40	31.35	47.53	31.52	0.00	8.55	4.81
Overstorey	130.8376	357.7468	82.80	87.60	96.94	1.68	35.14	37.58	0.00	9.30	5.23
Overstorey	171.5333	72.5325	82.80	84.60	34.53	66.03	46.70	50.47	35.82	15.92	19.19
Overstorey	22.9320	202.7952	82.70	83.40	13.16	16.66	28.17	25.72	0.00	9.24	5.20
Overstorey	81.2353	367.8255	82.50	86.80	85.83	24.65	21.63	23.67	0.00	0.00	5.04
Overstorey	160.7553	179.3454	82.40	84.70	44.31	47.49	41.38	37.73	0.00	0.00	0.00
Overstorey	57.1659	238.8929	82.00	83.60	30.30	46.25	27.28	25.00	22.66	10.07	11.05
Overstorey	40.8065	160.1763	81.90	84.90	58.16	50.46	27.34	32.68	37.11	16.49	19.20
Overstorey	118.8912	317.1435	81.80	85.00	62.18	2.58	10.12	22.17	0.00	0.00	0.00
Overstorey	163.1805	107.0673	81.80	84.80	58.10	46.05	35.82	34.14	26.26	11.67	11.33
Overstorey	160.1881	152.5402	81.80	83.40	30.23	52.26	35.40	31.35	18.66	8.29	4.67
Overstorey	46.6881	61.3463	81.70	83.80	39.98	70.25	49.11	37.74	48.74	21.66	12.18
Overstorey	113.4413	223.2589	81.30	83.00	31.99	33.92	29.42	30.28	17.26	7.67	4.32
Overstorey	33.6404	99.3925	81.00	83.30	43.58	33.49	23.30	37.40	22.47	9.99	17.40
Overstorey	93.8948	140.3472	80.80	81.30	9.15	25.95	32.53	40.14	0.00	0.00	0.00
Overstorey	118.5195	217.9200	80.70	83.10	45.40	30.13	31.11	31.13	0.00	0.00	0.00
Overstorey	46.8695	77.5105	80.10	82.50	45.08	9.55	14.47	25.71	0.00	0.00	11.14
Overstorey	20.9961	260.8745	79.50	82.00	46.70	25.54	39.50	37.14	0.00	16.44	19.69
Overstorey	33.0172	377.1482	79.30	84.20	95.27	19.75	16.77	21.72	0.00	0.00	0.00
Overstorey	36.4195	30.3250	78.90	82.20	62.09	24.90	26.26	27.26	0.00	7.69	10.64
Overstorey	155.3123	295.3759	78.70	80.80	38.57	8.77	25.20	19.77	0.00	12.04	6.77
Overstorey	66.5558	157.4699	78.70	80.60	34.77	15.48	23.59	34.19	0.00	0.00	4.36
Overstorey	169.5106	253.4193	78.50	81.80	61.79	38.44	30.72	35.02	0.00	0.00	0.00
Overstorey	100.6228	274.3837	78.50	79.60	19.78	24.89	31.42	27.86	20.84	18.28	10.28
Overstorey	52.9952	121.1988	78.40	80.50	38.43	43.09	57.51	55.89	18.49	34.33	28.48
Overstorey	119.4340	97.7801	78.00	81.00	55.54	35.48	43.25	38.34	0.00	8.80	9.81
Overstorey	69.5388	291.8983	78.00	80.50	45.86	18.36	28.12	36.56	0.00	0.00	0.00
Overstorey	123.4866	144.6348	77.70	78.20	8.80	1.59	18.32	37.37	0.00	0.00	14.75
Overstorey	94.0778	252.3941	77.40	80.30	53.20	10.23	21.42	21.00	0.00	8.84	4.97
Overstorey	70.0548	152.3097	77.40	78.70	23.15	16.24	30.19	20.23	16.24	7.22	4.06
Overstorey	50.6459	280.2605	77.20	80.80	66.73	22.11	33.08	27.91	0.00	0.00	0.00
Overstorey	32.5237	60.3554	77.20	78.70	26.74	30.83	31.76	32.98	19.58	16.50	9.28
Overstorey	96.3346	26.7173	77.00	82.00	94.81	12.19	11.53	26.92	0.00	0.00	6.22
Overstorey	106.5114	217.9637	77.00	79.10	37.77	20.48	34.40	36.47	17.22	15.33	8.62
Overstorey	156.3792	320.9655	76.80	80.70	72.33	16.21	27.92	25.42	0.00	0.00	0.00
Overstorey	175.5755	138.0557	76.80	78.40	28.44	13.95	20.12	28.40	0.00	7.56	13.68
Overstorey	105.8505	308.3554	76.70	81.80	96.53	9.24	9.77	20.09	0.00	0.00	4.52
Overstorey	116.5924	175.9448	76.70	78.00	22.94	33.03	40.03	35.41	0.00	0.00	0.00
Overstorey	71.2912	122.6131	76.50	81.30	90.15	43.54	44.04	33.93	28.35	27.79	15.63

Overstorey	70.6888	200.4292	76.50	80.60	76.04	23.49	26.39	34.32	0.00	0.00	11.77
Overstorey	133.6383	68.1098	76.50	79.00	45.02	23.36	17.64	33.29	0.00	0.00	10.10
Overstorey	110.9849	63.0757	76.40	81.40	94.13	7.03	14.33	20.29	0.00	0.00	5.32
Overstorey	109.5733	41.5636	76.40	80.00	66.08	36.28	36.20	35.39	24.90	11.07	15.75
Overstorey	128.8976	100.1361	76.40	78.10	30.12	44.08	43.63	42.87	16.40	7.29	9.05
Overstorey	172.6860	225.5338	76.20	78.00	31.87	51.67	57.07	35.58	0.00	19.84	11.16
Overstorey	106.6996	179.1864	76.20	76.40	3.43	25.37	42.25	39.06	0.00	6.76	3.80
Overstorey	42.4253	106.3041	76.10	77.50	24.57	30.24	53.38	47.62	20.16	24.89	27.65
Overstorey	81.8242	363.5199	76.00	80.70	87.59	32.34	19.56	19.16	18.84	8.37	9.75
Overstorey	69.5534	72.2082	76.00	79.10	56.10	37.84	29.19	40.34	25.30	22.65	20.03
Overstorey	122.2693	235.8499	76.00	77.30	22.74	27.10	19.25	26.64	0.00	0.00	8.62
Overstorey	78.5562	205.0787	75.80	79.40	65.59	34.36	35.81	31.78	16.24	18.71	10.52
Overstorey	80.2315	55.5128	75.70	77.10	24.44	43.64	21.58	26.15	25.65	11.40	10.32
Overstorey	35.4893	123.3620	75.60	79.80	77.19	39.46	32.12	34.19	24.60	19.89	15.24
Overstorey	159.1717	130.5109	75.40	77.30	33.36	22.31	22.22	29.47	19.05	8.47	15.17
Overstorey	102.8738	193.6629	75.30	75.50	3.39	16.44	40.79	39.24	0.00	6.49	7.26
Overstorey	58.3329	168.5542	75.20	79.20	72.88	0.86	38.28	37.28	0.00	23.24	22.38
Overstorey	117.1057	258.3089	75.00	77.50	44.18	11.89	14.16	18.96	0.00	0.00	5.04
Overstorey	74.9604	128.3388	74.90	75.90	17.14	35.02	47.24	35.36	16.52	35.13	19.76
Overstorey	124.9000	187.5000	74.50	74.70	3.36	24.47	41.22	38.64	0.00	13.14	7.39
Overstorey	140.4945	92.5836	74.20	77.00	49.26	49.22	45.90	38.37	0.00	6.78	8.71
Overstorey	80.7725	153.1997	74.10	76.60	43.67	29.11	47.02	31.88	0.00	14.10	12.06
Overstorey	70.3492	288.3943	74.10	76.60	43.67	25.08	33.53	31.49	16.20	7.20	4.05
Overstorey	56.6468	106.6468	74.00	75.30	22.16	37.11	27.33	35.36	18.49	14.89	22.54
Overstorey	159.0032	69.1620	74.00	75.00	16.94	50.11	41.44	40.09	19.10	16.44	14.14
Overstorey	91.5330	320.7689	74.00	74.70	11.79	10.70	25.36	35.93	0.00	0.00	7.70
Overstorey	163.7329	156.9921	73.90	76.50	45.39	53.68	32.90	35.81	36.05	16.02	9.01
Overstorey	61.5831	205.9019	73.90	76.00	36.31	34.05	32.14	26.30	21.21	23.08	16.92
Overstorey	95.9004	179.8500	73.80	76.00	38.06	17.41	29.45	29.19	0.00	6.49	8.45
Overstorey	25.0436	26.9867	73.70	75.70	34.42	31.50	28.71	28.49	0.00	7.51	8.55
Overstorey	89.4844	185.2089	73.70	74.20	8.35	17.80	14.87	35.74	14.44	6.42	19.08
Overstorey	151.1196	216.1493	73.50	75.60	36.12	18.22	24.39	35.95	0.00	10.78	10.60
Overstorey	90.5091	341.4932	73.50	75.50	34.33	43.39	54.00	36.33	0.00	0.00	0.00
Overstorey	102.9048	171.4399	73.50	74.30	13.41	23.75	39.97	42.93	14.59	19.66	11.06
Overstorey	114.5317	274.0223	73.50	74.00	8.33	15.08	17.70	29.12	0.00	7.04	18.25
Overstorey	112.5000	200.3000	73.50	73.90	6.65	35.19	39.16	46.21	0.00	6.33	15.28
Overstorey	94.2175	206.7175	73.50	73.80	4.98	21.40	22.07	40.69	0.00	0.00	21.29
Overstorey	25.8541	203.1876	73.40	76.40	52.44	20.30	22.54	27.73	17.39	16.97	9.55
Overstorey	69.1471	216.1846	73.40	76.10	46.92	15.06	41.38	32.37	0.00	27.92	19.77
Overstorey	135.3405	176.8233	73.30	75.80	43.22	26.88	32.46	43.29	0.00	0.00	8.61
Overstorey	147.6126	27.4278	73.30	75.50	37.81	28.31	13.43	27.88	16.16	7.18	18.96
Overstorey	85.0412	303.0699	73.30	75.00	28.93	15.64	18.75	28.00	0.00	0.00	9.12
Overstorey	143.2430	315.9508	73.20	76.20	52.31	32.63	37.93	29.41	0.00	19.28	10.84
Overstorey	39.2176	331.8891	73.00	75.30	39.46	16.15	20.98	24.53	0.00	0.00	0.00
Overstorey	166.7374	329.3880	73.00	75.00	34.11	4.68	19.16	25.58	0.00	7.24	4.07
Overstorey	133.8974	347.9176	72.50	75.20	46.38	38.60	34.16	35.40	20.93	17.83	10.03
Overstorey	110.3229	20.5652	72.40	76.60	74.17	18.92	35.60	37.06	0.00	19.06	14.92
Overstorey	32.3411	303.4232	72.40	74.00	26.86	52.32	27.87	33.54	0.00	0.00	0.00
Overstorey	147.3026	191.2629	72.40	73.30	14.89	68.02	38.31	46.49	13.65	6.07	16.02
Overstorey	176.6574	225.9986	72.20	74.00	30.25	47.23	39.79	42.52	15.21	15.82	14.96
Overstorey	116.9173	338.3586	72.20	72.70	8.18	12.70	26.22	39.47	0.00	9.30	8.77

Overstorey	62.0208	87.0208	72.00	74.20	37.17	15.15	32.38	26.99	0.00	20.26	19.56
Overstorey	143.6979	200.1623	72.00	73.90	31.91	54.63	30.80	42.09	0.00	0.00	8.11
Overstorey	105.6418	115.0711	71.90	76.70	85.20	44.21	24.55	31.41	19.45	8.64	10.04
Overstorey	127.1276	26.6623	71.80	72.40	9.79	19.74	26.60	40.28	0.00	0.00	20.61
Overstorey	84.2047	340.2265	71.60	73.40	30.01	52.72	34.53	34.76	14.25	6.33	3.56
Overstorey	133.4748	183.7759	71.50	72.90	23.12	38.43	43.40	43.00	28.31	12.58	17.65
Overstorey	166.1035	268.2667	71.40	74.30	49.29	31.02	24.94	21.29	0.00	0.00	4.18
Overstorey	178.5120	184.1491	71.40	73.70	38.63	24.31	33.26	44.37	0.00	0.00	9.82
Overstorey	82.1225	146.7822	71.30	72.60	21.37	30.21	36.94	37.87	14.67	20.74	19.33
Overstorey	41.4853	167.2495	71.30	71.70	6.45	58.34	30.73	31.74	55.13	24.50	25.71
Overstorey	52.0318	179.7866	71.10	73.20	34.99	2.57	38.84	30.53	0.00	31.27	17.59
Overstorey	58.9170	127.6413	71.00	71.40	6.43	51.07	33.06	38.29	50.36	29.73	31.09
Overstorey	175.1014	345.9997	70.70	73.00	38.27	17.87	19.00	25.02	0.00	0.00	3.52
Overstorey	137.9501	371.0882	70.60	73.50	48.77	27.95	30.73	34.53	0.00	0.00	4.80
Overstorey	32.6792	269.6610	70.50	75.30	83.69	9.07	31.86	30.26	0.00	7.47	8.63
Overstorey	84.2178	77.2983	70.50	73.10	43.41	8.49	24.22	27.82	0.00	11.24	16.65
Overstorey	53.3983	291.6966	70.50	72.60	34.71	20.70	21.66	35.54	0.00	7.25	11.80
Overstorey	46.7661	290.6906	70.50	72.50	32.99	18.00	25.87	27.11	13.18	13.11	10.80
Overstorey	103.6902	152.3964	70.30	71.50	19.41	30.81	42.61	37.95	0.00	0.00	7.58
Overstorey	78.4857	167.8675	70.10	72.10	32.81	37.35	47.47	39.57	19.23	22.81	20.76
Overstorey	164.9107	200.9740	70.00	73.00	50.15	79.96	40.78	39.48	39.49	17.55	14.97
Overstorey	135.0275	61.9503	70.00	73.00	50.15	35.30	37.52	29.09	15.60	16.40	14.00
Overstorey	60.0370	23.6472	69.80	72.40	43.00	32.73	58.08	46.53	0.00	30.11	27.82
Overstorey	141.9084	143.5676	69.80	70.50	11.13	24.60	36.15	31.85	0.00	14.02	16.52
Overstorey	25.6541	254.6543	69.70	70.80	17.61	54.91	33.73	43.06	34.53	15.35	26.54
Overstorey	115.0309	356.9537	69.50	72.00	41.09	7.87	32.26	32.98	0.00	9.30	13.33
Overstorey	101.5802	333.9618	69.30	69.90	9.45	48.15	40.17	37.44	0.00	6.33	13.72
Overstorey	145.0718	182.8408	69.10	69.40	4.68	39.28	39.27	43.23	13.43	18.26	18.17
Overstorey	51.7097	238.8845	69.00	72.00	49.48	31.81	29.45	16.96	17.47	17.84	10.03
Overstorey	118.9921	212.7414	69.00	70.00	15.82	29.35	45.85	35.85	17.26	28.35	15.94
Overstorey	161.3300	184.6768	68.90	69.20	4.67	69.79	36.63	60.10	17.93	7.97	27.45
Overstorey	85.5554	355.4948	68.70	71.00	37.24	20.70	30.50	31.59	16.28	21.94	15.71
Overstorey	106.3401	109.9520	68.60	71.80	52.71	36.53	31.45	30.68	34.15	15.18	12.64
Overstorey	115.9569	50.5475	68.60	70.80	35.49	33.02	35.94	34.02	21.30	23.94	19.69
Overstorey	154.5958	146.7178	68.60	70.70	33.81	24.92	42.40	45.98	17.39	22.52	21.17
Overstorey	70.2433	173.5586	68.10	68.50	6.17	57.73	42.18	34.42	36.66	29.04	23.74
Overstorey	23.3620	72.9893	68.00	72.00	66.42	22.76	39.44	36.75	14.52	35.78	24.00
Overstorey	27.8942	35.0932	68.00	72.00	66.42	31.92	27.04	35.04	31.22	13.87	19.41
Overstorey	158.8095	244.8921	68.00	68.80	12.42	22.49	39.31	32.43	0.00	7.43	10.25
Overstorey	122.8630	331.9899	67.80	69.80	31.77	14.55	14.27	28.79	13.21	5.87	16.59
Overstorey	151.9571	183.4770	67.50	67.90	6.11	59.94	35.92	55.57	55.38	24.61	31.66
Overstorey	168.9013	187.5632	67.30	68.70	21.80	29.94	58.95	50.78	11.97	34.73	30.27
Overstorey	44.7239	118.6698	67.10	67.70	9.16	69.81	63.62	47.31	60.96	49.06	34.33
Overstorey	94.0832	159.0610	67.00	69.50	39.69	9.25	36.85	41.21	0.00	12.20	20.99
Overstorey	125.8876	81.7418	67.00	68.90	29.78	29.48	14.13	32.89	19.80	8.80	20.47
Overstorey	36.1016	298.6016	67.00	68.30	20.11	41.46	29.64	26.72	13.69	17.12	12.93
Overstorey	150.5839	356.6745	67.00	68.10	16.95	20.68	19.31	29.53	0.00	0.00	11.71
Overstorey	35.0195	309.3434	66.90	68.40	23.28	19.57	41.07	29.03	13.69	6.08	3.42
Overstorey	158.8568	60.1886	66.80	68.80	31.33	63.74	41.49	31.85	33.16	14.74	12.76
Overstorey	118.3219	204.8852	66.50	68.20	26.34	44.02	33.97	43.47	25.90	19.18	26.06
Overstorey	145.8018	194.7166	66.40	66.70	4.50	54.83	42.40	38.99	27.08	22.51	23.51

Overstorey	168.9814	171.7564	66.30	68.90	40.96	42.99	30.45	31.75	0.00	7.97	14.53
Overstorey	108.4036	139.7517	66.30	68.00	26.26	34.50	42.21	36.72	0.00	22.22	16.32
Overstorey	148.6727	163.2813	66.30	67.80	23.07	13.62	22.00	47.94	0.00	8.47	27.30
Overstorey	119.0993	181.8337	66.30	66.90	9.05	39.78	35.44	46.13	29.16	25.35	27.52
Overstorey	146.0746	54.0899	66.20	68.40	34.31	11.05	44.63	37.23	0.00	24.96	21.45
Overstorey	102.2721	357.6706	66.20	68.30	32.68	23.88	36.18	28.81	0.00	14.72	11.43
Overstorey	165.1958	166.3500	66.10	66.80	10.55	40.59	41.62	39.09	26.50	27.48	25.99
Overstorey	101.9854	204.9141	65.90	68.20	35.79	34.11	36.05	34.81	13.62	25.40	17.35
Overstorey	174.0335	107.2069	65.80	68.40	40.66	22.19	38.49	27.94	12.32	32.98	21.61
Overstorey	109.9694	92.1488	65.70	68.70	47.26	13.15	25.68	30.04	0.00	16.09	20.10
Overstorey	104.5702	323.8116	65.70	67.80	32.44	19.39	27.81	34.67	0.00	11.63	18.54
Overstorey	118.7229	244.4112	65.70	67.50	27.63	22.92	29.53	20.74	14.94	13.31	7.49
Overstorey	177.1011	299.4162	65.50	70.30	78.30	4.32	20.75	19.54	0.00	5.86	7.26
Overstorey	131.4820	370.5046	65.50	69.10	57.27	32.14	32.06	31.09	13.51	14.53	12.42
Overstorey	107.9112	45.9837	65.50	66.90	21.24	29.05	28.46	34.37	28.53	23.75	22.82
Overstorey	64.0393	53.9078	65.40	69.70	69.33	49.45	40.25	50.26	29.16	24.36	36.37
Overstorey	75.2024	174.0982	65.40	66.00	8.93	71.81	35.87	39.15	61.38	27.28	26.77
Overstorey	140.9440	101.6059	65.30	68.60	52.02	53.11	37.67	31.60	14.82	13.36	10.58
Overstorey	59.6677	281.0411	65.30	68.00	42.02	33.93	46.24	37.26	16.32	26.83	18.38
Overstorey	102.7812	371.0595	65.10	67.20	32.16	42.47	34.32	35.16	20.16	19.76	17.21
Overstorey	146.0871	360.4169	65.10	67.10	30.56	20.72	20.14	31.33	11.59	11.16	17.59
Overstorey	47.8354	315.2694	65.10	65.60	7.39	24.30	21.36	33.49	10.89	10.04	15.37
Overstorey	136.6914	42.9518	65.00	69.00	63.73	11.13	15.05	31.92	0.00	0.00	15.49
Overstorey	116.2130	167.0852	64.90	67.00	32.07	26.78	27.10	40.48	15.21	12.89	18.90
Overstorey	133.3018	169.7166	64.80	65.70	13.36	17.36	28.36	47.32	14.36	20.83	35.26
Overstorey	62.9875	278.9702	64.70	64.90	2.92	35.41	40.86	30.59	11.56	26.11	17.98
Overstorey	133.5607	316.1338	64.60	66.10	22.50	26.61	26.61	28.64	14.52	14.48	17.96
Overstorey	168.0024	177.3925	64.60	65.00	5.85	55.21	44.26	36.84	41.77	34.80	28.00
Overstorey	69.7982	23.7382	64.50	66.80	35.07	54.17	53.34	32.30	42.59	40.91	23.01
Overstorey	63.5716	307.6251	64.50	66.40	28.71	26.85	20.35	29.47	0.00	0.00	7.34
Overstorey	57.5206	46.4627	64.30	66.60	34.97	38.03	59.42	40.13	30.51	48.52	31.68
Overstorey	47.9313	311.9999	64.10	66.00	28.54	11.02	24.69	30.33	0.00	5.20	12.02
Overstorey	156.3204	114.0174	64.00	70.00	98.28	56.20	32.34	38.50	44.24	19.66	19.56
Overstorey	178.4059	33.2225	64.00	67.30	51.06	41.42	34.79	30.01	41.42	26.94	24.15
Overstorey	158.6992	349.4735	63.70	66.20	37.84	9.29	12.62	27.58	0.00	5.15	12.09
Overstorey	144.7774	243.6065	63.70	65.00	19.15	9.54	27.50	26.58	0.00	5.26	8.00
Overstorey	164.4449	236.0298	63.60	65.20	23.70	49.22	48.68	42.31	24.25	22.80	20.43
Overstorey	34.2480	257.4889	63.60	65.00	20.64	46.96	47.70	32.91	30.25	27.22	15.31
Overstorey	149.5628	32.1055	63.50	67.70	65.78	15.85	23.61	23.86	14.25	22.04	20.72
Overstorey	122.6143	369.3222	63.50	67.60	64.08	15.94	25.77	33.79	11.94	19.59	18.64
Overstorey	134.2348	294.8457	63.50	66.80	50.69	9.52	24.19	17.84	0.00	12.04	6.77
Overstorey	74.9511	71.2184	63.50	66.70	49.04	43.65	38.16	31.30	40.94	35.54	23.70
Overstorey	20.7457	89.1028	63.50	65.20	25.20	26.81	29.66	48.75	26.81	29.51	43.11
Overstorey	166.8000	257.4478	63.50	65.00	22.13	41.29	43.01	29.38	16.73	23.54	13.24
Overstorey	32.3296	352.6678	63.50	65.00	22.13	16.49	18.16	25.00	0.00	5.20	2.92
Overstorey	160.3231	254.5961	63.50	64.80	19.09	40.16	35.89	31.04	39.12	23.52	21.18
Overstorey	148.8158	80.7657	63.50	64.50	14.59	21.03	27.45	37.08	0.00	15.29	20.79
Overstorey	30.0780	106.0518	63.30	66.40	47.27	44.13	37.95	39.06	39.81	33.33	28.41
Overstorey	84.7761	200.6836	63.30	64.50	17.53	20.44	23.57	36.45	15.76	20.27	27.78
Overstorey	167.7229	66.0229	63.20	63.50	4.28	56.15	58.68	50.01	31.95	45.41	32.89
Overstorey	82.2402	253.8497	63.00	65.60	39.03	18.01	38.06	30.27	0.00	25.57	14.38

Overstorey	22.4433	49.7789	63.00	65.40	35.87	39.51	30.77	28.17	29.14	19.83	19.29
Overstorey	111.2215	97.6039	63.00	64.30	18.95	29.55	42.29	37.76	28.20	35.71	27.57
Overstorey	127.4295	35.5232	63.00	64.00	14.47	23.11	15.98	36.41	13.10	11.11	24.93
Overstorey	175.3922	229.4829	63.00	63.20	2.84	50.58	36.80	43.81	28.90	17.57	21.04
Overstorey	83.6565	231.2893	63.00	63.10	1.42	29.40	40.76	29.07	20.16	29.29	22.14
Overstorey	163.2471	194.0576	62.90	65.70	42.16	61.61	56.01	52.09	55.24	42.01	38.89
Overstorey	37.7827	145.5951	62.90	63.10	2.84	0.68	37.31	29.12	0.00	33.87	25.20
Overstorey	178.8000	194.0818	62.80	67.50	73.67	43.10	38.72	43.81	34.92	26.51	25.77
Overstorey	84.2085	130.6430	62.80	65.00	32.63	30.72	24.95	30.95	14.40	13.74	22.25
Overstorey	111.6183	119.7599	62.70	65.00	34.14	36.94	45.02	27.31	34.15	30.11	16.94
Overstorey	101.3000	189.7517	62.70	62.80	1.41	18.98	37.64	43.98	14.25	25.35	32.83
Overstorey	25.7321	314.1444	62.60	63.60	14.38	18.52	22.24	30.46	0.00	11.28	18.68
Overstorey	48.8375	109.5133	62.60	63.50	12.91	76.13	50.14	50.72	67.84	42.44	43.93
Overstorey	101.7370	340.7669	62.50	65.80	49.95	30.31	39.98	37.44	12.21	11.76	19.07
Overstorey	162.6149	271.3331	62.50	65.10	38.74	22.06	22.22	25.46	13.80	6.13	7.63
Overstorey	142.5185	230.5736	62.50	62.90	5.66	24.56	25.72	30.51	0.00	4.69	12.28
Overstorey	102.7170	139.8618	62.30	63.90	23.23	35.40	36.74	31.17	28.08	18.16	15.39
Overstorey	33.8705	335.5714	62.20	65.00	41.72	20.27	13.62	29.12	14.17	6.30	9.51
Overstorey	138.0578	256.3756	62.20	63.00	11.38	25.36	16.82	21.73	20.16	13.65	13.00
Overstorey	63.5876	318.0953	62.00	64.70	40.02	16.16	22.49	25.45	0.00	4.90	8.17
Overstorey	93.0360	328.9488	62.00	64.50	36.88	50.22	53.03	39.92	26.16	33.87	19.05
Overstorey	85.2390	222.0480	62.00	63.30	18.65	42.21	29.65	34.48	25.86	16.00	19.96
Overstorey	29.0083	184.9209	62.00	62.90	12.79	26.46	37.42	31.10	0.00	17.52	17.85
Overstorey	151.6738	235.5683	62.00	62.50	7.04	24.67	40.42	32.81	24.25	29.85	20.36
Overstorey	30.8422	365.3494	62.00	62.50	7.04	12.69	25.69	24.34	0.00	16.94	12.40
Overstorey	154.7566	118.1687	61.70	66.00	65.76	13.32	41.40	31.05	12.25	40.21	22.62
Overstorey	71.5664	308.1634	61.60	64.00	35.11	27.90	32.71	28.47	11.02	15.80	16.60
Overstorey	20.2274	226.6425	61.50	66.70	80.86	27.25	41.88	29.43	11.53	18.72	13.72
Overstorey	165.0218	205.6640	61.50	62.50	14.14	72.73	53.75	41.84	52.81	37.33	30.37
Overstorey	48.3399	262.0389	61.00	63.40	34.79	21.40	18.81	22.89	0.00	4.69	10.26
Overstorey	141.2471	191.6616	61.00	62.50	21.29	78.34	48.91	36.90	60.36	37.85	28.37
Overstorey	141.4382	217.9204	61.00	61.40	5.53	27.64	26.60	27.47	14.29	10.75	15.52
Overstorey	79.5572	333.2214	60.90	62.20	18.33	45.35	40.03	29.63	13.47	16.94	13.02
Overstorey	113.0331	30.0721	60.70	62.30	22.65	61.49	41.42	35.84	39.57	30.52	26.73
Overstorey	111.7031	257.3129	60.50	64.50	59.69	15.74	13.72	19.91	15.02	11.74	14.05
Overstorey	177.4930	77.8679	60.40	62.70	32.95	65.26	44.99	36.65	64.02	43.49	26.99
Overstorey	134.9010	190.3391	60.20	63.60	49.82	35.67	57.26	40.31	13.29	40.82	28.64
Overstorey	44.1908	362.8507	60.20	62.70	35.87	15.97	25.17	30.51	13.40	11.15	13.34
Overstorey	175.0000	348.4000	60.20	61.80	22.48	34.02	20.75	20.09	25.85	11.49	12.90
Overstorey	124.2017	198.4120	60.20	60.80	8.23	11.63	29.40	41.47	11.63	21.93	27.37
Overstorey	130.6769	90.4552	60.00	60.90	12.39	39.40	47.15	36.05	27.12	34.73	22.48
Overstorey	89.6531	233.6356	59.80	63.70	57.45	18.16	25.98	31.72	0.00	8.84	15.47
Overstorey	27.7348	170.4424	59.80	61.10	18.01	2.89	21.15	42.83	0.00	10.11	32.46
Overstorey	79.7508	342.7763	59.80	61.00	16.59	32.80	38.10	33.81	23.14	22.22	19.91
Overstorey	119.5720	155.7268	59.70	60.70	13.73	2.37	37.82	39.70	0.00	20.33	27.33
Overstorey	48.5531	52.3494	59.60	61.20	22.26	80.82	52.69	45.97	66.29	42.55	37.16
Overstorey	138.1500	51.1258	59.50	62.80	47.73	33.43	31.26	31.33	23.60	25.87	25.79
Overstorey	179.8637	368.7253	59.50	60.50	13.69	40.20	34.10	29.82	22.90	22.32	17.43
Overstorey	132.0900	58.0138	59.20	62.30	44.42	44.88	39.12	28.40	44.48	31.90	24.05
Overstorey	93.5073	63.5593	59.10	63.20	60.03	5.92	12.20	26.10	0.00	0.00	17.61
Overstorey	94.0410	335.8860	59.10	59.30	2.67	69.42	46.57	41.87	56.99	35.61	28.72

Overstorey	135.5626	149.6508	59.00	61.30	32.23	82.73	48.13	37.15	50.71	29.33	22.50
Overstorey	122.2693	110.8499	59.00	60.20	16.37	15.84	37.36	39.92	0.00	27.40	30.07
Overstorey	106.1560	206.8369	58.90	60.30	19.16	36.40	51.41	43.62	25.28	41.18	36.56
Overstorey	175.1710	254.8970	58.80	60.00	16.32	41.91	29.38	34.12	27.29	16.79	15.86
Overstorey	173.6900	214.0727	58.80	59.70	12.15	28.35	52.05	45.41	20.38	39.70	32.70
Overstorey	109.3126	131.4004	58.50	63.00	65.89	34.77	26.28	34.82	32.26	23.57	24.89
Overstorey	142.8043	85.8754	58.50	60.80	31.97	25.22	39.70	45.97	25.22	24.03	29.50
Overstorey	63.4848	25.1736	58.50	59.80	17.63	60.12	58.84	46.73	59.08	56.23	42.52
Overstorey	114.2672	195.8143	58.50	59.10	8.00	25.28	51.17	38.74	25.28	41.27	32.83
Overstorey	82.1418	157.1418	58.50	59.00	6.65	21.90	38.95	37.18	14.67	30.40	28.69
Overstorey	75.0873	179.9992	58.50	58.70	2.64	43.89	39.48	27.00	41.85	32.12	21.51
Overstorey	41.5696	74.3190	58.40	61.80	48.45	21.79	31.32	35.98	17.02	27.36	30.23
Overstorey	101.2665	296.5114	58.40	61.50	43.86	5.59	16.07	24.82	0.00	7.43	17.98
Overstorey	139.4563	237.9158	58.40	60.10	23.25	27.40	21.87	23.54	20.45	13.43	13.77
Overstorey	129.2441	304.0985	58.40	60.00	21.83	12.36	17.87	29.66	0.00	9.81	20.44
Overstorey	40.2444	299.3873	58.40	59.20	10.70	30.17	35.77	27.64	25.35	31.35	23.62
Overstorey	120.2748	33.3374	58.20	61.00	39.21	33.24	35.82	39.27	33.05	32.86	33.50
Overstorey	49.0078	241.2391	58.20	60.00	24.60	40.15	21.08	21.93	30.43	13.53	13.27
Overstorey	145.7522	221.3494	58.20	59.30	14.77	33.60	29.16	29.93	33.60	25.71	19.16
Overstorey	152.8354	100.2895	58.10	60.20	28.86	22.69	51.77	48.82	0.00	33.95	33.30
Overstorey	142.0344	66.7284	57.90	61.50	51.13	29.33	31.17	33.92	28.92	30.85	28.89
Overstorey	41.2696	119.4427	57.90	59.50	21.65	72.30	61.38	45.43	72.14	58.63	38.92
Overstorey	29.0854	298.1428	57.70	61.50	54.06	30.21	28.52	30.86	25.35	22.29	21.24
Overstorey	128.4415	342.4149	57.70	58.00	3.91	52.60	34.95	32.07	35.07	26.87	26.20
Overstorey	170.9889	224.6234	57.40	59.80	32.85	51.84	62.24	39.54	38.88	41.85	25.98
Overstorey	143.2894	357.2783	57.40	58.70	17.31	24.48	34.88	37.10	22.85	30.97	28.37
Overstorey	75.3882	238.9489	57.40	58.30	11.86	32.21	42.02	37.67	31.54	36.85	32.83
Overstorey	141.3192	104.0956	57.30	59.70	32.80	27.10	39.73	35.19	11.76	22.62	23.14
Overstorey	78.1211	234.6057	57.30	59.60	31.35	19.00	36.87	43.22	9.95	32.29	32.70
Overstorey	168.4756	140.9500	57.20	59.00	24.19	21.84	39.50	37.44	15.37	29.49	28.13
Overstorey	139.9283	156.6718	57.20	58.20	13.17	76.35	53.25	33.33	47.67	36.61	23.72
Overstorey	30.4518	249.1914	57.20	57.80	7.82	49.95	31.23	31.71	40.82	26.59	21.47
Overstorey	175.0648	377.4424	57.00	59.00	26.93	30.83	35.45	25.06	28.64	22.91	15.24
Overstorey	50.9384	345.1426	56.90	59.20	31.15	19.49	27.15	33.38	11.70	18.20	22.23
Overstorey	103.7094	55.9363	56.90	58.80	25.47	3.46	24.86	20.03	0.00	22.34	16.57
Overstorey	51.2958	376.3896	56.80	61.30	64.17	21.36	24.05	26.08	18.49	14.17	17.73
Overstorey	21.3428	188.5075	56.80	59.50	36.87	21.63	31.24	28.80	9.89	21.37	20.55
Overstorey	83.1021	56.6788	56.80	58.50	22.64	41.95	29.08	23.97	40.51	22.44	18.44
Overstorey	144.3573	154.1203	56.80	57.80	13.08	51.72	53.71	43.32	36.91	41.14	33.84
Overstorey	176.1212	21.6314	56.50	60.40	54.56	41.42	36.46	27.84	41.42	32.95	25.62
Overstorey	148.3799	96.4687	56.50	57.60	14.35	50.04	42.23	46.66	35.65	32.61	34.63
Overstorey	106.3726	155.1604	56.50	56.90	5.13	40.59	37.43	40.41	12.78	15.14	24.35
Overstorey	144.8421	159.7434	56.20	57.00	10.30	52.87	39.19	44.76	47.36	33.77	39.52
Overstorey	173.1066	273.1066	56.00	59.40	46.62	26.21	20.77	23.89	13.80	10.84	10.99
Overstorey	172.2704	199.1439	56.00	57.20	15.56	55.82	55.96	48.64	55.82	50.74	43.26
Overstorey	128.4059	238.9457	55.90	59.00	42.12	28.57	22.06	23.01	14.94	15.71	16.43
Overstorey	137.9286	379.0775	55.90	58.20	30.63	43.80	42.91	33.06	13.51	28.51	21.25
Overstorey	41.7684	259.1537	55.70	57.80	27.73	33.69	31.32	32.82	20.61	23.34	18.51
Overstorey	75.9576	141.0739	55.70	56.20	6.33	13.34	33.94	46.05	13.18	30.35	40.11
Overstorey	78.3267	279.7511	55.60	59.50	53.78	27.92	45.54	35.05	9.09	26.78	20.59
Overstorey	53.1931	107.0067	55.60	57.60	26.30	42.74	51.11	33.89	42.74	46.92	31.44

Overstorey	168.0352	166.0946	55.60	56.00	5.04	29.27	39.07	41.64	23.02	29.72	31.42
Overstorey	138.4721	196.7491	55.50	56.80	16.76	56.90	43.30	34.87	44.65	35.83	29.53
Overstorey	65.3739	221.3448	55.50	55.90	5.04	14.99	33.03	37.32	14.48	27.35	33.75
Overstorey	168.0626	290.4577	55.30	59.30	55.02	14.43	21.74	19.60	0.00	12.74	12.02
Overstorey	113.1906	184.8759	55.30	55.90	7.57	44.34	40.63	41.71	40.99	39.01	37.53
Overstorey	37.3528	51.0807	55.20	56.80	20.68	19.79	43.55	37.11	19.58	32.30	28.70
Overstorey	31.6351	242.1460	55.20	56.80	20.68	28.62	29.49	30.07	27.36	25.61	26.47
Overstorey	107.2532	354.6208	55.20	56.10	11.42	33.61	27.01	35.52	24.62	19.89	29.59
Overstorey	123.8206	347.3409	55.10	56.60	19.30	30.88	43.77	29.94	29.34	39.48	25.25
Overstorey	163.7553	60.2232	54.80	55.20	4.97	44.16	46.12	43.69	21.91	35.91	37.72
Overstorey	105.3117	235.4248	54.50	56.80	29.91	11.54	13.40	23.28	8.24	11.31	19.39
Overstorey	21.0717	217.6504	54.20	56.60	31.13	43.37	27.57	29.99	22.65	17.79	21.60
Overstorey	144.2103	97.4485	54.10	55.20	13.76	59.58	36.39	37.92	52.85	27.60	29.15
Overstorey	60.0077	95.3189	54.00	57.40	45.09	34.06	33.34	26.54	34.06	30.16	24.10
Overstorey	107.8006	236.1405	54.00	57.40	45.09	10.99	24.88	23.25	0.00	19.36	17.33
Overstorey	171.5945	287.8176	54.00	56.20	28.29	14.27	17.62	23.56	8.79	13.32	17.51
Overstorey	159.1944	50.3211	54.00	56.00	25.58	18.80	25.90	29.67	11.83	18.94	21.49
Overstorey	109.8481	164.2365	53.90	54.80	11.16	35.87	41.48	39.72	26.91	34.63	28.94
Overstorey	69.1514	276.4138	53.90	54.60	8.63	42.13	34.05	33.01	19.38	20.27	23.70
Overstorey	72.9207	54.1048	53.50	57.60	54.88	54.17	28.82	34.22	52.66	27.21	32.06
Overstorey	50.7844	58.9658	53.50	54.30	9.82	63.23	49.25	45.15	56.08	43.95	41.84
Overstorey	93.0000	259.5263	53.30	58.00	63.65	19.00	17.66	26.48	16.12	11.95	18.25
Overstorey	137.9325	143.6849	53.20	55.80	33.31	29.86	45.33	33.74	21.82	40.98	28.21
Overstorey	173.5322	58.6193	53.10	54.70	19.93	40.26	55.93	44.17	40.26	50.65	39.00
Overstorey	104.3362	352.8701	53.10	54.70	19.93	21.04	28.88	40.08	19.53	23.38	36.28
Overstorey	141.4837	314.8000	53.10	54.70	19.93	38.73	33.88	27.92	33.31	26.84	21.42
Overstorey	100.1675	147.0985	53.00	54.10	13.49	53.61	46.77	35.75	47.16	38.43	29.27
Overstorey	90.8759	145.0824	52.70	55.30	33.02	37.24	44.77	41.86	29.70	39.17	31.56
Overstorey	80.1325	129.3067	52.70	54.00	15.94	26.15	39.00	40.29	24.96	37.86	39.17
Overstorey	94.8388	371.0926	52.70	53.70	12.16	44.58	38.10	39.14	31.45	27.97	30.24
Overstorey	106.2864	159.8677	52.60	55.10	31.61	37.13	48.77	36.86	20.87	29.86	24.25
Overstorey	77.5910	159.0359	52.60	53.80	14.65	36.37	36.12	40.57	36.37	36.12	37.68
Overstorey	67.1056	338.4370	52.50	56.50	52.51	29.87	30.77	29.52	19.23	16.98	17.61
Overstorey	167.6405	180.3231	52.50	53.60	13.37	70.38	50.56	44.56	64.13	47.78	39.78
Overstorey	155.5565	141.9199	52.30	54.90	32.79	15.75	41.93	38.03	12.50	37.61	33.61
Overstorey	29.8852	180.8219	52.30	53.20	10.83	20.18	29.78	31.73	17.05	23.93	26.53
Overstorey	133.0301	373.9681	52.10	53.70	19.57	41.92	29.17	39.50	41.92	27.24	34.77
Overstorey	152.3170	335.0360	52.00	54.00	24.68	24.73	23.90	24.64	12.18	16.15	17.19
Overstorey	88.5299	332.3280	52.00	53.60	19.53	69.25	49.14	38.02	56.58	40.91	30.92
Overstorey	165.9510	235.0225	51.90	53.10	14.46	20.43	56.35	41.23	10.63	49.97	34.31
Overstorey	21.0529	25.8989	51.90	52.20	3.52	37.74	29.55	33.23	36.86	28.16	31.00
Overstorey	79.9847	377.3244	51.80	54.40	32.50	29.24	25.11	32.21	28.92	20.09	22.85
Overstorey	75.0995	311.3996	51.70	54.20	31.11	26.62	31.44	26.36	10.24	20.35	14.93
Overstorey	50.5603	51.4987	51.40	53.50	25.71	56.98	59.23	42.56	56.98	58.97	39.48
Overstorey	155.2914	81.6522	51.40	51.80	4.67	41.39	31.91	44.46	29.98	23.68	37.40
Overstorey	92.1698	128.0908	51.20	54.60	42.95	12.71	17.38	34.40	10.56	12.04	30.97
Overstorey	117.4253	281.3041	51.10	56.20	67.23	22.88	15.50	26.53	13.69	6.08	17.92
Overstorey	41.7262	321.5631	51.10	53.00	23.00	21.64	30.85	27.67	10.76	21.12	21.74
Overstorey	87.6745	272.4985	51.00	55.00	51.16	12.64	30.90	32.78	9.09	27.77	23.52
Overstorey	27.7721	181.8612	51.00	53.50	30.71	29.01	38.96	24.08	18.74	29.99	16.87
Overstorey	140.5543	205.5101	51.00	52.50	17.92	27.08	36.34	33.69	21.72	29.47	26.97

Overstorey	129.1203	219.3573	51.00	51.90	10.57	13.41	23.35	34.43	0.00	17.31	29.25
Overstorey	141.1773	148.8176	51.00	51.70	8.18	65.48	47.74	41.42	65.48	46.81	39.26
Overstorey	166.2558	344.5636	50.90	54.40	44.10	35.14	18.38	24.59	33.83	15.03	18.00
Overstorey	165.2658	106.2121	50.70	52.70	24.10	56.53	36.03	37.00	55.94	34.33	31.95
Overstorey	67.8553	264.1373	50.60	52.50	22.79	9.71	22.54	32.18	0.00	12.88	20.18
Overstorey	104.5197	316.2924	50.60	51.00	4.60	29.13	30.07	26.75	28.22	26.77	22.78
Overstorey	83.0301	310.8521	50.50	52.33	21.86	28.18	25.81	17.94	21.41	20.26	14.15
Overstorey	112.1353	146.3168	50.30	51.40	12.82	20.22	41.20	37.65	11.56	36.47	34.18
Overstorey	48.4983	137.6920	50.20	52.10	22.62	0.00	38.27	34.93	0.00	36.21	33.47
Overstorey	161.0434	197.7982	50.20	50.50	3.41	52.03	49.19	50.57	52.03	46.81	47.89
Overstorey	68.7294	379.6942	50.10	54.30	53.15	29.10	25.18	31.08	18.49	18.30	25.64
Overstorey	123.3614	245.6847	50.10	51.60	17.62	35.69	23.13	29.68	35.03	22.24	26.95
Overstorey	107.4719	149.4575	50.10	50.80	8.03	47.71	37.23	42.99	46.36	35.94	38.39
Overstorey	70.8439	208.3439	50.00	52.50	30.15	61.81	52.10	39.51	60.92	51.46	34.05
Overstorey	42.7616	276.2147	49.90	54.70	61.50	26.57	21.09	29.23	16.32	13.55	21.69
Overstorey	103.7050	31.2899	49.80	52.20	28.76	58.34	41.26	30.36	51.41	36.48	25.64
Overstorey	135.1029	233.1812	49.80	51.40	18.74	31.03	25.18	30.00	27.62	23.61	26.47
Overstorey	24.9724	55.1662	49.80	50.90	12.70	43.25	31.82	33.48	39.35	28.17	27.61
Overstorey	165.1400	172.3524	49.70	50.00	3.38	66.54	34.89	38.91	66.54	34.89	38.91
Overstorey	54.0016	85.9944	49.60	51.90	27.38	25.26	22.55	20.20	25.09	22.37	20.05
Overstorey	63.4872	322.7526	49.50	51.10	18.63	11.21	12.22	31.14	10.46	4.65	23.95
Overstorey	162.6526	64.9953	49.50	49.90	4.50	52.13	43.01	49.21	43.59	39.14	45.63
Overstorey	58.3742	336.9596	49.40	54.50	65.29	43.58	24.06	28.94	37.22	20.44	20.58
Overstorey	167.6683	89.3811	49.40	51.30	22.28	2.41	19.15	35.64	0.00	11.68	30.58
Overstorey	177.3811	333.8865	49.30	52.00	32.32	19.87	25.51	20.50	12.04	21.77	14.09
Overstorey	166.9796	149.1698	49.30	50.90	18.56	59.38	47.52	35.29	59.38	45.19	32.02
Overstorey	90.7801	223.9390	49.00	50.60	18.45	21.12	28.72	28.05	20.16	24.88	25.54
Overstorey	44.0366	264.3743	49.00	50.20	13.68	25.18	27.19	27.84	25.03	25.44	22.82
Overstorey	78.5923	307.7036	48.80	51.10	26.97	38.66	20.92	28.03	38.49	17.11	22.53
Overstorey	176.0566	363.4185	48.80	50.00	13.63	27.02	36.31	29.73	21.68	30.25	22.73
Overstorey	132.9794	96.0568	48.50	50.20	19.47	62.83	47.04	41.75	51.11	41.46	35.58
Overstorey	155.6573	90.8993	48.20	50.30	24.20	44.16	37.06	36.35	43.64	34.44	34.47
Overstorey	134.8989	99.2971	47.80	48.30	5.45	79.82	39.87	39.75	73.93	36.55	34.46
Overstorey	166.4754	269.6719	47.70	50.20	28.86	38.87	23.41	25.03	33.22	19.46	21.97
Overstorey	96.0921	378.5590	47.40	48.80	15.55	49.20	44.63	43.18	40.01	36.35	35.46
Overstorey	107.2955	204.9209	47.00	47.90	9.76	39.75	52.90	43.59	34.37	49.11	41.02
Overstorey	74.3190	254.0696	46.70	49.90	36.98	42.94	22.69	37.31	32.29	17.41	30.91
Overstorey	59.9433	312.2789	46.70	49.00	25.88	29.09	32.21	23.02	21.49	26.62	18.44
Overstorey	25.3402	268.9911	46.70	47.30	6.41	37.90	40.87	38.12	37.46	38.86	36.37
Overstorey	162.5000	368.1000	46.60	49.40	31.92	15.11	17.80	31.85	0.00	6.97	23.92
Overstorey	89.5338	289.8396	46.50	49.50	34.33	24.72	36.30	35.62	20.29	32.77	30.17
Overstorey	136.2468	340.7250	46.50	48.60	23.40	24.81	32.67	35.58	22.55	28.30	31.04
Overstorey	43.9359	268.5016	46.40	51.50	61.86	32.62	34.23	22.01	25.88	29.75	16.73
Overstorey	20.9956	265.7611	46.40	50.90	53.67	42.38	34.69	33.24	36.08	31.29	29.65
Overstorey	100.8660	200.5000	46.30	46.40	1.04	54.61	39.28	39.59	54.32	38.47	36.45
Overstorey	163.2587	217.8569	46.20	47.00	8.51	9.06	51.41	56.18	0.00	45.22	52.70
Overstorey	131.1806	215.7863	46.20	47.00	8.51	14.62	26.87	34.66	6.73	23.36	32.56
Overstorey	37.6491	39.3941	46.10	50.00	45.46	18.84	18.00	37.32	16.89	16.85	35.66
Overstorey	160.2822	55.5828	46.00	47.60	17.38	34.23	44.63	31.01	33.52	42.64	29.62
Overstorey	165.6147	210.8623	46.00	46.30	3.13	37.52	54.89	51.36	37.52	53.73	49.78
Overstorey	145.9316	199.5415	46.00	46.30	3.13	62.93	42.55	44.52	62.93	40.53	42.24

Overstorey	32.6578	313.3049	45.90	48.50	29.05	39.38	24.52	34.19	35.50	22.79	29.25
Overstorey	58.1618	366.0489	45.70	50.00	50.32	39.96	27.90	23.50	31.88	22.71	19.32
Overstorey	33.2535	353.5887	45.70	47.00	13.90	21.37	24.77	28.01	10.56	18.60	20.05
Overstorey	120.6083	62.0210	45.50	47.70	24.09	38.71	39.94	26.02	37.86	39.56	24.42
Overstorey	48.4802	324.2748	45.10	47.30	23.89	24.37	27.16	27.43	17.78	23.49	24.93
Overstorey	96.8092	331.0455	44.70	45.80	11.44	43.21	50.72	37.43	38.59	47.41	34.08
Overstorey	179.6807	141.8648	44.70	45.80	11.44	22.79	29.69	25.51	22.79	27.50	22.97
Overstorey	57.5133	252.4412	44.70	44.90	2.02	5.89	34.46	34.61	0.00	31.56	31.66
Overstorey	39.6887	326.9026	44.50	46.00	15.74	26.79	30.59	29.98	26.79	25.83	26.37
Overstorey	75.1859	329.6173	44.40	47.00	28.18	13.04	28.79	40.32	9.67	24.06	36.50
Overstorey	109.7574	29.1417	44.30	47.40	34.05	57.14	45.26	37.79	56.08	43.64	35.26
Overstorey	157.9590	79.4118	44.30	45.10	8.17	36.69	29.80	40.04	36.69	28.29	39.04
Overstorey	73.2309	282.5139	44.20	47.20	32.78	38.83	43.89	36.89	30.97	34.82	29.81
Overstorey	176.3377	326.2044	44.00	48.10	46.14	21.46	17.66	21.67	18.80	14.61	17.77
Overstorey	77.3492	250.8551	44.00	45.70	17.76	43.82	32.97	36.28	38.51	29.73	32.46
Overstorey	101.5154	163.7272	43.75	44.30	5.50	52.91	34.36	39.33	49.07	29.71	36.32
Overstorey	126.4236	296.4880	43.70	47.30	39.66	23.51	15.30	20.91	20.16	8.96	13.79
Overstorey	152.4522	374.5531	43.50	45.60	21.98	25.36	25.19	36.38	18.27	16.84	30.12
Overstorey	92.1460	169.1351	43.50	44.70	12.20	1.86	29.15	45.84	0.00	23.69	41.40
Overstorey	30.3049	371.3288	43.50	44.40	9.06	29.05	22.89	25.53	27.49	17.32	21.94
Overstorey	21.4945	212.8141	43.00	45.50	26.23	28.02	36.38	27.92	25.49	33.12	25.16
Overstorey	157.7883	365.6467	43.00	45.10	21.75	10.79	20.63	28.29	6.10	17.48	25.73
Overstorey	39.5905	65.4308	42.80	43.70	8.92	62.17	42.05	47.77	62.17	42.05	46.68
Overstorey	165.5603	21.3879	42.70	43.60	8.90	45.34	33.07	38.04	41.19	30.89	36.81
Overstorey	60.8550	306.7830	42.60	43.70	10.92	22.64	27.77	29.39	17.02	24.87	27.29
Overstorey	127.6431	103.2640	42.60	43.60	9.90	58.73	42.64	43.38	52.84	36.79	38.70
Overstorey	50.0839	250.0545	42.50	43.60	10.90	15.17	30.56	35.20	14.04	27.94	33.49
Overstorey	27.9764	336.1081	42.40	43.70	12.94	11.28	32.33	26.14	10.56	30.18	21.00
Overstorey	143.7508	186.5125	42.40	42.70	2.88	68.00	54.54	46.11	68.00	54.54	46.07
Overstorey	43.4099	291.9534	42.40	42.60	1.92	47.29	31.68	32.22	46.74	30.13	30.42
Overstorey	30.4163	294.6877	42.00	43.50	14.89	35.06	21.74	30.12	34.81	19.36	27.91
Overstorey	153.5708	367.0705	42.00	43.30	12.82	16.38	29.05	27.40	16.38	27.75	26.06
Overstorey	103.1031	327.0152	42.00	43.00	9.76	37.22	39.58	42.78	37.22	38.97	41.60
Overstorey	171.9818	264.0018	42.00	43.00	9.76	48.63	39.05	29.61	48.48	38.36	27.71
Overstorey	38.4203	130.2195	41.80	42.00	1.89	41.95	31.24	34.17	40.52	30.05	33.32
Overstorey	164.1989	313.7343	41.40	46.00	49.86	7.25	14.28	23.21	0.00	7.24	16.34
Overstorey	79.3939	252.9637	41.40	42.80	13.67	23.71	40.51	29.80	22.20	39.22	28.11
Overstorey	93.2046	155.3196	41.40	41.70	2.82	12.08	52.65	44.69	12.08	52.65	44.17
Overstorey	81.5749	338.0752	41.30	43.10	17.77	61.67	42.06	34.76	53.87	38.48	31.92
Overstorey	68.0753	272.5581	41.10	43.30	21.92	37.37	28.64	34.95	24.87	22.60	30.23
Overstorey	155.5316	337.4242	41.00	44.40	35.17	24.05	27.57	27.71	19.47	23.06	23.38
Overstorey	65.6829	272.2959	41.00	42.70	16.61	34.26	28.62	29.06	29.56	24.68	25.68
Overstorey	117.7709	106.2816	40.70	42.00	12.44	50.02	54.11	38.29	44.91	50.40	34.33
Overstorey	53.1721	213.9792	40.60	42.20	15.44	22.05	22.27	19.63	21.21	19.31	16.21
Overstorey	168.3510	368.6657	40.50	45.50	53.85	12.35	28.43	29.07	12.35	22.09	24.94
Overstorey	135.2705	214.6831	40.50	42.70	21.62	21.68	18.08	37.28	21.68	16.61	36.40
Overstorey	120.1759	108.8301	40.40	41.80	13.35	19.80	48.21	38.41	18.22	45.82	35.44
Overstorey	46.8830	238.9861	40.30	41.70	13.32	22.77	21.26	23.14	21.96	19.64	22.07
Overstorey	127.8789	274.0465	40.20	42.30	20.43	25.25	23.19	23.82	21.30	19.06	19.51
Overstorey	178.0429	231.5254	39.80	41.90	20.24	55.92	43.51	52.44	55.92	39.87	48.46
Overstorey	87.8446	210.0353	39.60	39.80	1.79	24.02	36.66	37.61	24.02	36.47	36.44

Overstorey	34.8112	358.5288	39.50	43.00	35.15	29.91	27.39	28.67	25.85	23.25	25.61
Overstorey	61.0262	357.4373	39.40	42.60	31.74	9.58	22.72	28.11	6.25	19.33	25.06
Overstorey	119.0351	377.5086	39.10	40.00	8.17	11.42	29.90	32.53	11.42	28.76	31.89
Overstorey	142.6154	256.7884	39.00	41.00	18.85	31.11	19.81	21.93	30.08	18.06	20.37
Overstorey	27.7055	74.7037	39.00	40.70	15.85	39.95	37.17	40.37	39.95	35.35	39.35
Overstorey	28.4089	320.9374	39.00	39.40	3.55	24.75	27.23	28.93	24.75	27.12	28.86
Overstorey	56.9057	344.1687	38.80	43.00	42.50	37.01	33.87	21.14	35.41	28.94	16.28
Overstorey	109.5829	168.4881	38.60	39.20	5.32	45.03	37.13	43.13	45.03	36.85	40.47
Overstorey	162.9884	226.4183	38.20	38.50	2.60	73.85	48.90	50.51	71.60	46.69	48.31
Overstorey	64.5362	278.6734	38.00	40.90	27.56	47.67	50.80	32.27	44.36	47.68	30.12
Overstorey	25.8780	108.3540	37.60	38.50	7.87	39.83	26.05	36.27	38.82	24.97	34.51
Overstorey	163.7784	244.0769	36.50	37.90	12.13	37.88	39.24	43.01	37.63	37.86	41.98
Overstorey	106.5619	184.1995	36.50	36.60	0.82	32.83	49.42	43.94	32.26	48.84	42.92
Overstorey	66.1070	299.6770	36.20	36.70	4.15	32.00	35.34	34.85	32.00	34.12	32.90
Overstorey	92.0853	358.9992	36.00	39.10	28.28	13.41	46.01	38.79	12.60	45.00	37.48
Overstorey	133.6036	224.7873	35.40	35.70	2.41	18.86	27.10	32.17	18.86	26.91	32.00
Overstorey	159.4990	132.9706	35.00	36.10	9.05	41.53	28.12	36.40	41.53	27.88	35.94
Overstorey	164.7904	29.2183	35.00	35.50	4.01	44.59	39.22	34.05	43.74	38.03	33.23
Overstorey	164.5075	290.3670	34.50	39.70	49.36	19.46	18.62	21.18	16.69	14.67	16.19
Overstorey	73.7366	280.0029	34.10	35.30	9.67	46.22	44.83	36.78	45.41	43.27	34.56
Overstorey	38.9289	175.8250	34.10	34.80	5.52	51.16	45.56	35.83	50.09	43.08	33.36
Overstorey	35.6593	275.9326	33.90	35.70	14.78	22.26	18.23	28.87	21.65	17.86	28.59
Overstorey	79.7273	345.8554	33.50	33.70	1.52	31.81	34.15	37.85	27.41	31.96	35.51
Overstorey	160.4633	66.2053	33.20	33.90	5.38	68.91	43.52	37.92	68.91	43.36	37.78
Overstorey	126.4345	175.4386	32.80	33.50	5.32	51.55	40.00	33.05	51.55	40.00	32.75
Overstorey	56.0941	284.7526	32.80	33.30	3.76	51.71	39.35	35.52	51.59	39.16	35.14
Overstorey	84.9802	331.4812	32.20	32.60	2.94	51.15	46.75	38.38	50.89	45.52	37.55
Overstorey	103.5267	217.3541	31.90	32.00	0.72	16.19	36.12	43.15	15.64	35.26	42.45
Overstorey	70.3240	189.5964	31.50	33.70	17.18	11.25	27.13	42.42	6.86	23.24	38.85
Overstorey	39.0657	189.8213	31.20	34.60	27.69	44.00	33.24	30.04	39.41	29.58	25.85
Overstorey	174.5432	217.1229	30.90	32.30	10.37	73.23	49.76	51.16	73.23	49.76	50.60
Overstorey	61.1110	196.1808	30.80	35.50	39.92	20.48	22.06	30.04	14.44	16.69	24.81
Overstorey	111.4681	370.5301	30.60	32.00	10.27	18.31	34.16	35.05	18.31	34.16	35.05
Overstorey	167.7709	231.2816	29.70	30.00	2.03	79.77	51.26	47.00	79.77	51.15	46.89
Overstorey	178.0482	374.7257	29.30	29.60	2.00	44.79	37.41	35.21	44.79	37.33	34.60
Overstorey	71.7873	350.4867	29.10	29.80	4.74	3.51	27.68	34.76	2.84	26.14	33.44
Overstorey	76.0825	340.9334	28.90	29.50	4.01	48.04	41.19	33.72	47.91	40.79	33.10
Overstorey	152.1667	378.8296	27.70	28.90	7.94	23.79	24.58	37.10	23.47	23.38	36.31
Overstorey	58.7691	357.8957	27.50	30.50	21.54	25.19	33.12	29.10	24.18	30.44	26.55
Overstorey	91.9945	171.3210	27.00	27.30	1.85	20.46	34.82	44.04	19.43	34.21	43.22
Overstorey	32.2125	357.2125	26.60	27.00	2.44	32.53	27.43	27.45	30.47	24.41	24.58
Overstorey	54.9451	375.7390	26.20	27.40	7.54	28.87	30.51	25.58	27.88	30.07	24.91
Overstorey	110.4306	78.7964	25.70	30.20	32.77	4.04	17.10	23.21	0.00	14.04	19.01
